# Virtual reality-empowered deep-learning analysis of brain cells

**DOI:** 10.1038/s41592-024-02245-2

**Published:** 2024-04-22

**Authors:** Doris Kaltenecker, Rami Al-Maskari, Moritz Negwer, Luciano Hoeher, Florian Kofler, Shan Zhao, Mihail Todorov, Zhouyi Rong, Johannes Christian Paetzold, Benedikt Wiestler, Marie Piraud, Daniel Rueckert, Julia Geppert, Pauline Morigny, Maria Rohm, Bjoern H. Menze, Stephan Herzig, Mauricio Berriel Diaz, Ali Ertürk

**Affiliations:** 1 Institute for Diabetes and Cancer (IDC), Helmholtz Munich, Neuherberg, Germany; 2 Joint Heidelberg-IDC Translational Diabetes Program, Heidelberg University Hospital, Heidelberg, Germany; 3 German Center for Diabetes Research (DZD), Neuherberg, Germany; 4 Institute for Stroke and Dementia Research, Klinikum der Universität München, Ludwig-Maximilians-Universität LMU, Munich, Germany; 5 Institute for Tissue Engineering and Regenerative Medicine, Helmholtz Munich, Neuherberg, Germany; 6 Department of Computer Science, TUM Computation, Information and Technology, Technical University of Munich (TUM), Munich, Germany; 7 Center for Translational Cancer Research of the TUM (TranslaTUM), Munich, Germany; 8 Department of Diagnostic and Interventional Neuroradiology, School of Medicine, Klinikum rechts der Isar, Technical University of Munich, Munich, Germany; 9 Helmholtz AI, Helmholtz Munich, Neuherberg, Germany; 10 Department of Computing, Imperial College London, London, United Kingdom; 11 Department for Quantitative Biomedicine, University of Zurich, Zurich, Switzerland; 12 Chair Molecular Metabolic Control, TU Munich, Munich, Germany; 13 School of Medicine, Koç University, İstanbul, Turkey; 14 Munich Cluster for Systems Neurology (SyNergy), Munich, Germany; 15 Deep Piction, Munich, Germany

**Keywords:** Neuroscience, Fluorescence imaging, Software, Machine learning

## Abstract

Automated detection of specific cells in three-dimensional datasets such as whole-brain light-sheet image stacks is challenging. Here, we present DELiVR, a virtual reality-trained deep-learning pipeline for detecting c-Fos^+^ cells as markers for neuronal activity in cleared mouse brains. Virtual reality annotation substantially accelerated training data generation, enabling DELiVR to outperform state-of-the-art cell-segmenting approaches. Our pipeline is available in a user-friendly Docker container that runs with a standalone Fiji plugin. DELiVR features a comprehensive toolkit for data visualization and can be customized to other cell types of interest, as we did here for microglia somata, using Fiji for dataset-specific training. We applied DELiVR to investigate cancer-related brain activity, unveiling an activation pattern that distinguishes weight-stable cancer from cancers associated with weight loss. Overall, DELiVR is a robust deep-learning tool that does not require advanced coding skills to analyze whole-brain imaging data in health and disease.

## Abstract (web summary)

Generating training data for training deep-learning-based tools is time consuming. The DELiVR pipeline facilitates this process as demonstrated in this study on detecting c-Fos^+^ cells or microglia in the brain, following tissue clearing and imaging with light-sheet microscopy.

## Main

Analyzing the expression of proteins is essential to understand cellular and molecular processes in physiology and disease. While standard immunohistochemistry is useful for validating protein expression on tissue sections, it does not provide a holistic view of expression patterns in larger samples and information can be lost during slicing^1,2^. Tissue clearing and fluorescent imaging solve many of these restrictions and allow unbiased protein expression analysis up to the whole-organism scale^1,3–5^.

Whole-brain analysis is essential for detecting areas involved in specific behaviors or conditions. A brain-wide snapshot of the neuronal activity of an animal can be obtained by immunostaining for the expression of immediate early genes such as *c-Fos*. Unbiased quantification methods for system-level examination at the single-cell resolution are essential to interpret those brain-wide findings^6^, but current automated methods for cell detection and registration to the Allen Mouse Brain Atlas^7–9^ are difficult to apply consistently to three-dimensional (3D) whole-brain datasets. Variations in image acquisitions between samples, uneven signal-to-noise ratios across the tissue or low abundance of the target protein limit detection sensitivity and specificity. This requires manual adjustments such as setting sample and volume-specific thresholds or using conservative thresholds that will not capture all information in each sample. Deep-learning-based cell detection methods offer a promising solution to address these challenges; however, their implementation typically demands advanced coding skills, presenting a challenge for users lacking computational expertise.

**Fig. 1:**
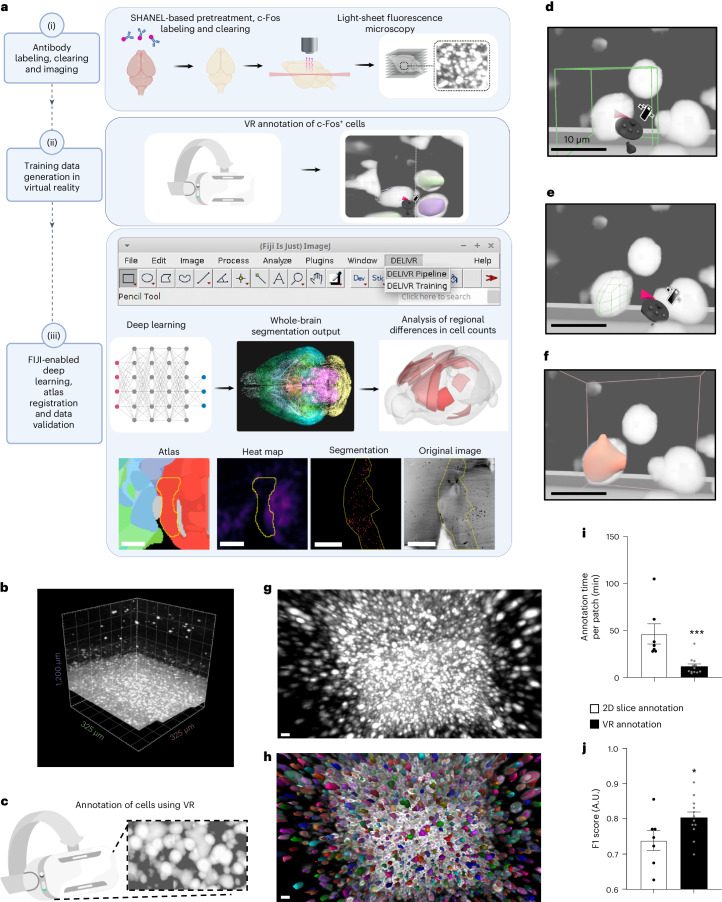
Virtual reality-aided annotation is faster than 2D-slice annotation. **a**, Summary of VR-aided deep learning for antibody-labeled cell segmentation in mouse brains. (i) Fixed mouse brains are subjected to SHANEL-based antibody labeling, tissue clearing and fluorescent light-sheet imaging. (ii) Volumes of raw data are labeled in VR to generate reference annotations. (iii) The DELiVR pipeline was packaged in a Docker container, controlled via a Fiji plugin. DELiVR segments cells using deep learning and registers them to the Allen Brain Atlas. DELiVR produces per-region cell counts and generates visualizations with all detected cells color coded by atlas region. **b**, Patch volume of raw data (c-Fos-labeled brain imaged with LSFM) and loaded into Arivis VisionVR. Volume size represents 200^3^ voxel, rendered isotropically. **c**, Illustration of VR goggles and VR zoomed-in view of the same data as in **b**. **d**–**f**, Using Arivis VisionVR, individual cells were annotated by placing a selection cube on the cell (**d**), fitting the cube to the size of the cell (**e**) and filling (**f**). Scale bar, 10 µm. **g**,**h**, Zoomed-in view of raw data (same volume as in **b**) (**g**) and annotation overlay generated in VR (**h**). Scale bar, 10 µm. **i**, Time spent for annotating a test patch using 2D-slice (*n* = 7) and VR annotation (*n* = 12 with *n* = 6 annotations performed with Arivis VisonVR and *n* = 6 annotations performed with syGlass). Data are presented as mean ± s.e.m. ***P = 0.0005, two-sided Mann–Whitney *U*-test. **j**, Instance Dice of 2D-slice annotation (*n* = 7) versus VR annotation (*n* = 12 with *n* = 6 annotations performed with Arivis VisonVR and *n* = 6 annotations performed with syGlass). Data are presented as mean ± s.e.m. *P = 0.0445, two-sided unpaired *t*-test. A.U., arbitrary units. Source data

Here, we developed DELiVR (deep learning and virtual reality mesoscale annotation pipeline), a virtual reality (VR)-aided deep-learning pipeline for detecting c-Fos^+^ cells in cleared mouse brains (Fig. 1a) that can be extended to other cell types. We generated high-quality annotations of light-sheet microscopy data of cleared whole mouse brains stained for c-Fos in a VR environment. Next, we trained a deep neural network on these data to identify c-Fos^+^ cells across the brain and mapped them automatically to the Allen Brain Atlas. To increase the usability of DELiVR, we packaged it into a single Docker container that runs via a plugin for the open-source software Fiji. DELiVR can also be trained with custom data via Fiji to adapt DELiVR to specific datasets. We used DELiVR to study cancer-related cachexia and found increased neuronal activity in mice with weight-stable cancer in brain areas related to sensory processing and foraging. In contrast, this increase was lost in cachectic animals, suggesting a weight-stable cancer-specific neurophysiological hyperactivation phenotype.

## Results

### Reference annotation is faster in VR compared to 2D slices

We used the SHANEL protocol^10^ for whole-brain c-Fos immunostaining, tissue clearing and light-sheet fluorescence microscopy (LSFM). To train deep-learning segmentation models in a supervised manner, substantial amounts of high-quality expert annotations are crucial. As common annotation approaches such as ITK-SNAP^11^ rely on time-consuming sequential two-dimensional (2D) slice-by-slice annotation, we used a VR approach that allows for full immersion into 3D volumetric data (Fig. 1b,c). We used two commercial VR annotation software packages (Arivis VisionVR and syGlass^12^) to evaluate the speed and accuracy of VR in comparison to 2D slice-based annotation in ITK-SNAP.

**Extended Data Fig. 1:**
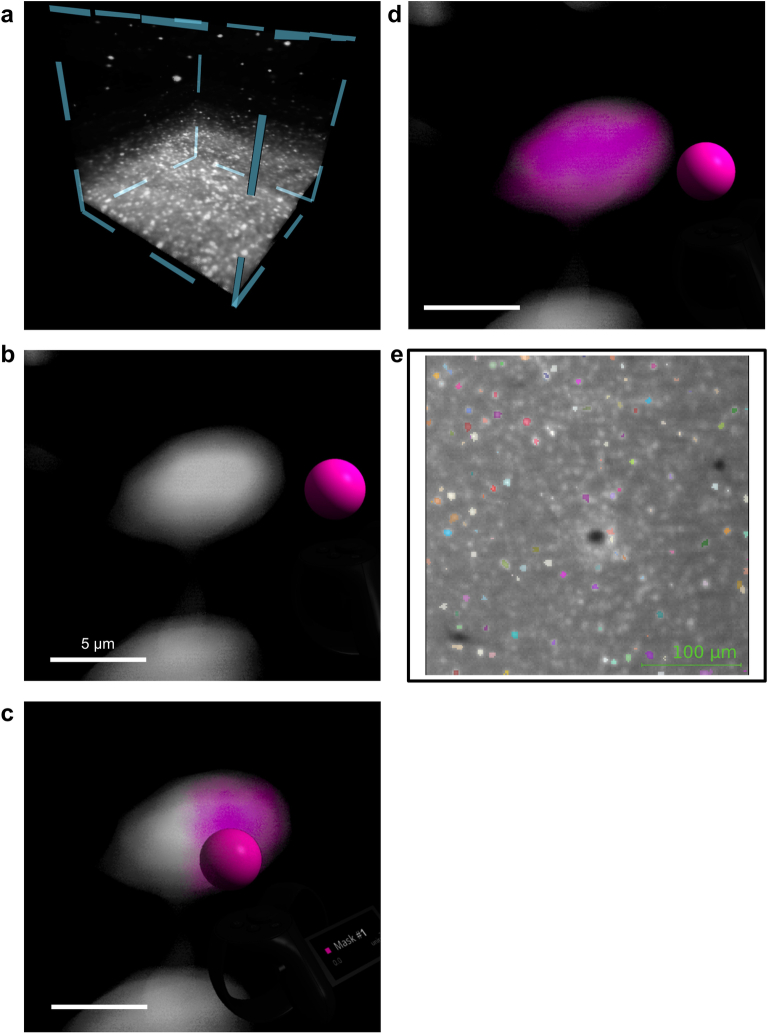
VR Segmentation in syGlass and 2D-slice-based segmentation using ITK-SNAP. a, Volume of raw data (c-Fos labeled brain) that was generated by light-sheet microscopy and loaded into syGlass. Volume size represents 200^3^ voxel, rendered isotropically. b-d, Using VR, individual cells were segmented in syGlass by using three-dimensional euclidean shapes as ROI and adjusting a threshold until the segmentation was acceptable. Scale bar indicates 5 µm. e, ITK-SNAP view of a single plane of the image stack. Cells were labeled in 2D, slice by slice. Segmentations are color coded by cell ID.

For annotation using Arivis VisionVR, the annotator defined a region of interest (ROI) in which an adaptive thresholding function was applied, according to the annotator’s input (Fig. 1d–h and Supplementary Video 1). In syGlass, the annotation tool allowed the annotator to draw simple 3D shapes as ROIs and adjust a threshold until the annotation was acceptable to the annotator (Extended Data Fig. 1a–d and Supplementary Video 2). In ITK-SNAP, individual c-Fos^+^ cells were segmented in each plane of the image stack (Extended Data Fig. 1e and Supplementary Video 3). We evaluated the time spent by the annotators for a 100³ voxel sub-volume (depicting 83 c-Fos^+^ cells) as well as the annotation quality of cell instances using the F1 score. We found that VR annotation was significantly (*P* = 0.0005, two-sided Mann–Whitney *U*-test) faster than 2D-slice annotation (Fig. 1i) and improved annotation quality (increase in F1 score from 0.7383 to 0.8032 (Fig. 1j)). Thus, we decided to generate reference data in VR for our deep-learning algorithm for c-Fos activity mapping.

### DELiVR outperforms threshold-based c-Fos segmentation

**Fig. 2:**
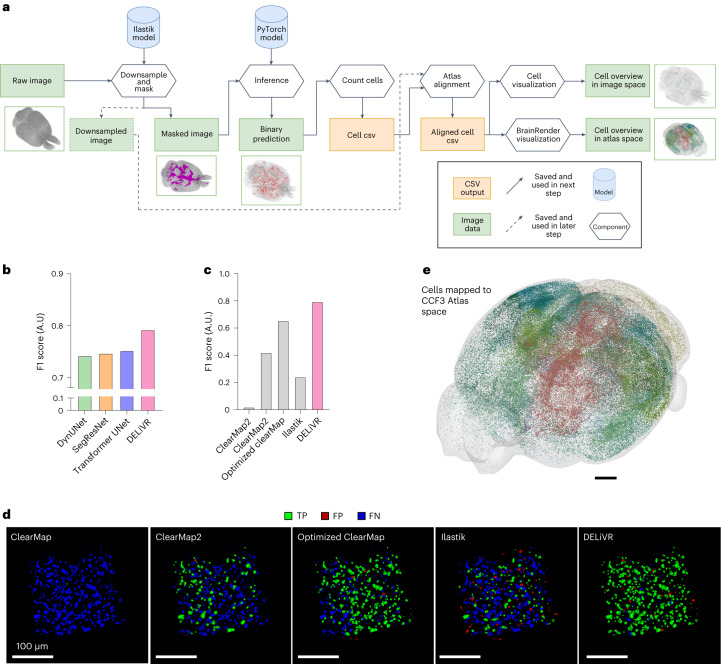
DELiVR’s UNet outperforms current methods for c-Fos^+^ cell detection. **a**, Scheme of the DELiVR inference pipeline. All components are packaged in a single Docker container. Raw image stacks serve as input. They are downsampled for atlas alignment and optionally masked (to exclude detection on ventricles). The masked images are then passed on to deep-learning cell detection (inference), which produces binary segmentations. The binarized cell’s center points are subsequently transformed to the Allen Brain Atlas CCF3 space. The cells are visualized in atlas space as (group-wise) heat maps and in image space as color-coded tiff stacks. **b**, Quantitative comparison of segmentation performance based on instance Dice (F1 score) between different deep-learning architectures and DELiVR. **c**, F1 scores for non-deep-learning methods (gray) and DELiVR (the same F1 score for DELiVR is used as in **b**). **d**, 3D qualitative comparison between ClearMap, ClearMap2, ‘Optimized’ ClearMap, Ilastik and DELiVR on instance basis. Predicted cells with overlap in reference annotations (TP) are masked in green, predicted cells with no overlap in reference annotations (FP) are masked in red. Undetected reference annotation cells (FN) are marked in blue. TP, true positive; FP, false positive; FN, false negative. Scale bar, 100 µm. **e**, Whole-brain segmentation output of the detected cells is visualized in atlas space using BrainRender. Scale bar, 1 mm in CCF3 atlas space. Source data

**Extended Data Fig. 2:**
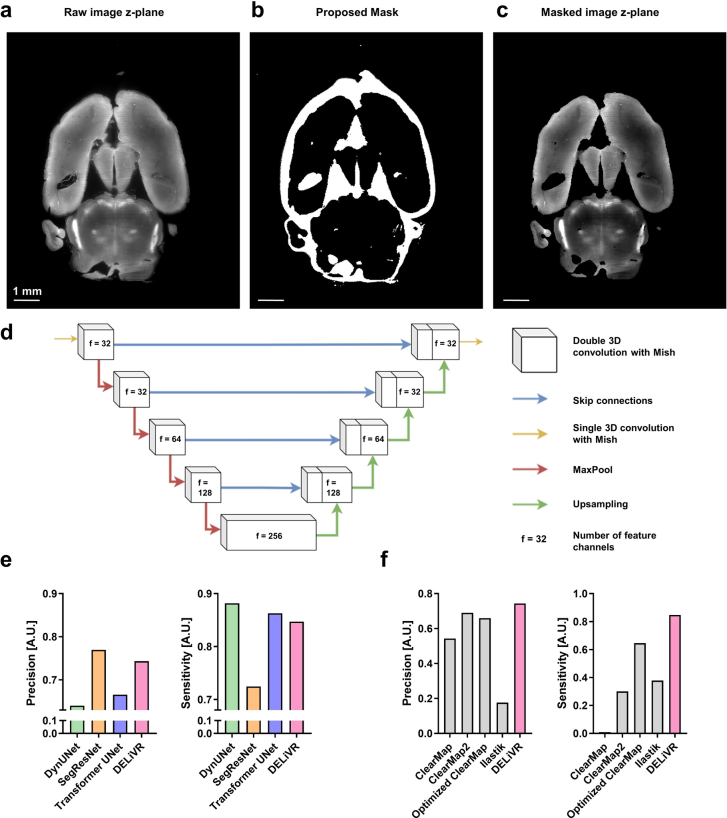
DELiVR pre-processing automatically removes artefacts. a-c, Horizontal view of an original image slice (a), the proposed mask (b) and the masked image slice generated (c). Scale bar = 1 mm. d, Architecture of the c-Fos deep-learning network; a MONAI 3D BasicUNet. e, Quantitative comparison (instance precision and sensitivity) of segmentation performance between deep-learning architectures and DELiVR’s 3D BasicUNet. f, Segmentation performance of non-deep-learning methods and DELiVR (Scores for DELiVR are the same as used in e). Source data

To comprehensively analyze neuronal activity across the entire brain, DELiVR detects and aligns the cells to the Allen Brain Atlas. DELiVR then visualizes the segmentation in both image and atlas space. Therefore, DELiVR consists of multiple steps (Fig. 2a). First, the pipeline downsamples the raw image stack and generates ventricle masks (Extended Data Fig. 2a–c). It then upscales the masks and uses them to mask the ventricles in the raw image input. DELiVR then utilizes a customized sliding-window inferer to identify potential cells. Afterwards, we conduct a connected component analysis^13^ to identify individual cells in the masked images and filter by size. DELiVR then aligns the previously downsampled brain to the Allen Brain Atlas (CCF3, 50 µm per voxel) with mBrainAligner^14^ and assigns the corresponding atlas region to each detected cell. The connected component analysis returns a set of center-point coordinates and volume for each segmented cell, which DELiVR then automatically maps to the Allen Brain Atlas with mBrainAligner.

To train and validate our model, we randomly sampled and VR-annotated 48 × 100³ voxel patches (referring to 5,889 cells) from a c-Fos-labeled brain. From these we trained a 3D BasicUNet (Extended Data Fig. 2d). In addition, we trained recent larger segmentation models, such as transformers^15^, SegResNet^16^ and the MONAI DynUnet^17^ to determine which model was best suited for our data. Assessing the instance performance by calculating the overlap between individual cells, the 3D BasicUNet architecture showed the best performance (based on F1 score) (Fig. 2b and Extended Data Fig. 2e). Therefore, we chose the 3D BasicUNet for our DELiVR pipeline.

**Extended Data Fig. 3:**
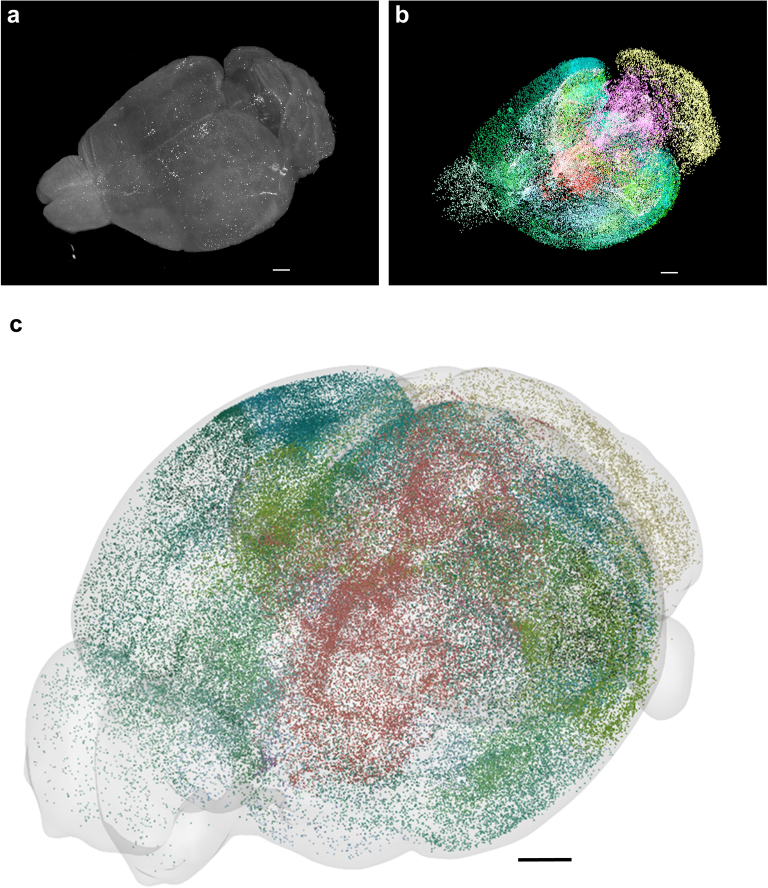
Whole-brain segmentation output generated with DELiVR. a, 3D visualization of a whole raw light-sheet image stack. b, 3D view of whole-brain segmentation output of detected cells by DELiVR. The area-wise color code from the Allen Brain Atlas was combined with the 3D segmentation. Thereby each cell is color coded according to the brain area it was detected in. The segmentation of cells is shown in the original image space. Scale bar = 500 µm. c, Visualization of the detected cells in CCF3 atlas space using BrainRender (same image as in Fig. 2e). Scale bar = 1 mm.

We also compared DELiVR with previously published non-deep-learning models that are applicable to cell detection in 3D images and had code available (ClearMap^7^, ClearMap2 (ref. ^18^) and Ilastik^19^). Our performance on the test set shows an F1 score of 0.7918 (+89.03% increase), instance sensitivity of 0.8470 (+181.64% increase), instance precision of 0.7434 (+7.74% increase) and a volumetric Dice of 0.6739 (+581,39% increase) compared to the second-best performing method, ClearMap2 (Fig. 2c,d, Extended Data Fig. 2f and Supplementary Table 1). We increased the performance of ClearMap based on the F1 score to 0.65 by manually pre-processing image stacks and optimizing parameters for cell detection^3^; however, DELiVR still had a superior performance. These scores demonstrate a clear improvement over filter and threshold-based segmentation methods as the deep-learning model captures 84.8 times more cells (1,611 true positives) than ClearMap (19 true positives), 2.8 times more cells than ClearMap2 (572 true positives) and 31.2% more than the optimized version of ClearMap (1,228 true positives) while not over-segmenting. For visualization, DELiVR generates a whole-brain segmentation output that exists in the original image space. Here, each segmented cell corresponds to a threshold value fitting to an Area ID of the Allen Brain Atlas and was colored according to the brain region that it belongs to (Extended Data Fig. 3a,b and Supplementary Video 4). In addition, we used BrainRender^20^ to plot and visualize the detected cells in the atlas space (Fig. 2e and Extended Data Fig. 3c).

**Fig. 3:**
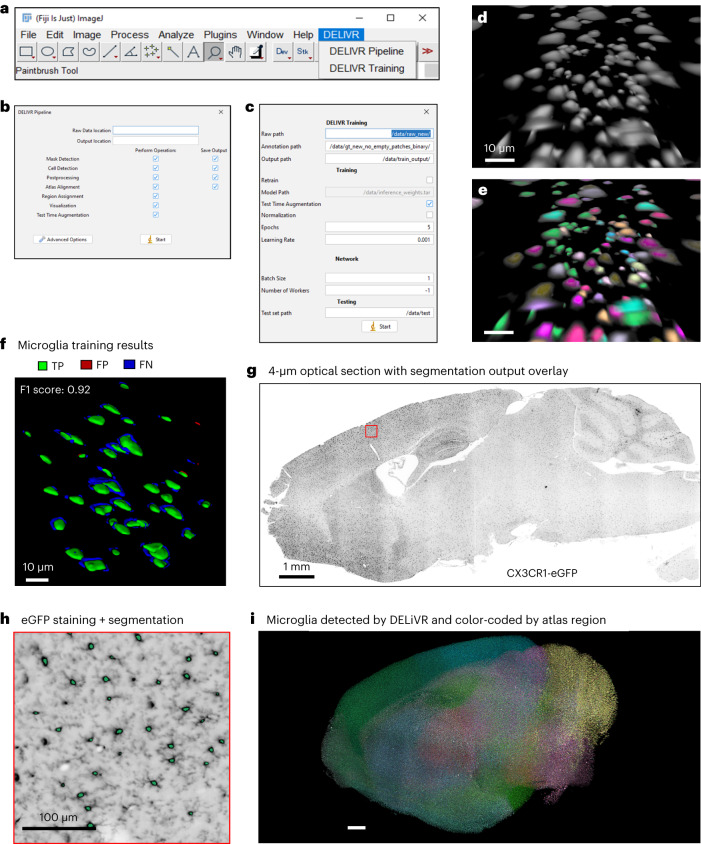
DELiVR runs end to end and can be adapted to other cell types. **a**–**c**, The DELiVR plugin will appear in Fiji upon installation. It can launch DELiVR for inference (**b**) or launch the training Docker to train on domain-specific training data (**c**). **d**,**e**, Zoomed-in Arivis VisionVR view of raw data from a CX3CR1^GFP/+^ microglia reporter mouse (**d**) and annotation overlay of cell bodies generated in VR (**e**). Scale bar, 10 µm. **f**, 3D representation of the training evaluation on instance basis; predicted cells with overlap in reference annotations are masked in green (TP), predicted cells with no overlap in reference annotations are masked in red (FP) and reference annotation cells with no corresponding prediction are marked in blue (FN). Following training, DELiVR segments microglia cell bodies with a Dice (F1) score of 0.92. Scale bar, 10 µm. **g**, Optical section of a CX3CR1^GFP/+^ microglia reporter mouse brain hemisphere (*n* = 1, sagittal), scanned at ×12 magnification and with inversed brightness (microglia indicates black spots). Scale bar, 1 mm. **h**, Zoomed-in view of the cortex (red inset in **g**), with overlaid segmented cells detected by whole-hemisphere DELiVR analysis shown in green (*n* = 1). Scale bar, 100 µm. **i**, Visualization of 12.2 million CX3CR1^GFP/+^ microglia across one hemisphere, generated by DELiVR and visualized with Imaris. Color-coding per Allen Brain Atlas CCF3 regions. Scale bar, 1 mm.

To increase usability, the entire DELiVR pipeline, encompassing atlas alignment, cell detection and visualization, is available as a single, user-friendly Docker container for both Linux and Windows. Docker is a software platform that allows to bundle and distribute applications, along with their required components, in a uniform container format^21^. We also developed a dedicated Fiji plugin to seamlessly run the DELiVR Docker (Fig. 3a–c and Supplementary Video  5).

Moreover, we provide a Docker container for training that integrates with the DELiVR Fiji plugin (Fig. 3a,c). This feature allows users to (re-)train DELiVR on other datasets, thereby enhancing DELiVR’s precision and adaptability. Users can choose to fine-tune the existing c-Fos model or train their own model from scratch. For this, one can adjust hyperparameters such as the number of epochs and learning rate. The user-trained model can then be used in the DELiVR pipeline as the inference model. For a comprehensive guide, please consult our ‘DELiVR handbook’ (Supplementary Note).

We used our DELiVR training to annotate microglial cell bodies, the brain’s resident macrophages^22^. We performed whole-brain nanobody labeling in CX3CR1^GFP/+^ reporter mice, clearing and LSFM, and annotated microglia somata in VR (Fig. 3d,e). Training used a dataset of 161 VR-annotated 100^3^ voxel patches with a total of 3,798 annotated microglia somata. The newly trained model had an F1 score of 0.92, indicating robust performance^23^ (Fig. 3f). We applied this model in the DELiVR pipeline and could detect and map microglia cell bodies throughout the brain. Using DELiVR’s visualization tool, we evaluated the microglia cell body segmentation output generated by DELiVR in our original images (Fig. 3g–i) and mapped the segmented cells to region IDs of the Allen Brain Atlas (Fig. 3i). Thereby, DELiVR allows to find and confirm an anatomical or functional sub-area in the original image stack of the brain.

### DELiVR identifies activation patterns in tumor-bearing mice

Cancer affects normal physiology locally in the surrounding tissue but can also lead to profound changes in the systemic metabolism of the patient. This is exemplified by the wasting syndrome cancer-associated cachexia (CAC) characterized by involuntary loss of body weight^24–26^ and specific changes in brain activity^27^.

**Fig. 4:**
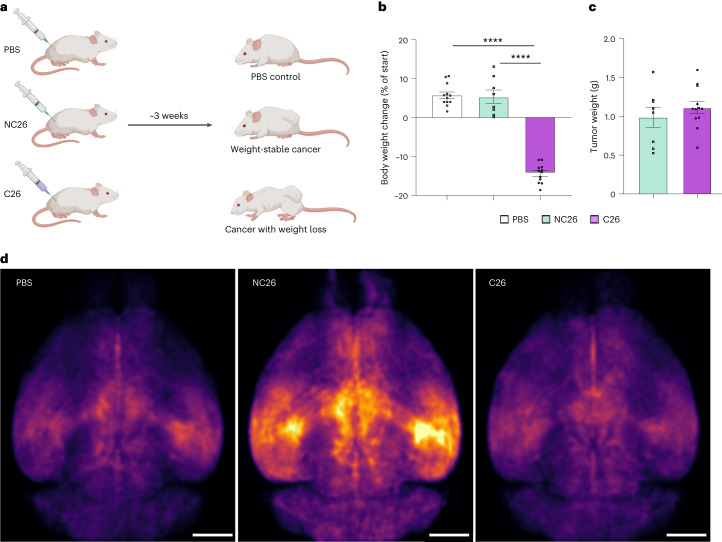
DELiVR identifies changes in neuronal activity in weight-stable cancer. **a**, Experimental setup. Adult mice were subcutaneously injected with PBS as control; NC26 cells that lead to a weight-stable cancer or cachexia-inducing C26 cancer cells. **b**, Body weight change of mice at the end of the experiment compared to starting body weight. Tumor weight was subtracted from the final body weight. *n*(PBS) = 12, *n*(NC26) = 8, *n*(C26) = 12. Data are presented as mean ± s.e.m. ****P < 0.0001, one-way ANOVA with Sidak post hoc analysis **c**, Tumor weight at the end of the experiment. *n*(NC26) = 8, *n*(C26) = 12. Data are presented as mean ± s.e.m. **d**, Normalized c-Fos^+^ cell density in brains of PBS controls, mice with weight-stable cancer (NC26) and mice with cancer-associated weight loss (C26), visualized in CCF3 atlas space. *n*(PBS) = 12, *n*(NC26) = 8, *n*(C26) = 12. Scale bars, 2 mm. Source data

**Extended Data Fig. 4:**
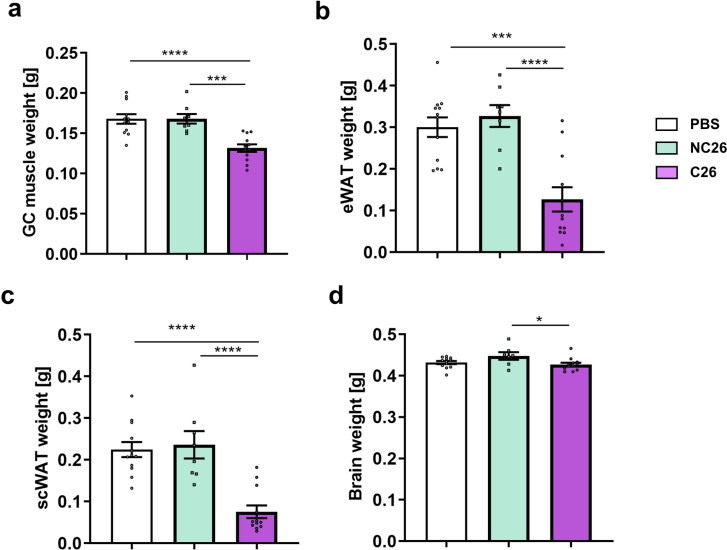
Tissue weights of mice with weight-stable cancer (NC26) and cancer-associated weight loss (C26). a, Gastrocnemius (GC) muscle weight. n(PBS) = 12, n(NC26) = 8, n(C26) = 12, ****p < 0.0001,***p = 0.0004, One-way ANOVA with Sidak post hoc analysis. b, Epididymal white adipose tissue (eWAT) weight. n(PBS) = 12, n(NC26) = 8, n(C26) = 12, **p(PBS vs C26) = 0.0040,**p(NC26 vs C26) = 0.0015, Kruskal-Wallis test with Dunn´s multiple comparison test. c, Subcutaneous WAT (scWAT) weight. ***p = 0.0003, **p = 0.0019, Kruskal-Wallis test with Dunn´s multiple comparison test. d, Brain weight. n(PBS) = 12, n(NC26) = 7, n(C26) = 12, *p = 0.0479, One-way ANOVA with Sidak post hoc analysis. All data are presented as mean values +/− SEM. Source data

To identify brain regions affecting body weight maintenance in cancer, we used DELiVR to compare the neuronal activity patterns between weight-stable cancer and CAC. We subcutaneously transplanted NC26 colon cancer cells that give rise to weight-stable cancer or C26 colon cancer cells that induce weight loss (Fig. 4a). As expected^28^, no changes in body weight were observed in NC26 tumor-bearing mice compared to controls, whereas C26 tumor-bearing mice showed significant (*P* < 0.0001, one-way analysis of variance (ANOVA) with Sidak post hoc analysis) reductions (Fig. 4b). The differences in body weight were not due to differences in tumor mass (Fig. 4c). C26 tumor-bearing mice displayed reduced weights of the gastrocnemius muscle and white adipose tissue depots (Extended Data Fig. 4a–c). We observed a small but statistically significant (*P* = 0.0479, one-way ANOVA with Sidak post hoc analysis) decrease in brain weights of cachectic C26 versus weight-stable NC26 tumor-bearing mice (Extended Data Fig. 4d). We performed c-Fos antibody labeling, clearing and imaging of whole brains of these mice and applied DELiVR for whole-brain mapping of neuronal activity. c-Fos^+^ density maps indicated an increase in brain activity in weight-stable NC26 tumor-bearing mice compared to phosphate-buffered saline (PBS) controls, whereas this increase was not present in cachectic C26 tumor-bearing mice (Fig. 4d).

**Fig. 5:**
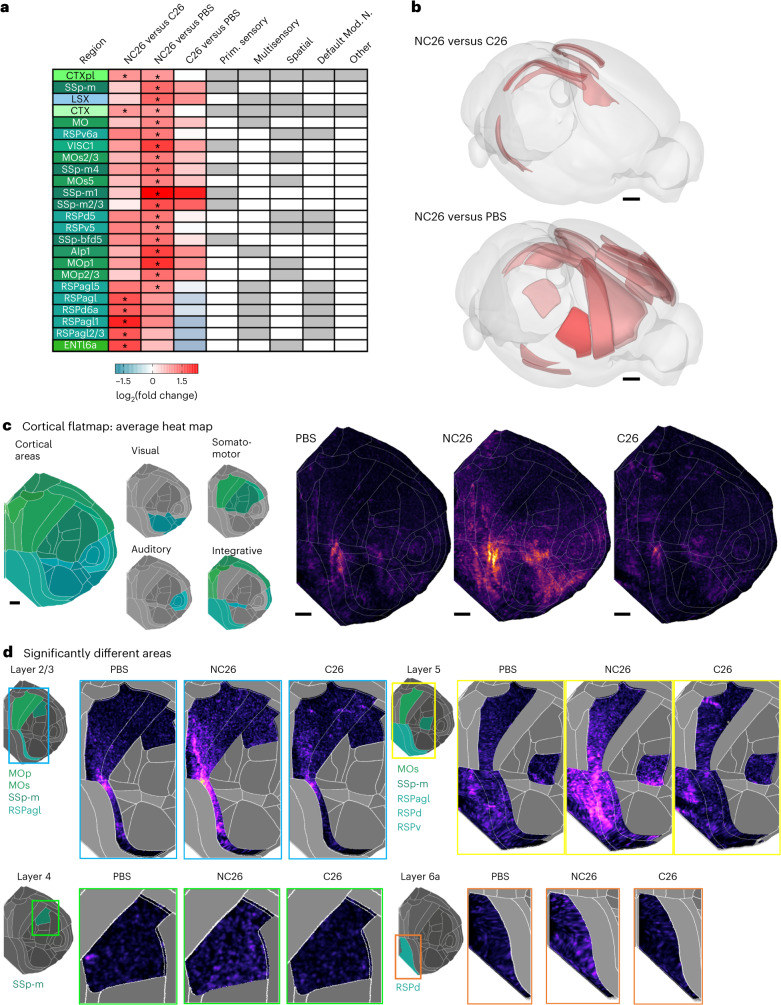
DELiVR identifies cancer-related brain activation patterns. **a**, Brain-region-wise c-Fos^+^ cell density log_2_(fold change) compared between the three groups. *Padj < 0.1 (two-sided unpaired *t*-tests with Benjamini–Hochberg multiple-testing correction with FWER = 0.1, *n*(PBS) = 12, *n*(NC26) = 8, *n*(C26) = 12). **b**, Brain areas with significantly different (*P*adj < 0.1) c-Fos expression between NC26/C26 (top) or NC26/PBS (bottom) visualized using BrainRender. Red indicates significantly (*Padj < 0.1) more c-Fos^+^ cells in NC26 in both cases. Two-sided unpaired *t*-tests with Benjamini–Hochberg multiple-testing correction with FWER = 0.1, *n*(PBS) = 12, *n*(NC26) = 8, *n*(C26) = 12. Scale bars, 1 mm. **c**, Flattened-cortex visualizations of normalized c-Fos^+^ cell density for PBS control mice (*n* = 12), NC26 (*n* = 8) and C26 tumor-bearing mice (*n* = 12), Scale bars, 1 mm in flattened-cortex projection space (flattened from CCF3 atlas space). **d**, c-Fos^+^ cell density in cortical subregions that were statistically significantly (*Padj < 0.1) different after multiple-testing correction. Two-sided unpaired *t*-tests with Benjamini–Hochberg multiple-testing correction with FWER = 0.1, *n*(PBS) = 12, *n*(NC26) = 8, *n*(C26) = 12. Source data

The increase in brain activity in NC26 tumor-bearing mice was most pronounced throughout the cortical plate and in the lateral septal complex (Fig. 5a,b). Overall, we identified 19 areas in NC26 tumor-bearing mice that showed statistically significantly (*P*adj < 0.1, two-sided unpaired *t*-tests with Benjamini–Hochberg multiple-testing correction with family-wise error rate (FWER) = 0.1) increased c-Fos expression compared to PBS controls after multiple-testing correction (Fig. 5a). We found that NC26-bearing mice also have more c-Fos^+^ cells in the cortical plate, with the most pronounced differences observed in the somatomotor areas (Fig. 5a,b). NC26 tumors notably increased c-Fos^+^ density in the somatosensory cortex related to the snout, specifically in the mouth region (layer 2/3 and 4) and barrel field layer 4. Furthermore, NC26-bearing mice showed more c-Fos^+^ cells than PBS controls in the primary (layers 1 and 5) and secondary motor areas (layers 2/3 and 5; Fig. 5a,b). The primary motor cortex layer 5 is especially interesting because it contains extratelencephalic projection neurons that project as far as the spinal cord, among others^29^.

We found seven areas that were significantly altered between NC26 and cachectic C26 tumor-bearing mice, whereas we did not observe significant changes (*P*adj > 0.1, two-sided unpaired *t*-tests with Benjamini–Hochberg multiple-testing correction with FWER = 0.1) in c-Fos^+^ expression when comparing PBS and cachectic C26 tumor-bearing mice after correcting for multiple testing (Fig. 5a). When comparing NC26 to C26 tumor-bearing mice, we found that NC26 mice had more c-Fos^+^ cells overall in the cortical plate, with the differences clustering in the dorsal and agranular lateral retrosplenial cortex as well as a subset of entorhinal cortex (Fig. 5a,c). Evaluation of c-Fos^+^ density heat maps offer additional details (Fig. 5d). Evaluation of layer 2/3 retrosplenial cortex shows that activity clusters in the anterior third of retrosplenial cortex in PBS and C26, but not NC26 retrosplenial cortex, where it is both stronger and spread further to the back. The retrosplenial cortex is thought to be a site of multisensory integration, spatial integration and environment mapping^30^ and is crucially involved in foraging behavior^31^.

Overall, our findings showed that brain activity in weight-stable NC26 cancer-bearing mice are markedly different from both cachectic C26 cancer-bearing mice and PBS controls. Specifically, we find a consistent hyperactivation phenotype in NC26 brains that is detectable at the whole-cortex level, but most pronounced in areas relevant to somatosensation at the snout and motor planning, as well as spatial navigation (all of which would be consistent with a foraging-related brain activation pattern). Thus, with DELiVR we were able to identify a neuronal activity pattern specific to the NC26 cancer model.

## Discussion

Here, we present DELiVR, an end-to-end VR-enabled deep-learning-based quantification pipeline for whole-brain cell mapping in cleared mouse brains. We designed it to make deep learning accessible to most biologists via a Fiji front end, not requiring coding skills. We leveraged VR technology to generate reference annotations for training a deep-learning-based segmentation network. DELiVR improves segmentation accuracy compared to current cell detection methods and generates a registered segmentation output that can be examined in the original image and in the atlas spaces. In addition, our Fiji training feature enables users to adapt DELiVR to their specific datasets, increasing its versatility and usability.

Traditional, non-machine-learning solutions for large-scale analysis of c-Fos cell detection, such as ClearMap^7,18^ rely on a sophisticated system of thresholding and filtering to detect small structures and classify them as cells. While such approaches generated valuable information^6^, their performance is limited for data with variable signal-to-noise ratios, as is the case when imaging large volumes such as the entire mouse brain. Though parameters can be adjusted, it is difficult to find a setting that accounts for all cells. Hence, the thresholds tend to be set conservatively, meaning that subtle differences may be lost during threshold-based analysis. A trained deep-learning model learns these local variances, thus providing more accurate cell number estimates than threshold-based methods, as exemplified by DELiVR’s high instance F1 score. Previous approaches for segmenting cells in mouse brains ranged from deep learning^32–34^, random forest algorithms^19,35^ and threshold-based solutions^7,18,36^; however, only a subset of studies published their analysis pipeline and model weights in a working package that makes it applicable for other datasets. We found that DELiVR’s 3D BasicUNet consistently outperformed all other approaches with available code. In addition to providing highly accurate AI-based cell detection, DELiVR provides a unique and accessible open-source tool, functioning seamlessly within Fiji. It encompasses all steps of brain activity mapping, including cell detection, atlas alignment and visualization, in an easily accessible environment without the need for writing additional computer code.

Our experiments showed that VR is a superior means of annotation and data exploration for volumetric data analysis. Non-VR methods show orthogonal slices, which allows an annotator to outline the shape of individual cells in 2D; however, it obfuscates necessary volumetric information, making annotation challenging and time consuming; an annotator never sees the whole cell, only a cross-section and must scroll through slices to ensure that it is in fact a cell and not background noise. In contrast, VR allows the annotator to capture 3D structures in their entirety, enabling the fast generation of more reliable annotated data.

In future work, it will be interesting to explore the possibility of performing active learning in a VR environment. Active learning is a combined machine-learning training and annotation approach, where a model selectively chooses the most informative or uncertain data points for manual annotation, allowing for efficient model improvement with fewer labeled data points^37^. This approach is currently limited by the possibilities of the VR annotation software application programming interfaces. Using an ensemble of networks or a test time augmentation uncertainty map as well as methods such as Monte-Carlo-based sampling using dropout layers^38^ to highlight areas that are ambiguous to the network, the annotator can be guided to even more efficient time use in VR annotation. The annotators’ choices can then be fed into a fine-tuning step to improve the model while annotating.

We used DELiVR to profile the brain activation patterns of cancer-bearing mice that were either weight-stable or displayed CAC. A mix of reduced food intake, elevated catabolism, increased energy expenditure and inflammation drives weight loss in cancer^26^. The brain was shown to contribute to anorexia in CAC, as it responds to inflammatory cytokines that modulate the activity of neuronal populations that regulate appetite^39^. In addition, activation of neurons in the parabrachial nucleus was shown to suppress appetite in mouse models of CAC^40^. The reduction in brain weight among cachectic C26 tumor-bearing mice aligns with prior reports of decreased brain weight in mice with cachexia-inducing pancreatic tumors^41^. It is currently unclear whether this this volume reduction is due to cell death, or white matter loss.

Notably, we found a substantial increase in c-Fos^+^ expression in the brains of weight-stable NC26 tumor-bearing mice, especially in motor and sensory areas, and higher-order regions such as the retrosplenial cortex. Those regions are linked to sensorimotor control, motor sequencing and foraging^30,42,43^. The abundance of sensory-related regions suggests cancer-specific impairment in GABAergic inhibition^44^, driving a hyperactivation phenotype via disinhibition. If and how these increases in neuronal activation in weight-stable cancer-bearing mice affect body weight maintenance will be of a high interest to explore in future studies.

In conclusion, we present DELiVR: an integrated, easy-to-use pipeline to label, scan and analyze neuronal activity markers across the entire mouse brain and show how VR increases the speed and accuracy of generating reference annotations. Using DELiVR, we find differences in c-Fos expression between cachectic and non-cachectic cancer mouse brains, pointing us to a previously unknown neurophysiological phenotype in cancer-related weight control.

## Methods

### Whole-brain immunolabeling and clearing

Immunostaining for c-Fos was performed using a modified version of SHANEL^10^. All incubation steps were carried out under moderate shaking (300 rpm). For the pretreatment, samples were dehydrated with an ethanol/water series (50%, 70% and 100% ethanol) at room temperature for 3 h per step. Next, samples were incubated in dichloromethane (DCM)/methanol (2:1 v/v) at room temperature for 1 day. Brains were rehydrated with an ethanol/water series (100%, 70% and 50% ethanol and diH_2_O) at room temperature for 3 h per step. Samples were incubated in 0.5 M acetic acid at room temperature for 5 h followed by washing with diH_2_O. Next, brains were incubated in 4 M guanidine HCl, 0.05 M sodium acetate, 2% v/v Triton X-100, pH 6.0, at room temperature for 5 h followed by washing with diH_2_O. Brains were incubated in a mix of 10% CHAPS and 25% *N*-methyldiethanolamine at 37 °C for 12 h before washing with diH_2_O. Blocking was performed by incubating the brains in 0.2% Triton X-100, 10% dimethylsulfoxide and 10% goat serum in PBS shaking at 37 °C for 2 days. Samples were incubated with c-Fos primary antibody (Cell Signaling Technology, 2250, 1:1,000 dilution) in primary antibody buffer (0.2% Tween-20, 5% dimethylsulfoxide, 3% goat serum and 100 µl heparin per 100 ml PBS) shaking at 37 °C for 7 days. The antibody solution was filtered (22-µm pore size) before use. Samples were washed in washing solution (0.2% Tween-20 and 100 µl heparin in 100 ml PBS) shaking at 37 °C for 1 day at which the washing solution was refreshed five times. Brains were incubated with the secondary antibody (Alexa Fluor 647 and goat anti-rabbit IgG (H + L) from Invitrogen, A-21245, 1:500 dilution) in secondary antibody buffer (0.2% Tween-20, 3% goat serum and 100 µl heparin per 100 ml PBS) shaking at 37 °C for 7 days followed by incubating in washing solution shaking at 37 °C for 1 day at which the washing solution was refreshed five times. Brains were dehydrated using 3DISCO^2^ with a THF/H_2_O series (50%, 70%, 90% and 100% THF) for 12 h per step followed by an incubation in DCM for 1 h. Tissues were incubated in benzyl alcohol/benzyl benzoate (1:2 v/v) until tissue transparency was reached (>4 h).

For microglia labeling, brains of CX3CR1^GFP/+^ mice were pretreated via the modified SHANEL protocol as described above and incubated with Atto647N-conjugated anti-GFP nanobooster (Chromotek, gba647n-100, 1:1,000 dilution) with 5% 2-hydroxypropyl-β-cyclodextrin, 0.2% Tween-20 and 6% goat serum in PBS for 5 days at 37 °C. Brains were washed as described in washing solution shaking at 37 °C for 1 day at which the washing solution was refreshed five times. Brains were dehydrated with an ethanol/dH_2_O series (50%, 70%, 90% and 100% ethanol) at room temperature for 2 h each step and incubated in 100% ethanol overnight. Subsequently, brains were incubated in DCM for 1 h before incubation in benzyl alcohol/benzyl benzoate until tissue transparency was reached.

### Light-sheet imaging

Light-sheet imaging for c-Fos labeled brains was conducted through a ×4 objective lens (Olympus XLFLUOR 340) equipped with an immersion-corrected dipping cap mounted on an UltraMicroscope II (LaVision BioTec) coupled to a white light laser module (NKT SuperK Extreme EXW-12). The antibody signal was visualized using a 640/40 nm excitation and 690/50 nm emission filter. Tiling scans (3 × 3 tiles) were acquired with a 15–20% overlap, 60% sheet width and 0.027 NA. The images were taken in 16-bit depth and at a nominal resolution of 1.625 μm per voxel on the *xy* axes. In the *z* dimension we took images in 6-μm steps using left- and right-sided illumination. Whole-brain scans for microglia-labeled CX3CR1^GFP/+^ brains were generated with the LaVision BioTec Ultramicroscope Blaze coupled with LaVision BioTec MI PLAN ×12 objective (0.53 NA (WD = 10 mm), nominal pixel size of 0.54 µm in *xy*). Stitching of tile scans was carried out using Fiji’s stitching plugin, using the ‘Stitch Sequence of Grids of Images’ plugin^45^ and custom Python scripts.

### ClearMap

ClearMap^7^ and the CellMap portion^18^ of ClearMap2 were used with adapted settings for thresholds and cell sizes that fitted to the higher resolution and different signal-to-noise ratios in our dataset. Segmentation masks were saved as tiff stacks by toggling the ‘save’ option in the last segmentation step. ClearMap was ported to Python (v.3.5) before use, but functioned identically^46^. We only used the cell segmentation portions, no pre-processing (for example ClearMap2’s flat-field correction) or post-processing, such as atlas alignment, were performed. Both pipelines were run for an entire brain and subsequently subdivided into test patches that we used for the comparisons with DELiVR. For ‘optimized ClearMap’^3^, we performed the following pre-processing steps on our image stack: (1) Background equalization to homogenize intensity distribution and appearance of the c-Fos^+^ cells over different regions of the brain, using pseudo-flat-field correction function from Bio-Voxxel toolbox (10.5281/zenodo.5986129). (2) Convoluted background removal, to remove all particles bigger than relevant cells. This was performed with the median option in the Bio-Voxxel toolbox. (3) A 2D median filter to remove remaining noise after background removal. (4) Unsharpen mask to amplify the high-frequency components of a signal and increase overall accuracy of the cell detection algorithm of ClearMap. (5) A *z*-wise removal of artifacts by manually selecting ROIs in Fiji. After pre-processing, ClearMap^7^ was applied by following the original publication and considering the threshold levels that we obtained from the pre-processing steps.

### Ventricle masking

We wrote an automated pre-processing script that downsamples the image stack to an isotropic 25 × 25 × 25 µm per voxel and then applies a custom-trained random forest to identify ventricles. Specifically, we integrated Ilastik^19^ (v.1.4.0b8) with a 3D pixel classifier, which we trained on several downsampled brain image stacks to differentiate between ventricles and brain parenchyma. The pre-processing script then generates a 3D mask stack that our script upsamples to the original image stack dimensions, using bicubic interpolation to avoid aliasing artifacts at ventricle edges. It then masks each original *z*-plane image with the respective mask, pads it and returns a 16-bit image stack (saved as one big .npy file that can be read via np.memmap).

### Annotation

VR annotation for c-Fos^+^ cells was carried out using Arivis VisionVR (v.3.4.0, Carl Zeiss Microscopy Software Center Rostock) or syGlass (v.1.7.2, ref. ^12^). For this purpose, the annotator was wearing a VR headset (Oculus Rift S) and carried out annotations in VR using hand controllers (Oculus Touch). Slice-by-slice annotation was carried out using ITK-SNAP (v.3.8, ref. ^11^). For comparing VR and 2D-sliced based annotation, a 100^3^-voxel volume of c-Fos labeled brain was annotated by the participants and the time was recorded until the annotation task was finished. For training and testing our deep-learning network, we annotated a total of 48 × 100³ voxel patches in VR. All of our training and test patches were furthermore vetted by an expert biologist in ITK-SNAP to ensure that only cells were annotated. We evaluated the annotation quality using the formula of Dice as described below. For more details about the annotation process in VR, please see our ‘DELiVR handbook’ provided as a Supplementary Note. Microglia cell bodies were annotated in VR similar to c-Fos^+^ cells using Arivis VisionVR. Only the somata were annotated, while the microglia processes were excluded.

### Deep learning

To automatically segment the cells in all brains, we trained a 3D BasicUNet^47^ for DELiVR from the MONAI library^48^. The annotated dataset of 48 × 100³ patches was split into nine patches for testing and 39 patches for training stratified by signal after manual ventricle masking. As an activation function, we chose Mish^49^ and as optimizer Ranger21 (ref. ^50^). As a loss function, we used binary cross-entropy loss^17^. For the training of 500 epochs, we set the initial learning rate to 1 × 10^−3^ and the batch size to four. The network was then trained on a single GPU (NVIDIA RTX8000). Instead of conducting model selection, we selected the last checkpoint after 500 epochs of training. To compare the DELiVR 3D BasicUNet with other segmentation models, we trained UNETR^15^, SegResNet^16^and MONAI DynUNET^17^ with similar specifications.

The microglia 3D BasicUNet model was trained in a similar fashion for 500 epochs using 161 patches containing 3,798 cells. These were split into 129 patches for training and 32 patches for testing. Training was performed on an NVIDIA A100 GPU.

### Evaluation of the segmentation model

Evaluation of the deep-learning model was conducted in a twofold manner. First, we evaluated the volumetric segmentation quality by assessing, for each voxel, whether it was correctly classified as foreground or background using pymia^51^. A volumetric quality assessment gave us TPs, FPs, FNs and true negatives by comparing every prediction voxel with the reference annotation voxel. Additionally, we conducted an instance-wise assessment of the segmentation quality. Therefore, we assess detection rates on a single-cell (instance) level^52^. To fairly evaluate every cell irrespective of the patch, we aggregated the counts across all patches and computed the instance metrics globally^53^.

Volumetric and instance scores were calculated according to the following equations:

$${\mathrm{Dice}}=\frac{2\mathrm{TP}}{2\mathrm{TP}+\mathrm{FP}+\mathrm{FN}}\qquad{\mathrm{Sensitvity}}=\frac{\mathrm{TP}}{\mathrm{TP}+\mathrm{FN}}\qquad{\mathrm{Precision}}=\frac{\mathrm{TP}}{\mathrm{TP}+\mathrm{FP}}$$

Comparison with ClearMap, ClearMap2, ‘Optimized ClearMap’ and Ilastik was performed on a test brain to generate segmentations from which we cropped 100³-voxel patches to avoid artifacts that occur when the methods are applied at the patch level. These patches were then compared to our reference annotation using the same metrics as described above.

### Atlas registration and statistical analysis

For atlas registration, we used mBrainAligner^14^, which worked well with our datasets (Supplementary Fig. 1). We manually saved the downsampled isotropic 25 × 25 × 25 µm per voxel stacks as .v3draw using Vaa3d^54^. Subsequently, we wrote an automated script that aligned the image stacks to mBrainAligner’s 50 × 50 × 50 µm per voxel version of the Allen Brain Atlas CCF3 reference atlas, using the LSFM example settings with minor adaptations. Subsequently, we used mBrainAligner’s swc transformation tool to map the center-point coordinates of our c-Fos^+^ cells into atlas space.

Furthermore, we wrote a custom cell-to-atlas script (reusing parser code from VeSSAP^55^ and the Allen Brain Atlas CCF3 atlas file as provided by the Scalable Brain Atlas^56^) that filters the cells by size, with a user-defined upper and lower limit and returns two tables: a table with each cell as a row, including the region and Allen Brain Atlas color code, etc. and a region table with one region per row, in which the number of c-Fos^+^ cells per region is summarized. For all datasets, the post-processing script generates overview tables that contain cell counts for all regions. We used the latter for uncorrected Student’s *t-*tests. Finally, we implemented a level-aware multiple-testing script that compares groups at the Allen Brain Atlas’s 11 structure levels. We excluded the fiber tracts from our statistical comparisons.

### Visualization

For visualizing the cells and regions in atlas space, we used BrainRender^20^ (v.2) with a modified density plot function^46^. To visualize the segmented cells in the original image space, we combined the area-wise color code from the Allen Brain Atlas with the 3D segment mask output by the connected component analysis. The result is a cell mask file with each cell being color coded according to the brain area that it belongs to, which makes overlaying with the original image data in for example Fiji easy and allows for direct visual inspection of the segmentation results. Finally, we used the Allen Institute for Brain Science’s cortical flat-map code (https://github.com/int-brain-lab/atlas) with adaptions^46^ to include our heat maps.

### DELiVR Docker and Fiji plugin

We packaged the DELiVR pipeline as provided in GitHub (https://github.com/erturklab/delivr_cfos) into a Docker container (base, nvidia/cuda:11.7.2-runtime-ubuntu22.04) including mBrainAligner^14^ (https://github.com/Vaa3D/vaa3d_tools/tree/master/hackathon/mBrainAligner), Ilastik (https://www.ilastik.org/download.html, v.1.4.0b8) and TeraStitcher portable^57^ (https://github.com/abria/TeraStitcher/wiki/Binary-packages#terastitcher-portable-no-gui-only-command-line-tools, v.1.11.10). The code included Python (v.3.8), PyTorch (v.1.11), PyTorch Lightning (v.2.0.5), Nibabel (v.5.1.0), MONAI (v.1.2.0), SciPy (v.1.8.1), NumPy (v.1.24.4), Pandas (v.1.4.3), imglib2 (https://github.com/imglib/imglib2) and cc3d (https://github.com/seung-lab/connected-components-3d). For details, please see the Docker file on GitHub (https://github.com/erturklab/delivr_cfos/blob/main/Dockerfile).

We wrote the Fiji^58^ (v.1.52p) plugin in Java (v.1.8, using Maven (v.3.9.5) and Jackson, https://github.com/FasterXML/jackson) as a front end. This provides a graphical user interface that compiles a config.json with path names and analysis parameters. Subsequently, the plugin calls the Docker container via a shell command and displays the progress of the pipeline. For a more detailed description, please see our ‘DELiVR handbook’ provided as a Supplementary Note.

### Docker for training and Fiji plugin

We packaged the training code (https://github.com/erturklab/delivr_train) as a separate Docker container, which is also accessible via the Fiji plugin. The training plugin accepts annotated patches and trains a model specifically for this dataset. This model can then be imported into the inference pipeline for dataset-specific inference for any cell type. The Fiji training plugin compiles a config_train.json and arranges the file layout for the training Docker. It displays the training progress and shows the final test scores at the end.

### Cell culture

C26 and NC26 colon cancer cells were cultured in high-glucose DMEM with pyruvate (Life Technologies, 41966052), supplemented with 10% fetal bovine serum (Sigma-Aldrich, F7524) and 1% penicillin-streptomycin (Thermo Fisher, 15140122) as described previously^28,59^. Before using the cells for transplantation, cells had a confluence of 80%. Cells were trypsinized, counted and required cell numbers were suspended in Dulbecco’s PBS (Thermo Fisher, 14190250).

### Animal experimentation

Experiments were carried out with male BALB/c mice aged 10–12 weeks. They were purchased from Charles River Laboratories, maintained on a 12-h light–dark cycle and fed a regular unrestricted chow diet. The set points in the animal room were set to 20–24 °C temperature and 45–65% humidity. The mice were injected with 1 × 10^6^ C26 or 1.5 × 10^6^ NC26 colon cancer cells^28,59^ in 50 µl PBS subcutaneously into the right flank. Control mice were injected with 50 µl PBS. After 5 days from cell implantation, mice were monitored daily for tumor growth and body weight. Cachectic C26 tumor-bearing mice were considered cachectic when they had lost 10–15% of body weight. Mice were killed following deep anesthesia with a mix of ketamine/xylazine, followed by intracardiac perfusion with heparinized PBS (10 U ml^−1^ heparin) and by a perfusion with 4% paraformaldehyde (PFA). Tissues and organs were dissected, weighed and post-fixed at 4 °C overnight. Animal experimentation was performed in accordance with European Union directives and the German Animal Welfare Act (Tierschutzgesetz) and approved by the state ethics committee and the Government of Upper Bavaria (ROB-55.2-2532.Vet_02-18-93).

The 6–8-week-old CX3CR1^GFP/+^ (B6.129P-Cx3cr1tm1Litt/J) mice were purchased from The Jackson Laboratory (strain code 005582). They were deeply anesthetized using a combination of midazolam, medetomidine and fentanyl, intracardially perfused with 15 ml 0.01 M PBS solution (10 U ml^−1^ heparin) and 15 ml 4% PFA solution. The brain was dissected, post-fixed in 4% PFA for 6 h, then proceeded for staining and clearing following the SHANEL protocol. CX3CR1^GFP/+^ mice were killed for organ withdrawal (Tötung zu Wissenschaftlichen Zwecken/Organentnahme) in accordance with the German law for animal experiments (Tierschutzgesetz), paragraph 4, section 3.

### Statistical analysis

Results from biological replicates were expressed as mean ± s.e.m. Statistical analysis was performed using GraphPad Prism (v.9). Normality was tested using Shapiro–Wilk normality tests. To compare two conditions, unpaired Student’s *t*-tests or Mann–Whitney *U*-tests were performed. A one-way ANOVA with Sidak’s post hoc test or Kruskal–Wallis tests with Dunn’s multiple comparison test were used to compare three groups. For the c-Fos^+^ density comparison between areas, we used two-sided *t*-tests followed by Benjamini–Hochberg multiple-testing correction with a false discovery rate (FWER) of 0.1, as implemented in SciPy statsmodels.stats.multitest.multipletests module (https://www.statsmodels.org/dev/generated/statsmodels.stats.multitest.multipletests.html).

### Reporting summary

Further information on research design is available in the Nature Portfolio Reporting Summary linked to this article.

## Online content

Any methods, additional references, Nature Portfolio reporting summaries, source data, extended data, supplementary information, acknowledgements, peer review information; details of author contributions and competing interests; and statements of data and code availability are available at 10.1038/s41592-024-02245-2.

### Supplementary information

Supplementary Information Supplementary Fig.1, Supplementary Table 1 and DELiVR handbook.

Reporting Summary

Supplementary Video 1 Annotation of cells in virtual reality using Arivis VisionVR.

Supplementary Video 2 Annotation of cells in VR using syGlass.

Supplementary Video 3 2D-slice-based annotation using ITK-snap.

Supplementary Video 4 Whole-brain with c-Fos^+^ cells detected by DELiVR depicted in the original image space. Cells are color coded by the area they belong to in the Allen Brain Atlas.

Supplementary Video 5 Example run of the ImageJ plugin on Ubuntu Linux.

### Source data

Source Data Fig. 1 Numerical source data.

Source Data Fig. 2 Numerical source data.

Source Data Fig. 4 Numerical source data.

Source Data Fig. 5 Numerical source data.

Source Data Extended Data Fig. 2 Numerical source data.

Source Data Extended Data Fig. 4 Numerical source data.

## Data Availability

All data that support the findings of this study are available from the corresponding author. We provide the numerical source files of all figures in the supplementary material. Our training and test data as well as the trained network is available in GitHub at https://github.com/erturklab/delivr_cfos (ref. ^60^) and https://github.com/erturklab/delivr_train (ref. ^61^). A subset of representative whole-brain scans is available at the EBI Bioimage Repository (accession code S-BIAD1019). Due to limitations to share large imaging data online, additional whole-brain scans (*n* = 27 whole brains, ~2 TB data) will be made available upon reasonable request. The Allen Brain Atlas (CCF3) was downloaded from the Scalable Brain Atlas repository at https://scalablebrainatlas.incf.org/mouse/ABA_v3. Source data are provided with this paper.

## Code availability

All code to run our pipeline end-to-end is available in GitHub at https://github.com/erturklab/delivr_cfos (ref. ^60^). Training code is available in GitHub at https://github.com/erturklab/delivr_train (ref. ^61^). Docker containers and the plugin can be obtained from https://discotechnologies.org/DELiVR. The code is released under the MIT license.
