# Supplementary material for: Virtual reality-empowered deep-learning analysis of brain cells
Source: Nat Methods. 2024 Apr 22;21(7):1306–15. doi: 10.1038/s41592-024-02245-2 (PMC11239522; doi:10.1038/s41592-024-02245-2)
Supplement: Supplementary file 1 — Supplementary Fig.1, Supplementary Table 1 and DELiVR handbook. [file 41592_2024_2245_MOESM1_ESM.pdf]

nature methods

Article https://doi.org/10.1038/s41592-024-02245-2

Virtual reality-empowered deep-learning analysis of brain cells

In the format provided by the authors and unedited

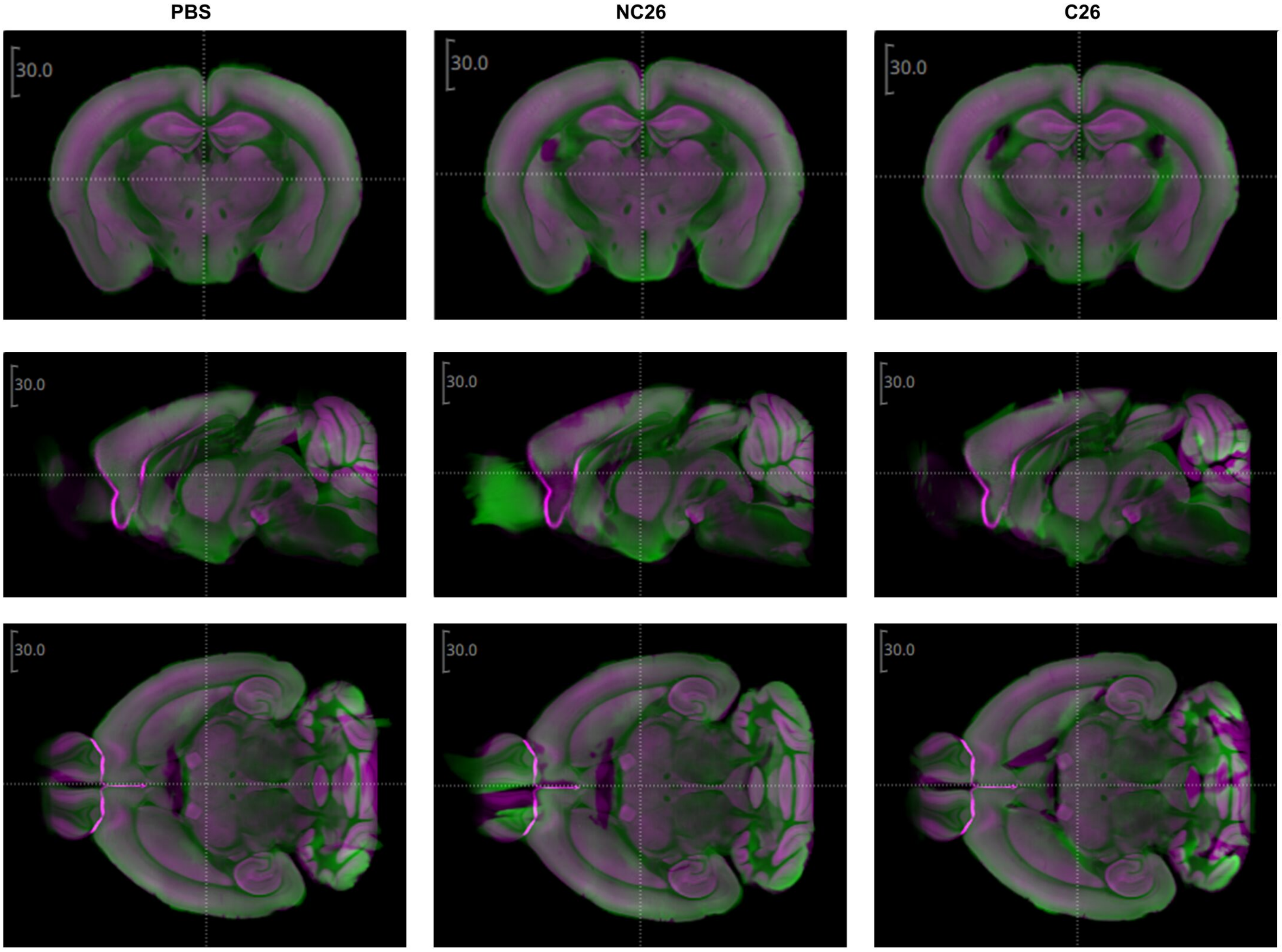

Supplementary Figure 1. Atlas Registration with mBrainAligner. Post-registration overlap for representative brains of the experimental groups PBS (**left**), NC26 (**middle**) and C26 (**right**), visualized with Vaa3D. Green, registered image stack. Magenta, mBrainAligner's version of the CCF3 reference atlas.

Supplementary Table 1: Quantitative comparison between previous methods of cell detection, different deep learning segmentation models and DELiVR based on volumetric Dice, Precision and Sensitivity.

Method	Volumetric Dice	Volumetric Precision	Volumetric Sensitivity
DELiVR	0.6739	0.7946	0.5975
Clearmap	0.0317	0.3467	0.0168
Clearmap2	0.0989	0.6289	0.0827
Optimized ClearMap	0.4747	0.8539	0.3734
Ilastik	0.3584	0.2463	0.7929
DynUt	0.6422	0.7817	0.5604
SegResNet	0.6338	0.7295	0.5919
Transformer Unet	0.6583	0.7141	0.6466

DELiVR – Handbook

This handbook should serve as a guidance to implement the computational part of DELiVR. Please also refer to our other resources and updates at https://discotechnologies.org/DELiVR.

Contents

1. Annotation of ground truth data using virtual reality 2
  1.1. Data preparation 2
  1.2. System requirements 2
  1.3. Arivis VisionVR 2
    1.3.1. Importing data into Arivis VisionVR 2
    1.3.2. General instructions using the VR-headset and controllers in ArivisVision VR 5
    1.3.3. Annotation of data using Arivis VisionVR 7
    1.3.4. Exporting annotations 16
    1.3.5. Helpful tips for annotation / visualization using Arivis VisionVR 18
  1.4. syGlass 18
    1.2.1. Import data into syGlass 18
    1.4.1. General instructions using VR-Headset and controllers in syGlass 24
    1.4.2. Annotating data using syGlass 28
    1.4.3. Export annotations in syGlass 36
    1.4.4. Helpful tips for annotation / visualization in syGlass 37
2. Step-by-Step guide for using DELiVR 40
  2.1 System requirements 40
  2.2. Data preparation 40
  2.3. Installation 40
    2.3.1 Installing DELiVR Docker image (required for Fiji plugin) 40
    2.3.2 Fiji Plugin 43
  2.4 Running DELiVR end-to-end 43
  2.4.1 The config file 43
    2.4.2 Running DELiVR from Docker (optional) 48
    2.4.3 Running DELiVR using the Fiji-Plugin (preferred) 49
    2.4.4 Validation of segmentation output in image space and atlas space 53
    2.4.5 Training DELiVR for your custom dataset using the Fiji Plugin 54
  2.5 Troubleshooting DELiVR 56

1. Annotation of ground truth data using virtual reality

Here, we describe the annotation of ground truth data using two commercial software packages: ArivisVisionVR and syGlass. We used both software packages in combination with a VR-Head-Set (Oculus Rift). We would like to note, that this annotation can theoretically be done in any VR environment, as the only required output is the ground truth data.

1.1. Data preparation

The data we chose to annotate consisted of a 100x100x100 voxel cube of a mouse brain, that was antibody labeled for c-Fos, cleared and imaged using light-sheet fluorescent microscopy.

1.2. System requirements

Both, ArivisVisionVR and syGlass are compatible with a standalone workstation setup equipped with 32GB RAM, Windows 10, high-end nVidia video card and an Oculus/Meta VR headset. For specific parameters refer to the https://www.syglass.io/#requirements and the https://kb.arivis.com/vision-vr-system-requirements addresses.

1.3. Arivis VisionVR

The Arivis VisionVR software is a virtual reality (VR) environment that, amongst other features, can be used for data exploration and data annotation. Here, we provide a basic description on using Arivis VisionVR for annotating data in VR. For additional information and explanations, please also refer to the Website of the provider: https://kb.arivis.com/arivis-visionvr-quick-start-guide .

1.3.1. Importing data into Arivis VisionVR

- Open the Arivis VisionVR software by clicking the Arivis VisionVR Icon on the desktop after installation. The starting window will appear.

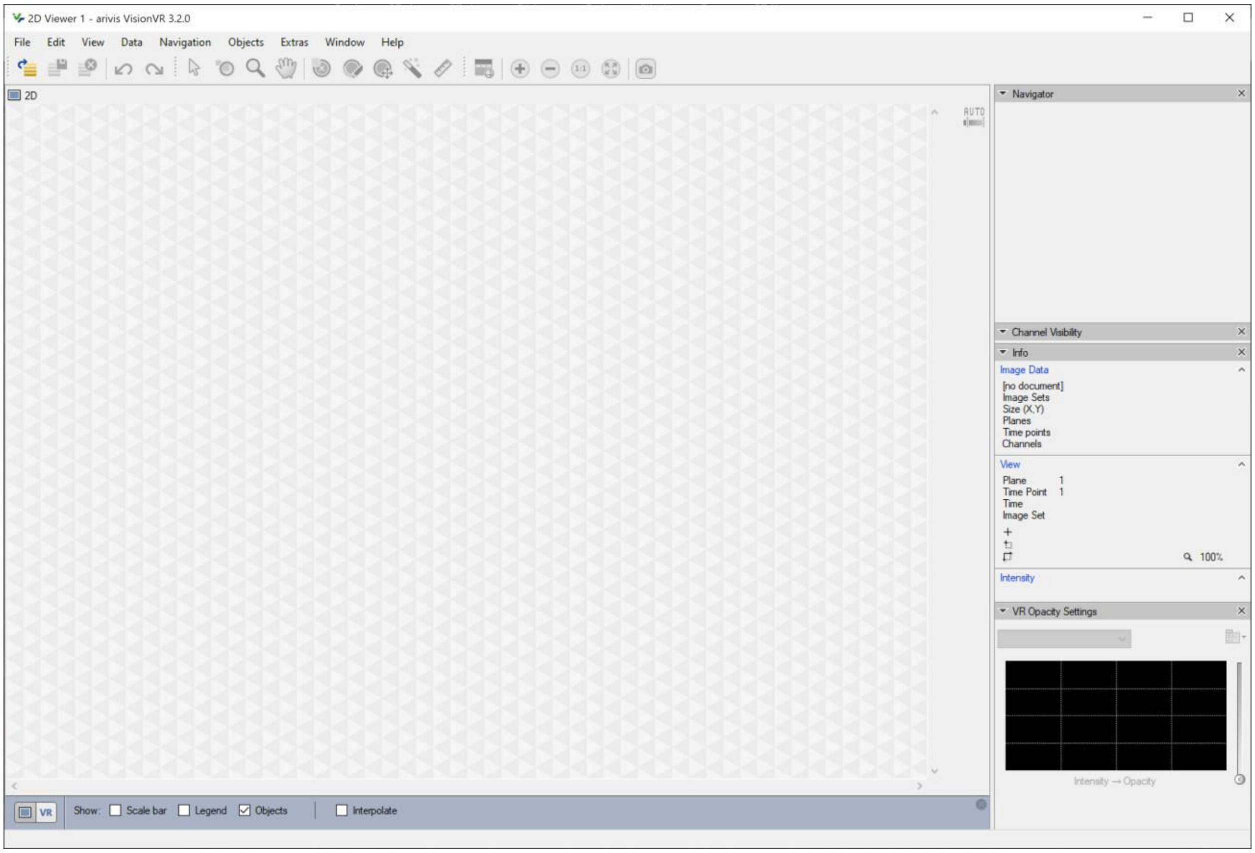

- Import the data set of interest that should get labeled in virtual reality into Arivis VisionVR by importing raw image data files by clicking on "File" → "Import".

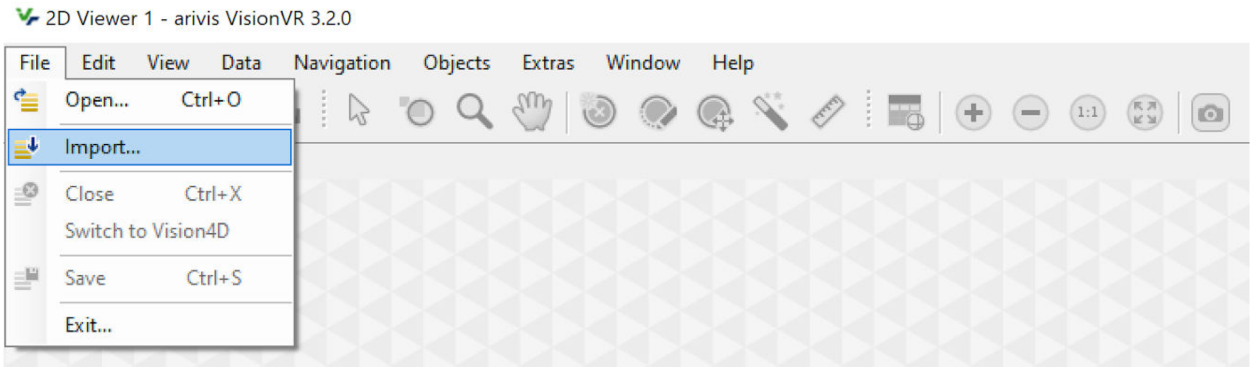

- In the "Import Images" – window, select your imaging data and click "Open". The "Import files"- window will appear. Select "New File" and choose a saving destination for the generated Arivis SIS.file. Finalize by clicking on "OK".

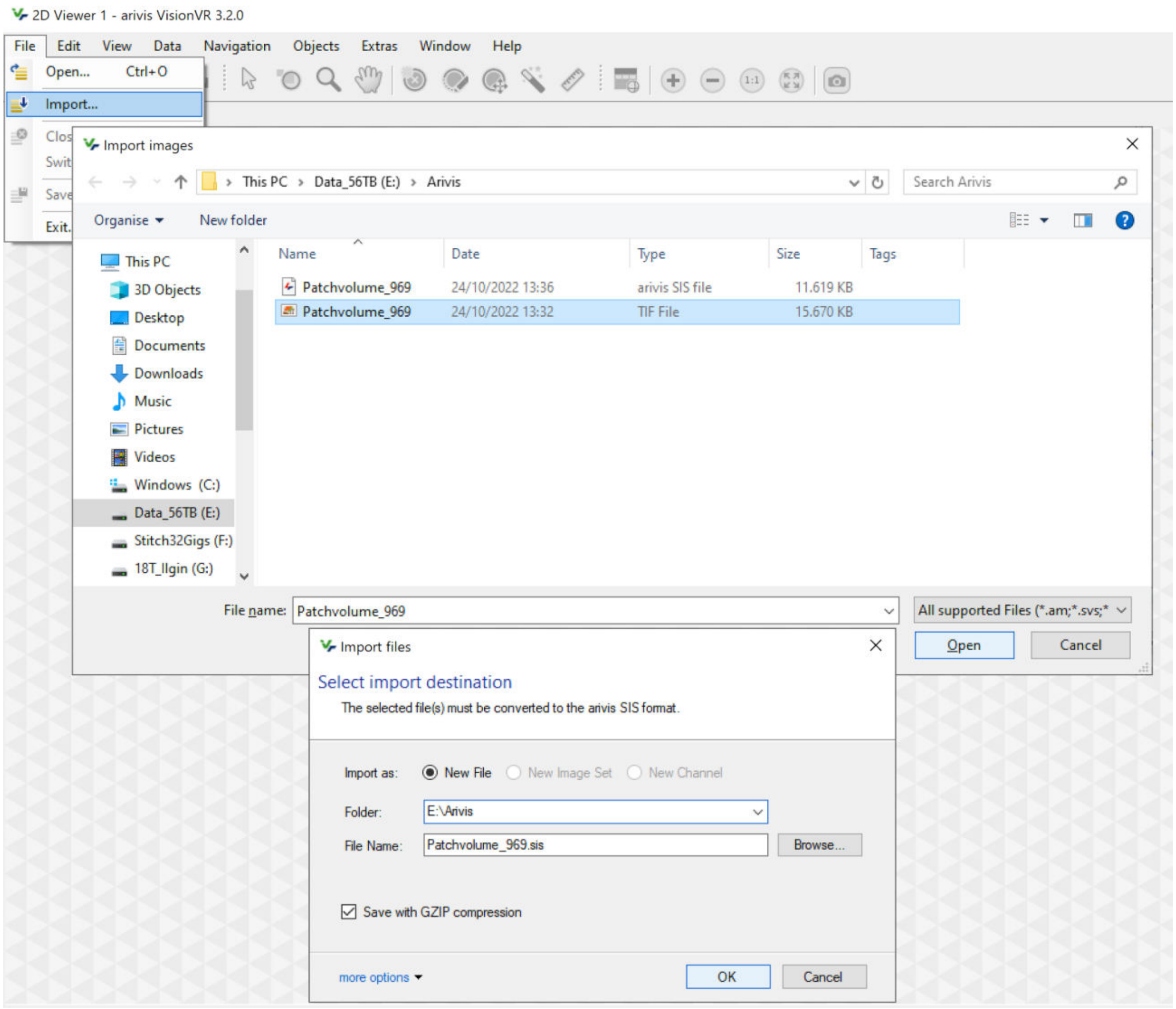

- Your image set will appear in the Arivis VisionVR window as a 2D plane after the import.
  - The contrast of the images can be adjusted with the bar next to the window.
  - You can use the cursor on the right side of the window to scroll through the image stack.

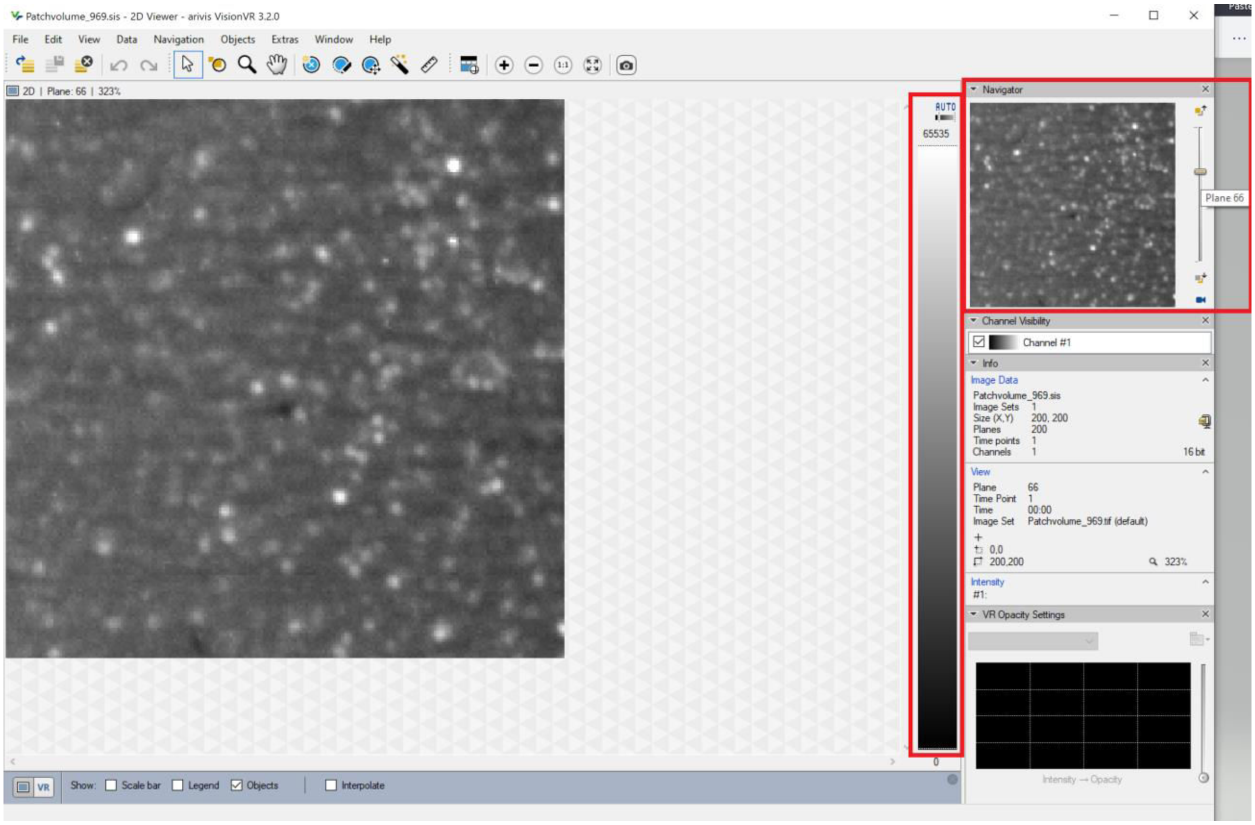

1.3.2. General instructions using the VR-headset and controllers in ArivisVision VR

Our description of the VR-Headset and controller usage are based on the Oculus Rift with Oculus Touch controllers, which we used for our annotations. However, when using other VR headsets, please note that controller functions can be slightly different.

- Key assignments of the controls can be found in Table 1.
  - By pressing the **B** button, the main menu opens.
  - The **Side trigger** is used to move the 3D-file.
  - With the **Front trigger** on the right controller, you select, click and use the tools.
  - By pressing the **Joystick** (left), the tool menu opens/closes.
  - The **X** button can be used to decline selections, undo deletions or separation of objects.
  - The **A** button is used to confirm the selection.

Table 1: Controller settings / Key assignments

B	Open/close settings menu
X	Decline / undo cuts or delete
A	Confirm
**Joystick** (left)	Open/close tool menu
**Joystick** (right)	Change ROI/grid size
**Front trigger**	Select / click
**Side trigger**	Move/rotate/zoom

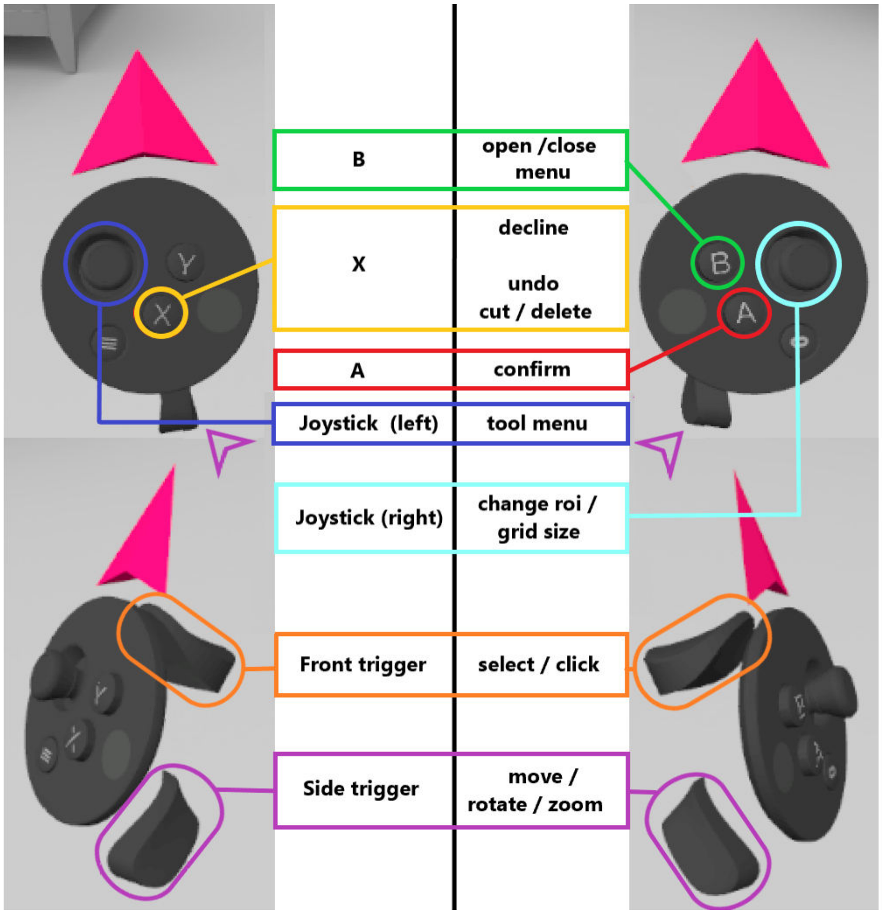

1.3.3. Annotation of data using Arivis VisionVR

- Switch to VR by clicking on “VR” in the bottom left corner of the screen.

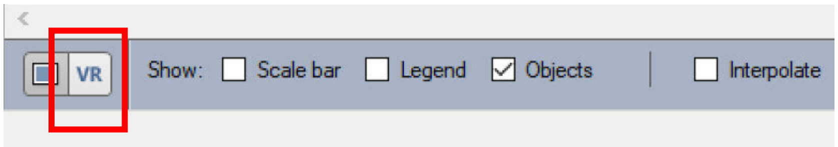

- Put on the VR-headset (eg. Oculus Rift) and use the handheld controllers to navigate through the data and the software.
- When entering the Virtual Room select “Segmentation”.

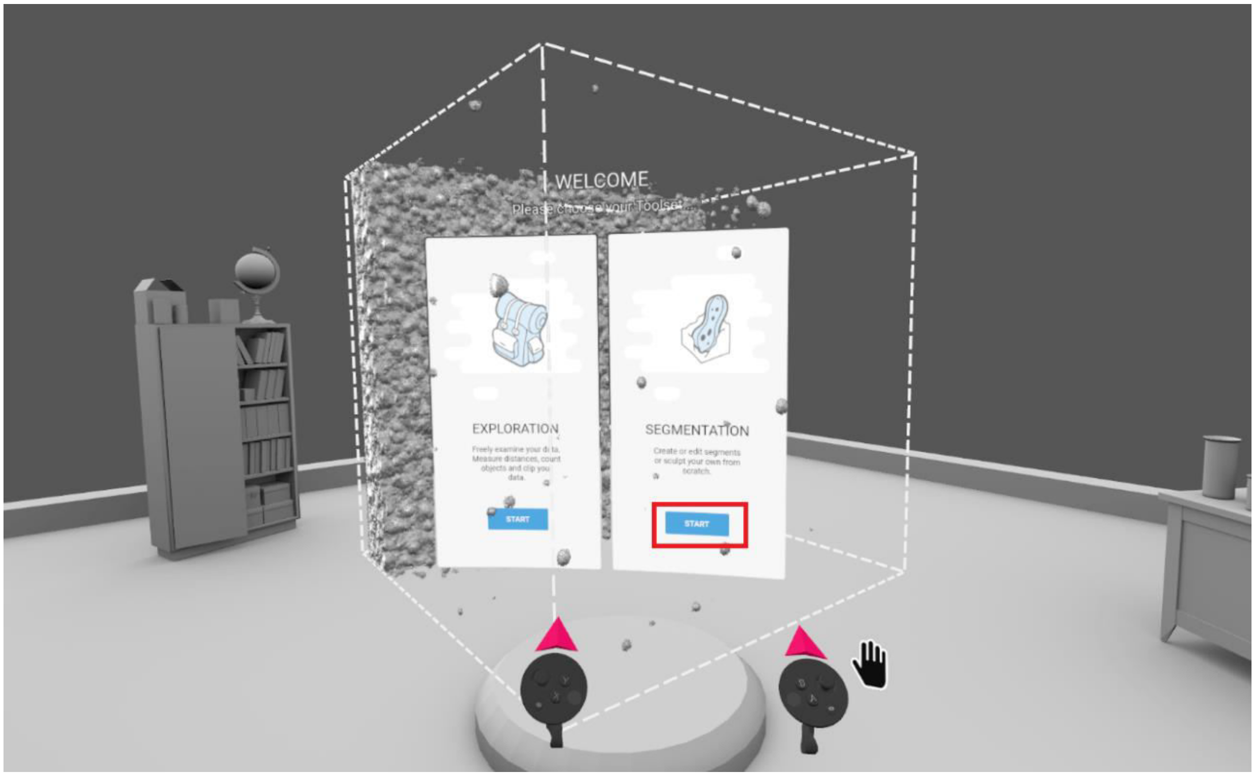

- The 3D- Volume will become accessible and can be moved by holding the **Side trigger** of one or both controllers.
  - Rotating the 3D-Volume works by holding the **Side triggers** of both controllers and turning the controllers as if you were holding an actual object.
  - For zooming out/in of the 3D-volume, hold the **Side triggers** of both controllers and move them close together or apart.

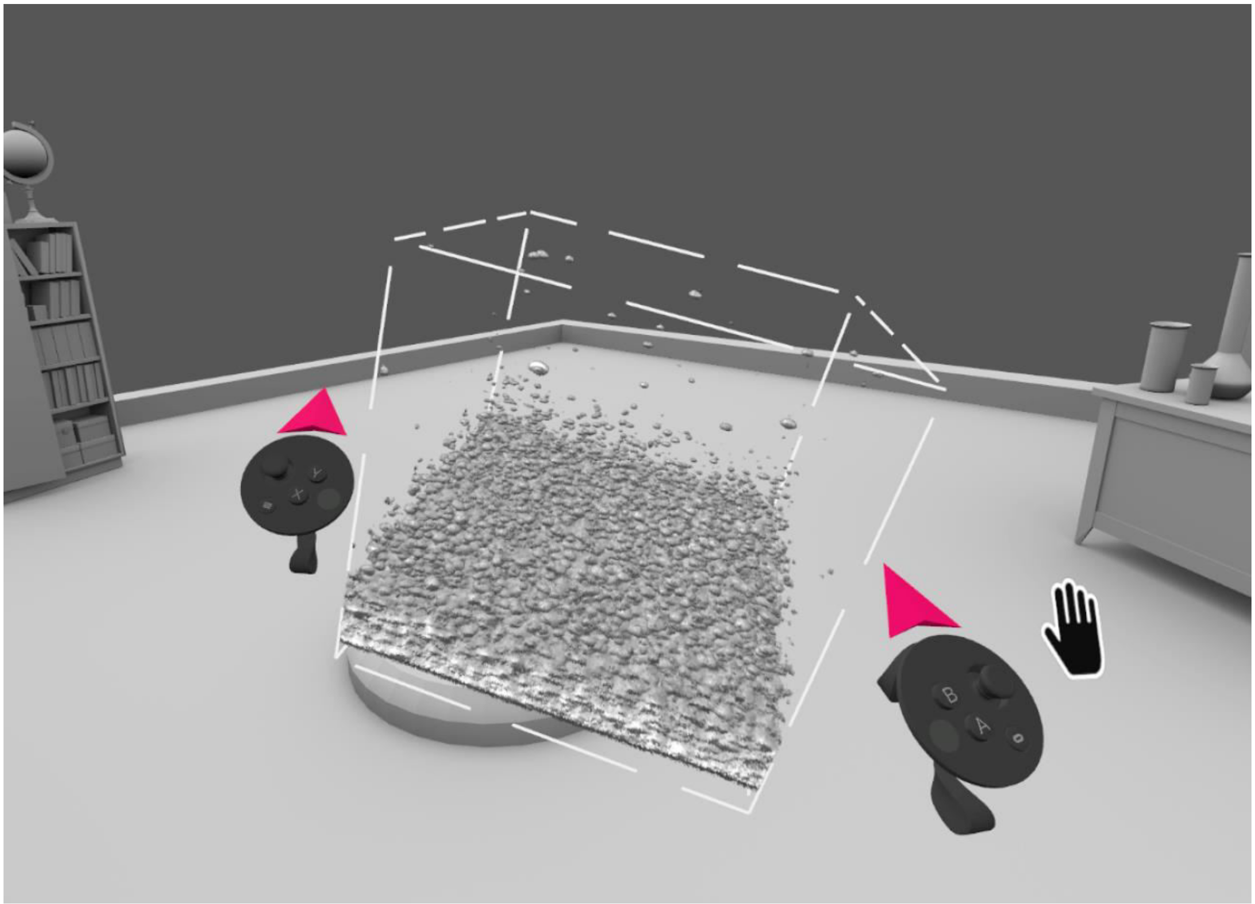

- Click on **B** on the right controller to open the main menu to adjust the visual appearance of your data volume.
    - In the main menu, select the graph symbol to change the opacity curve. Thereby, you can change the appearance of your data volume.
        - Use the pointer and click and hold in the white area that is right or left of the opacity curve to move the whole graph.
        - Use the pointer and click and hold lines or points in the opacity graph to change the settings.

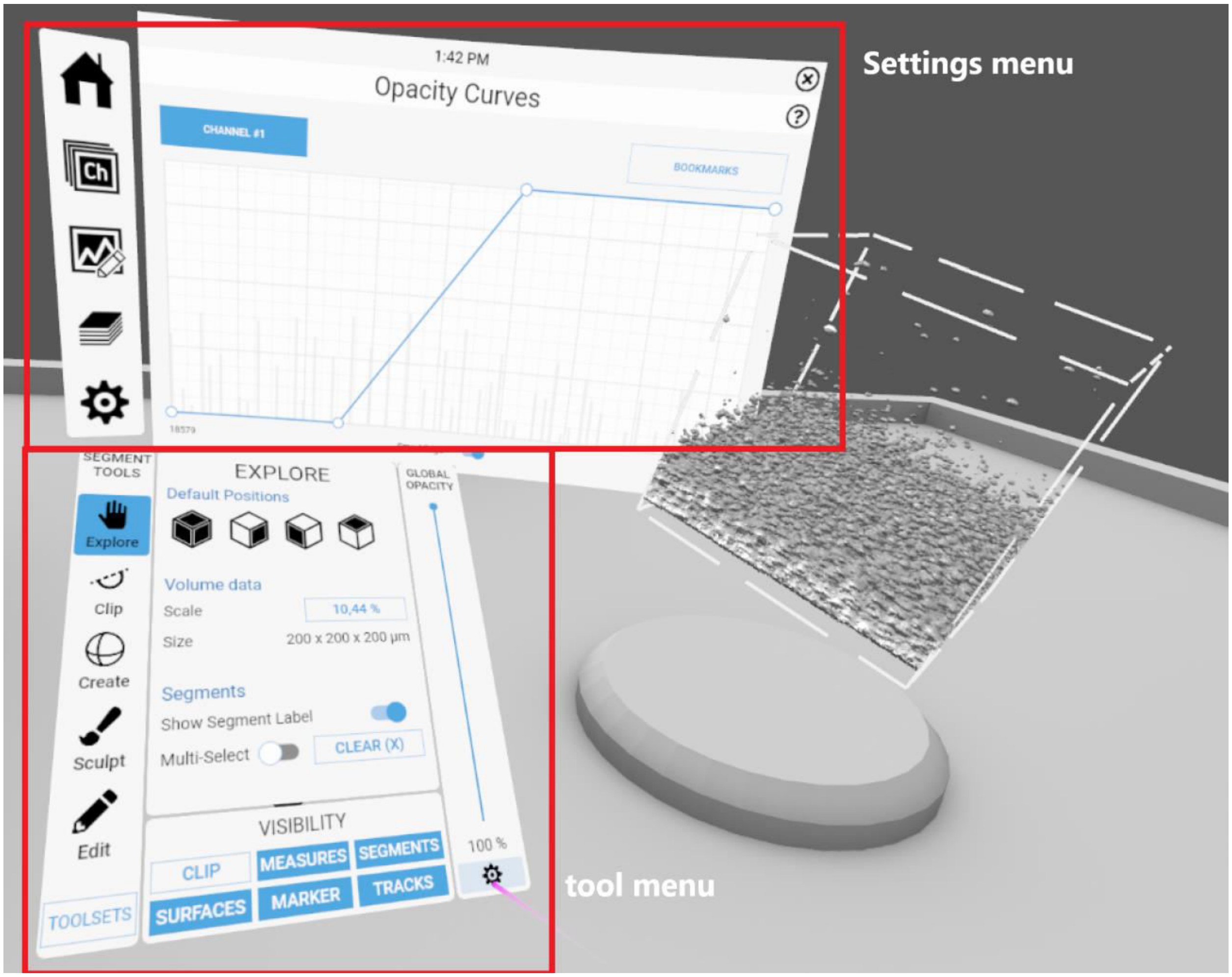
Settings menu
1:42 PM
Opacity Curves
CHANNEL #1
BOOKMARKS
SEGMENT TOOLS
EXPLORE
Default Positions
GLOBAL OPACITY
Explore
Clip
Create
Sculpt
Edit
TOOLSETS
Volume data
Scale
10,44 %
Size
200 x 200 x 200 µm
Segments
Show Segment Label
Multi-Select
CLEAR (X)
VISIBILITY
CLIP
MEASURES
SEGMENTS
SURFACES
MARKER
TRACKS
100 %
tool menu

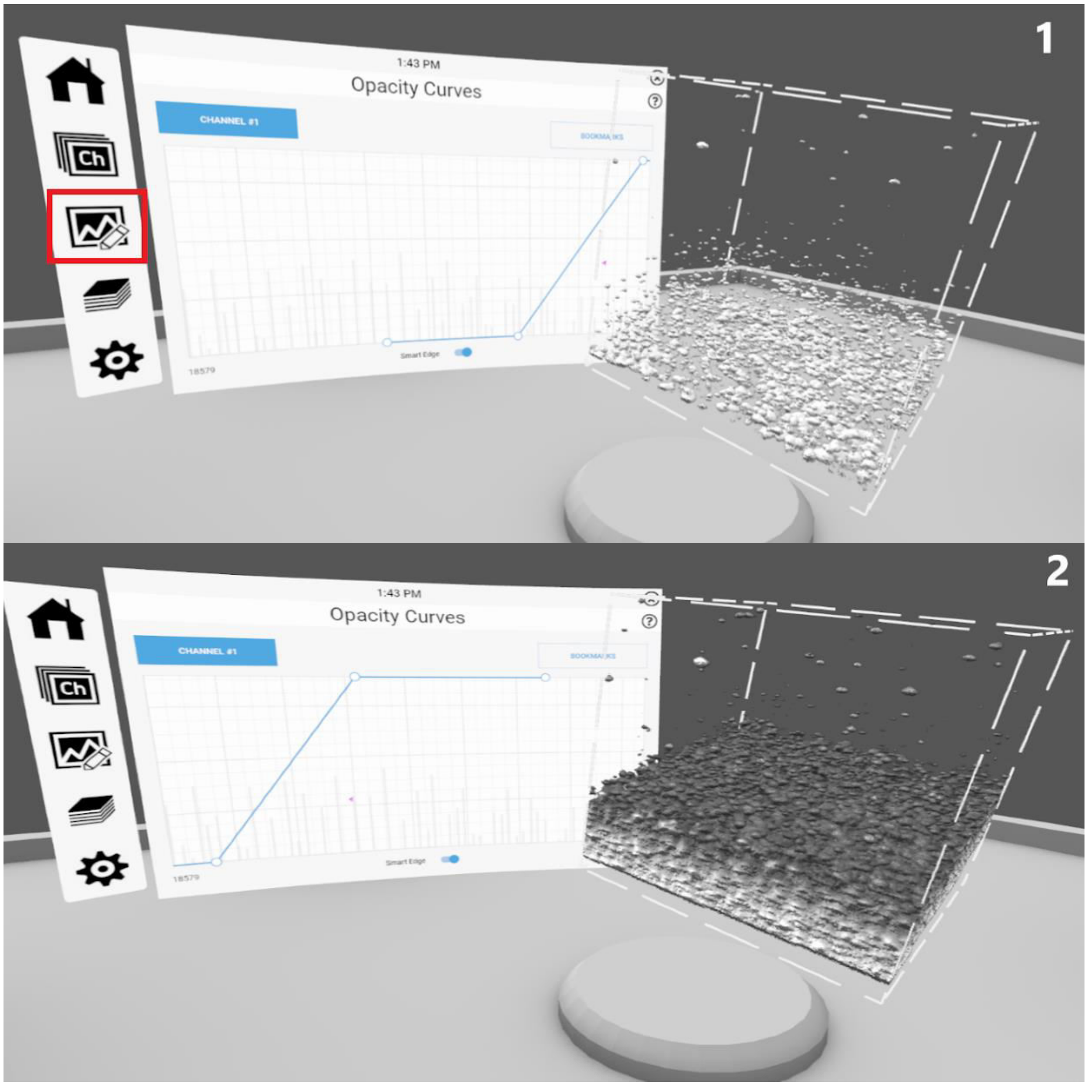

- The settings symbol (cog wheel) leads you to different volume renderings.
    - For annotating dense object, “Maximum intensity” works well.

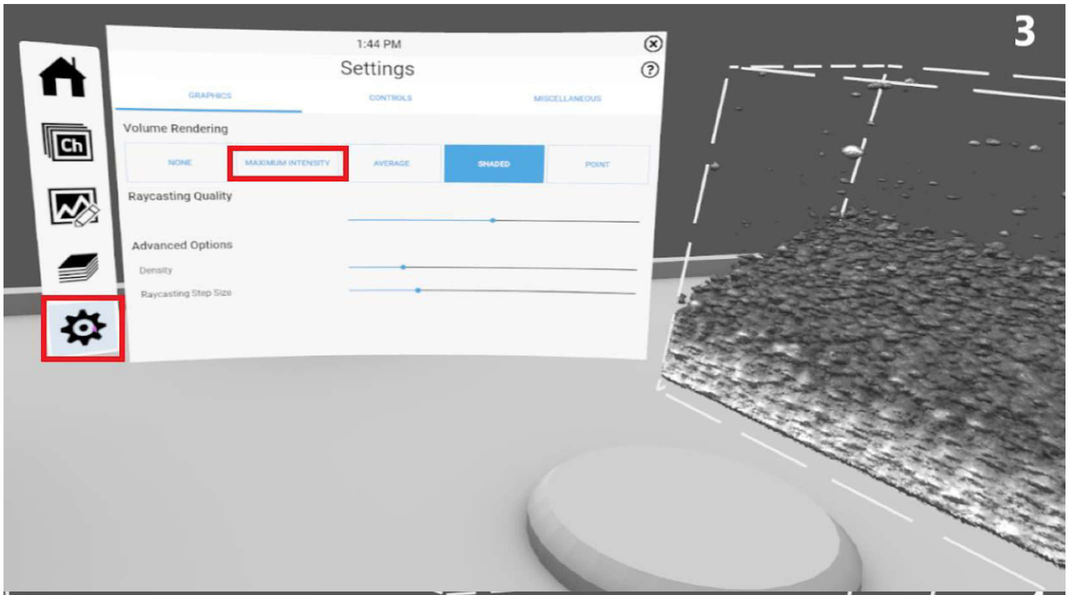

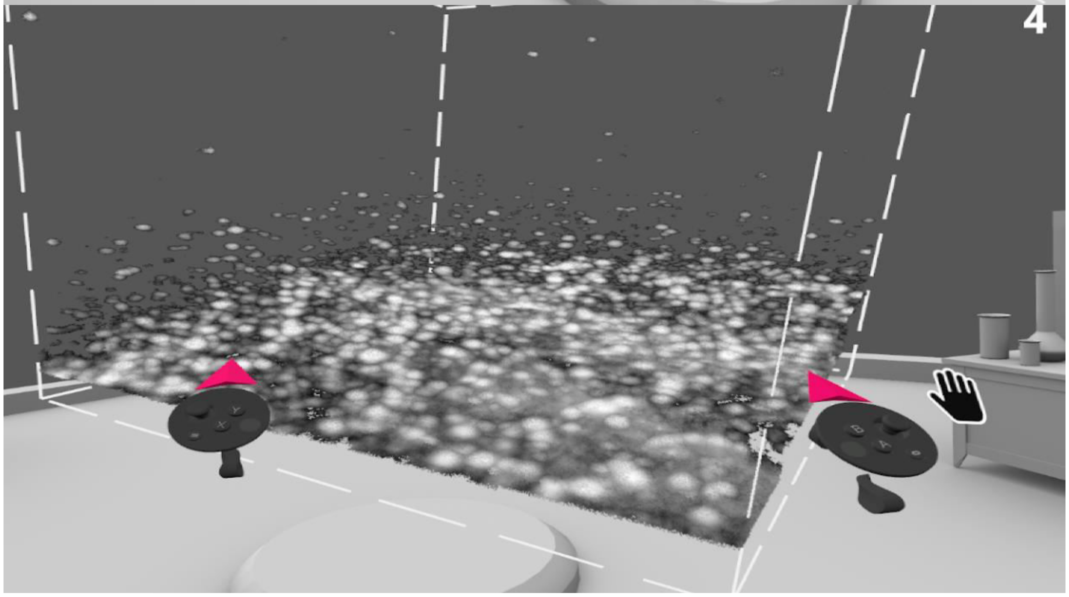

- To start the annotation, open the tool menu clicking the **left joystick**.
  - In the Tool menu, select “Create” and the magic wand symbol for semi-automated annotation.
  - For full manual annotation, select “Sculpt”.

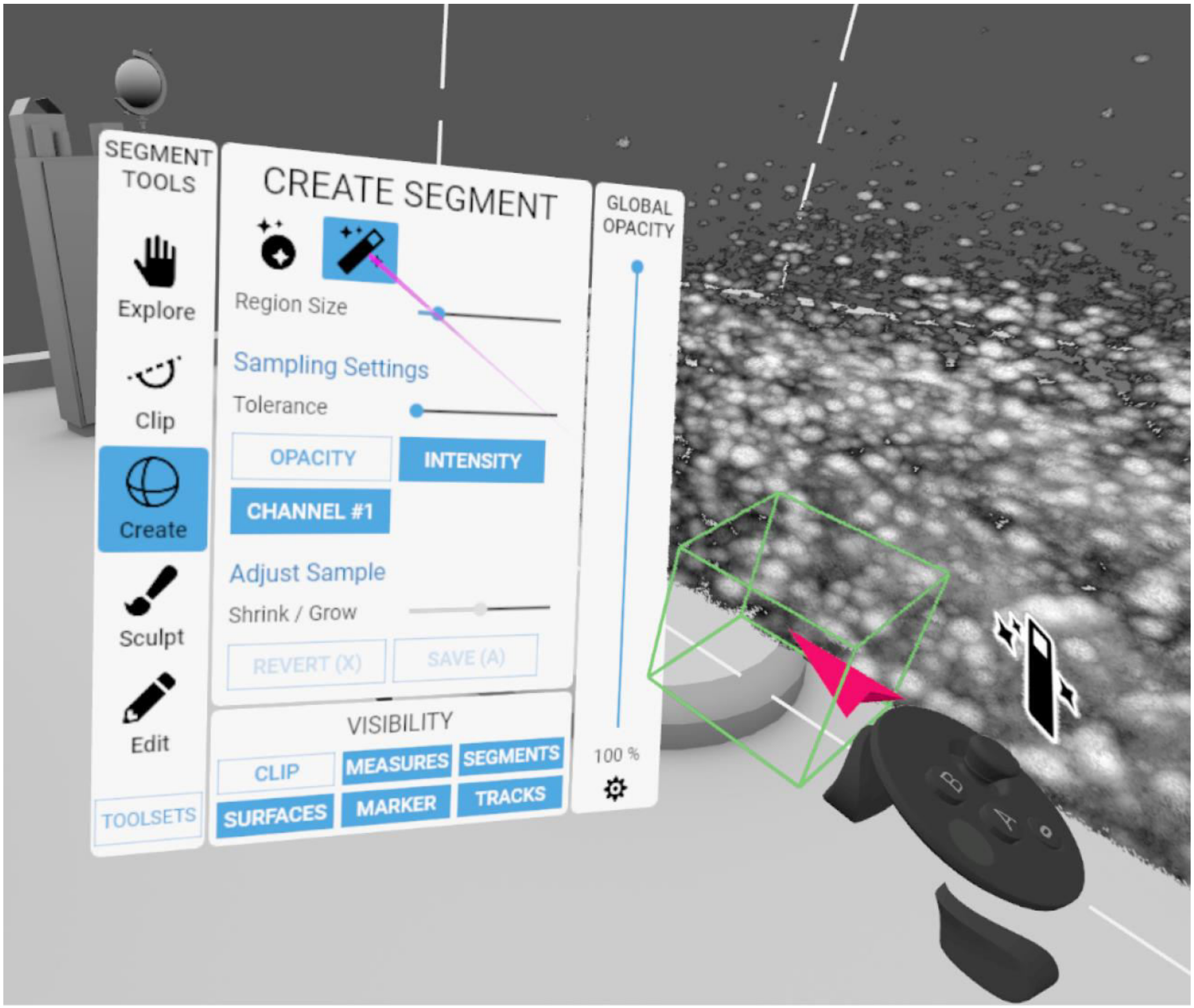

- When the magic wand symbol is selected, a green cube will appear in the right controller that will be used to annotate objects of interest.
  - The green cube can be adjusted in size by moving the right joystick up or down.
- For annotating, use your right controller to place the green cube around the object you would like to annotate and click the **Front trigger**.
  - Try to point the red arrow on your controller in the middle of the object.
- After clicking the **Front trigger**, a grid will be created around the object.
  - The grid can be adjusted in size by moving the **Right joystick** up or down.
  - If the grid is not visible right away, make the grid bigger or try to set your cube selection again with the red arrow placed a bit off the center of the signal.

- Adjust this grid to a size that covers your signal accordingly and click “**A**” to confirm your selection.
    - The confirmation will render a 3D object in the shape of the grid at the position of your signal.
    - Sometimes, a grid slightly smaller or bigger than your object can result in a better rendered object.

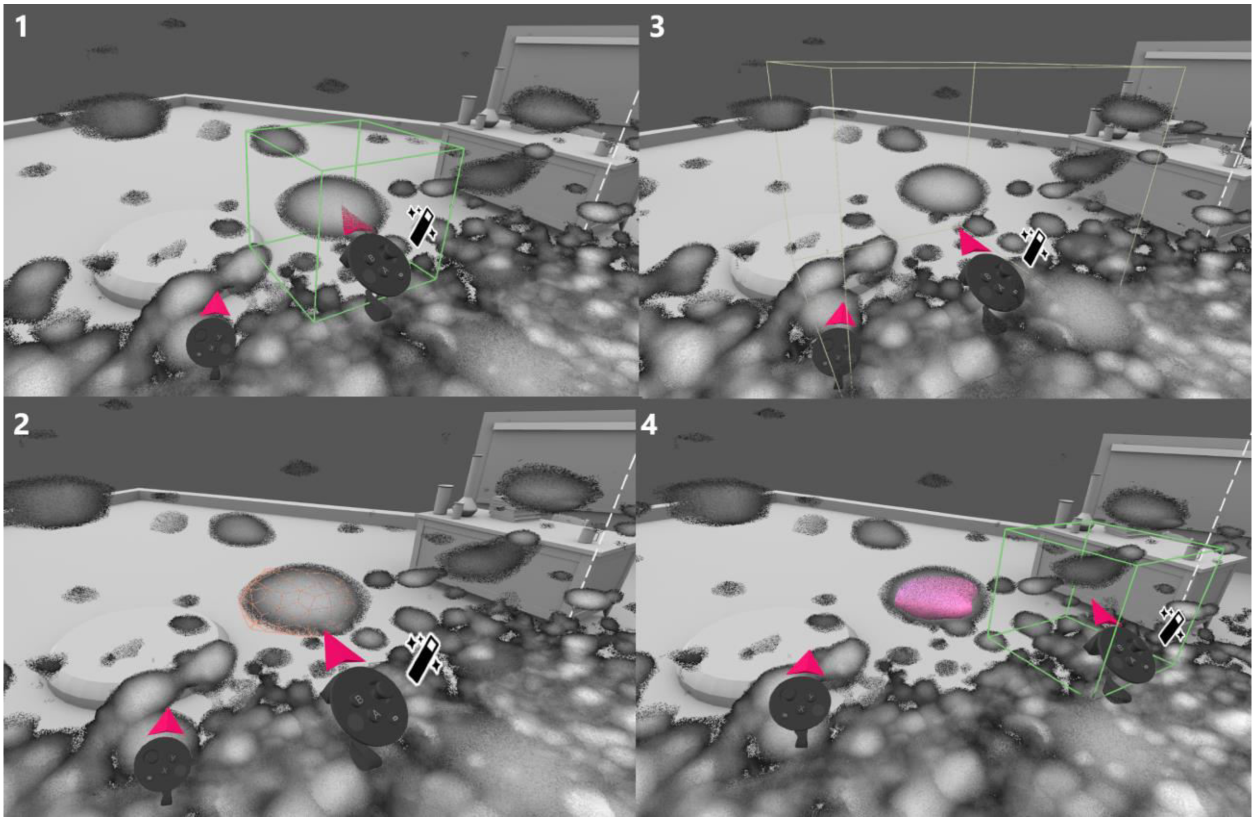

- To delete objects, select “Edit” in the tool menu and then select the trash can symbol.
    - Select all objects by pointing with the small laser pointer of the right controller and using the front trigger.
        - Selected objects will have a small cube around them.
    - Click **A** to confirm that selected objects will be deleted
    - By clicking **X** twice times, recent deletions can be recovered.
        - If you created new objects in the meantime, deletions will not be recovered anymore.

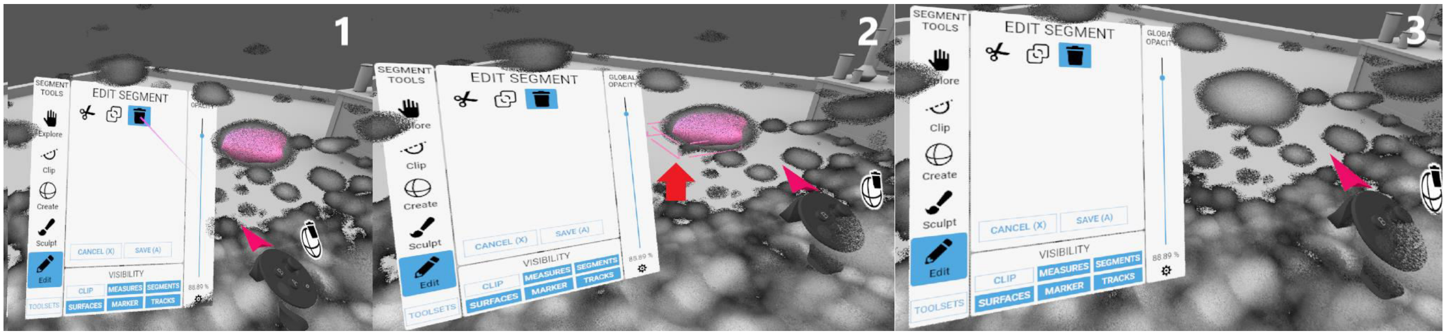

- To cut an annotation of an object in to smaller pieces (for example when two objects are touching each other), select "Edit" in the tool menu and then select the scissors symbol.
  - Select the annotation by placing the plane of your right controller and use the **Front trigger** to fix it in the right place for your cut.
  - Selected annotations will have a small cube around them.
  - By clicking **A**, you confirm that the selected object will be cut.
  - By clicking **X** twice, recent cuts will be undone.
    - If you created new annotations in the meantime, cuts will not be able to undo anymore

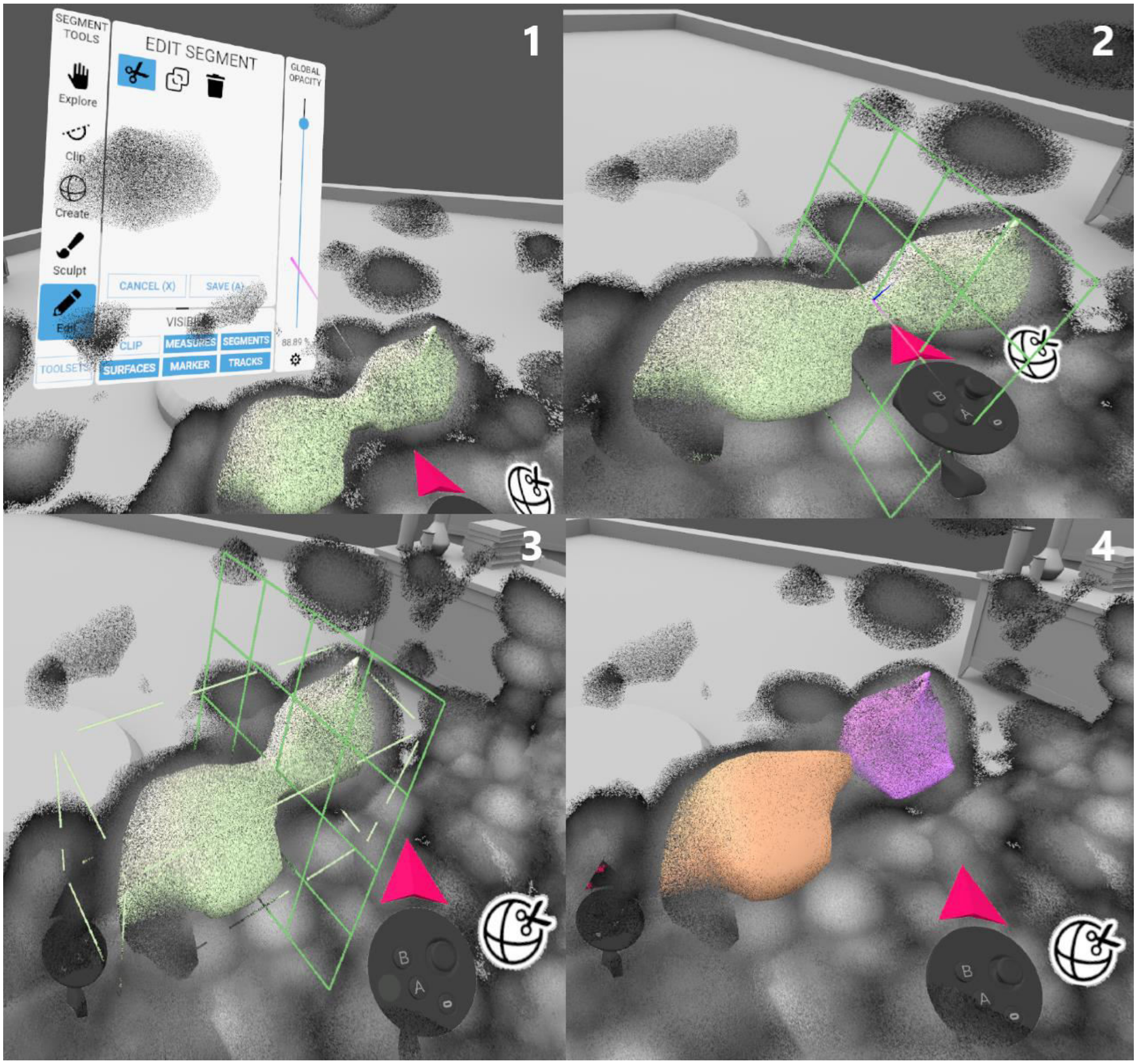

- Continue your annotations for the remaining objects and save the annotation at the end.

- You can save your annotation at any point and return to it later to continue.

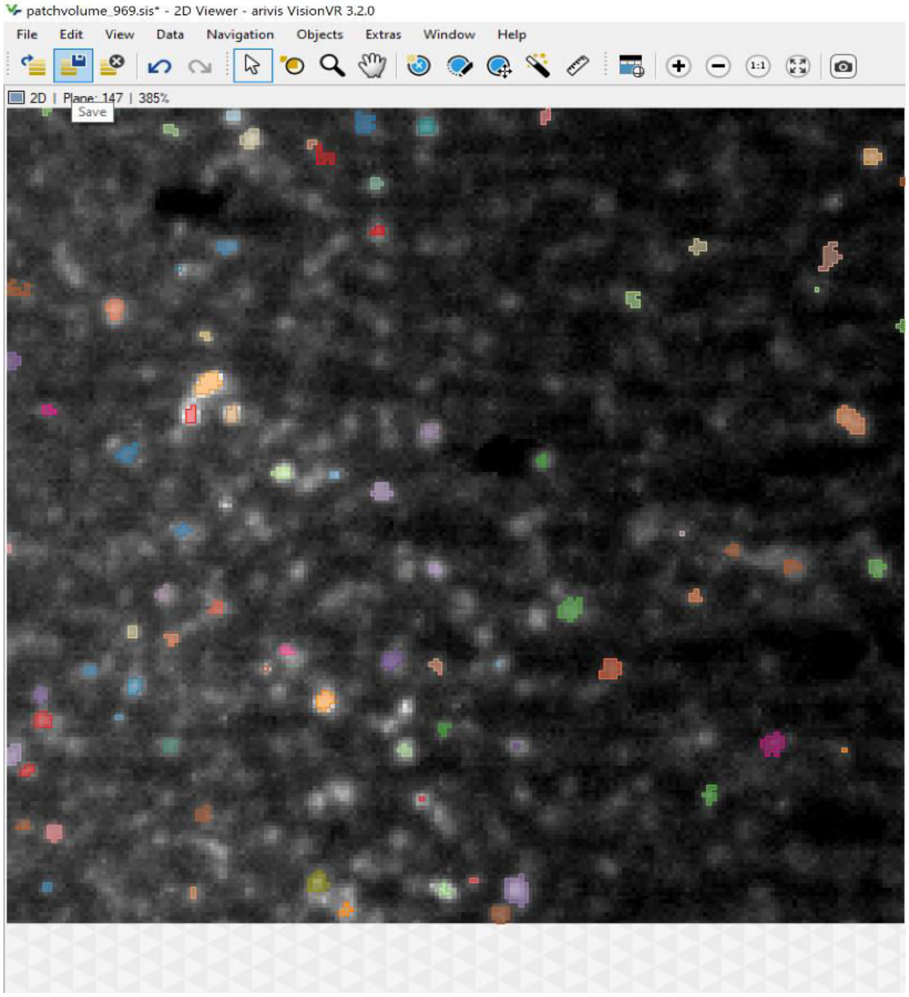

1.3.4. Exporting annotations

To export annotations made in Arivis VisionVR, it is necessary to switch to Arivis Vision4D that has an Import/Export Objects package. Therefore, the procedure below describes the export procedure in Arivis Vision4D.

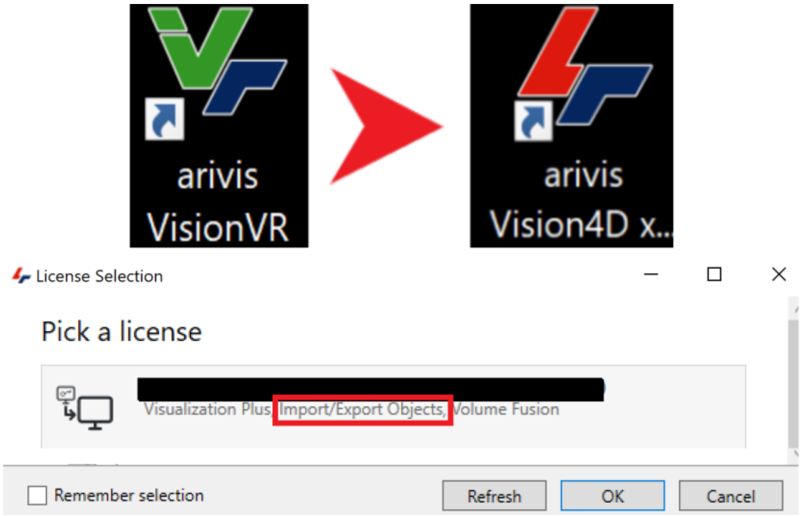

- Save your annotation in Arivis VisionVR to the .sis file and open it in Arivis Vision4D.
- Select objects in the top task bar.
    - Select "Export Objects to Labeled Images"
        - Select saving location and click on "Export"
- Your annotation should be now be saved as Tiff-Stack.

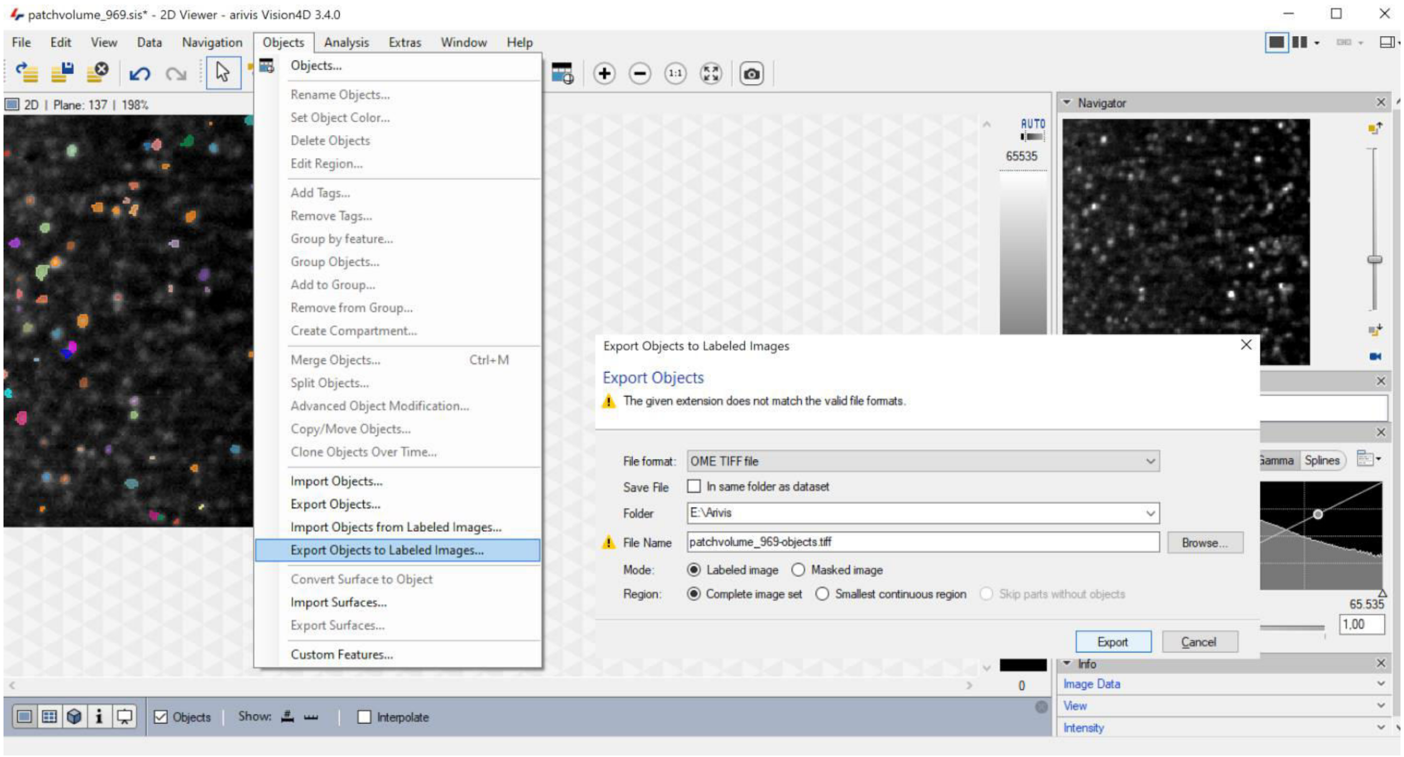

1.3.5. Helpful tips for annotation / visualization using Arivis VisionVR

- Try to change your perspective with your 3D data. This helps a lot to identify your signal, false negatives / positives or artifacts.
- You can try to dive into the data set by changing the perspective from outside of the volume to inside of the volume.
- Close the Main menu and tool menu after changing the visual settings or selecting the tool, to avoid accidental clicking on the menus (you will not be able to annotate if your controller points at the menus).

1.4. syGlass

The syGlass software is a virtual reality (VR) environment that, amongst other features, can be used for data exploration and data annotation. Here, we provide a basic description on using syGlass for annotating data in VR. For additional information and explanations, please also refer to the **syGlass User Guide**.

1.2.1. Import data into syGlass

- Open the syGlass software by clicking the syGlass Icon after installation. The starting window will appear.

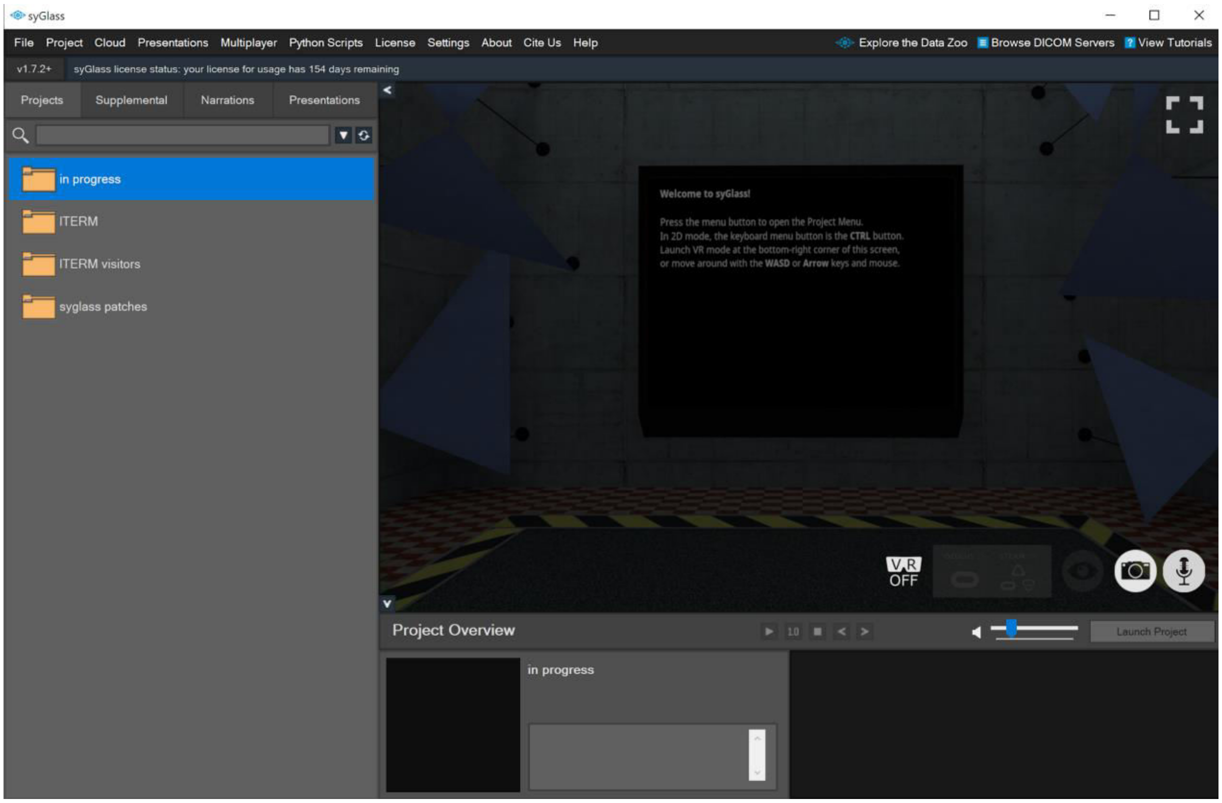

- Import the data set of interest that should get labeled in virtual reality into the syGlass software by importing raw image data files by clicking on “File” → “Create Project” → “Create New Project”.

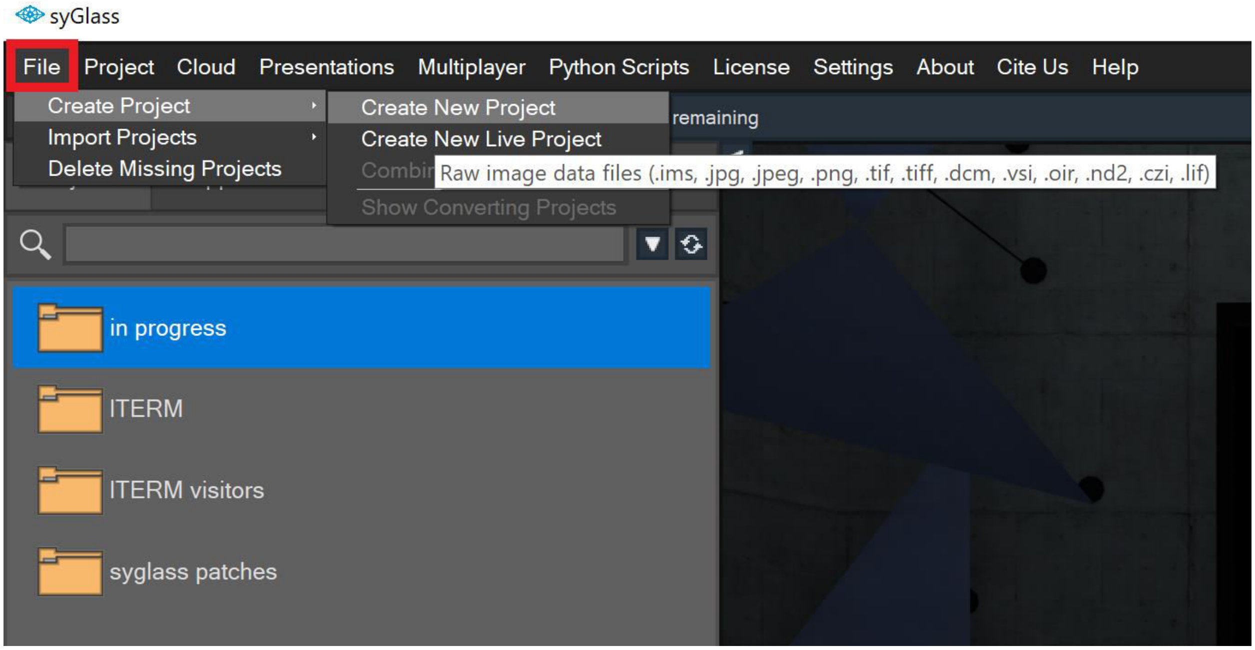

- Import existing projects by clicking on “File” → “Import Projects” → “Import Existing Projects”.

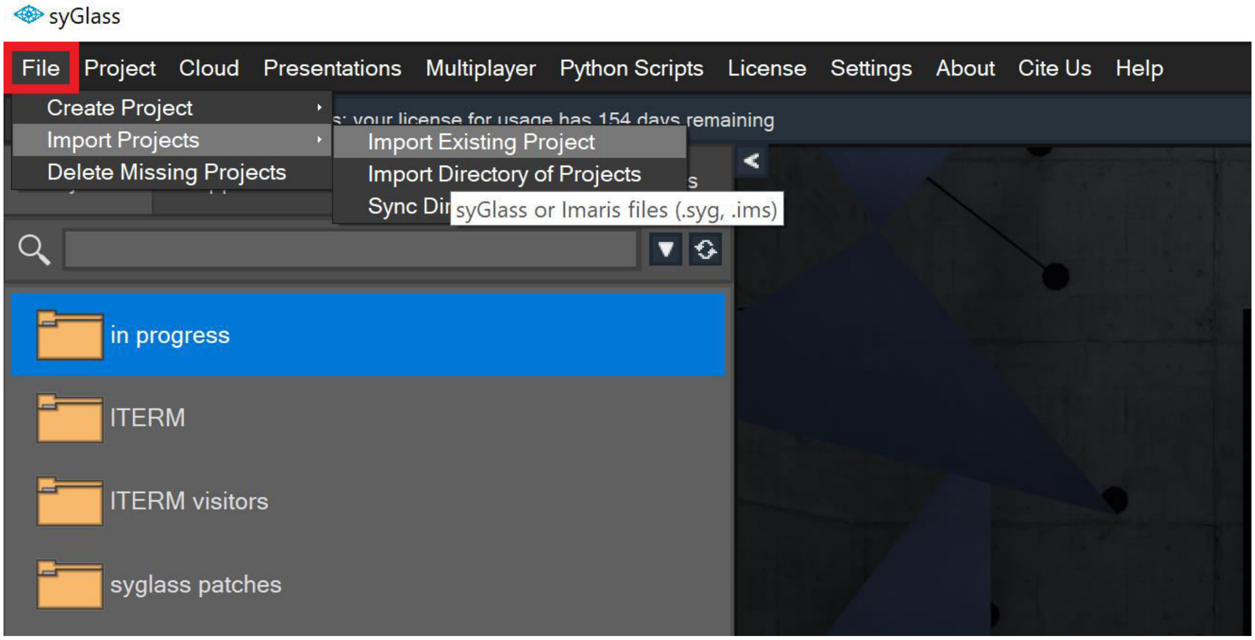

- In the “New Project Wizard”-window, click on “Add Files” and choose the imaging data set in the browser by clicking “Open”. The window also shows the compatible file formats. For Tiffs, use an image sequence to avoid size limitations of 4GB for the import.

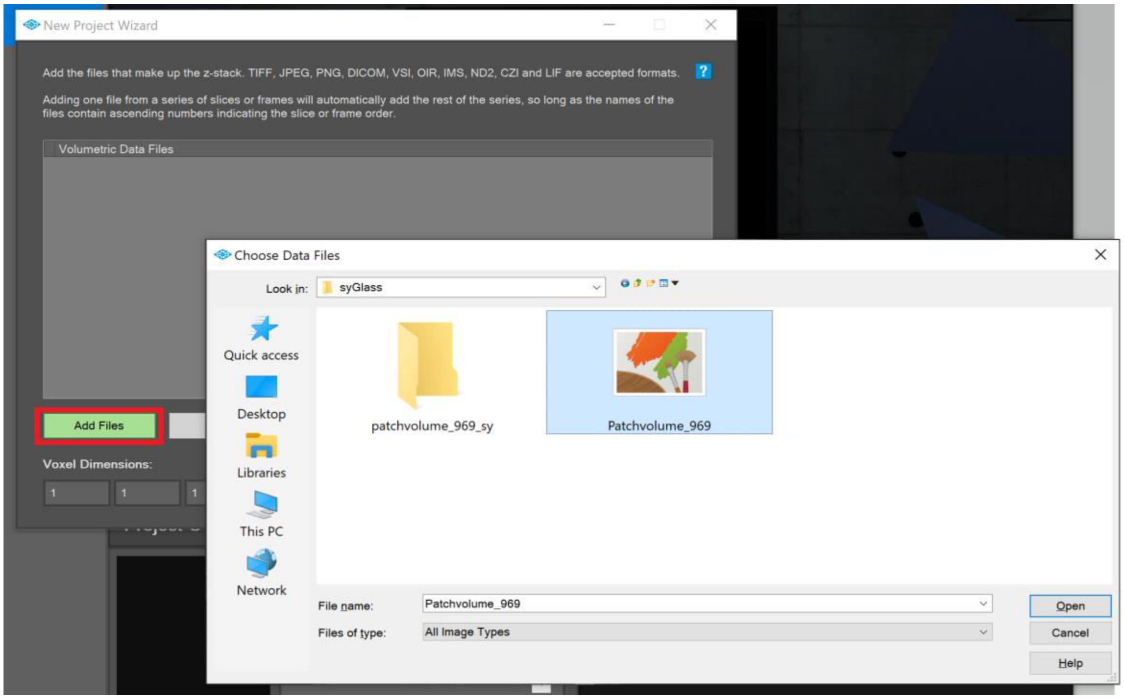

- Press “No” in the Window “Confirm Time Series”.

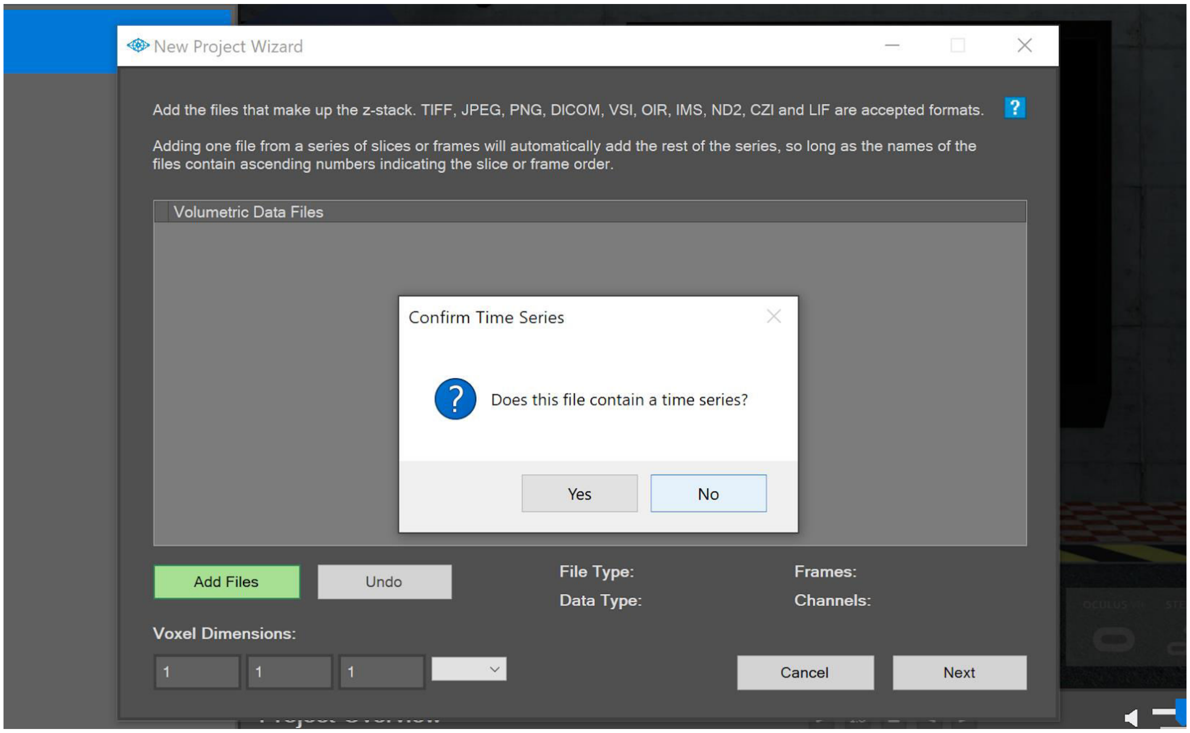

- The path of your imaging data set will appear under “Volumetric Data Files” in the “New Project Wizard” - window. The correct channel count will be visible. Change the voxel dimensions and scale if needed to fit the resolution of your data and click “Next”. Both can also be changed at a later time point.

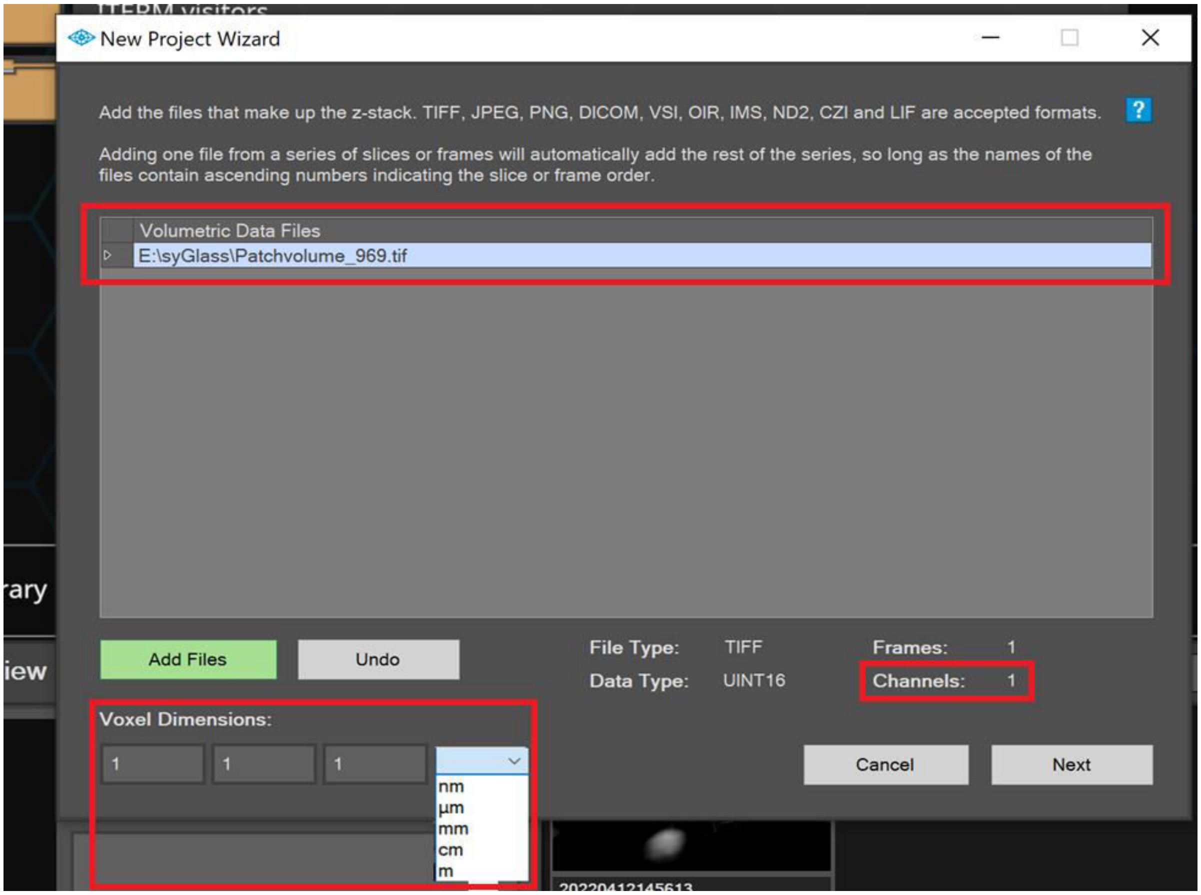

- Choose downsampling options (syGlass recommends to downsample the data to 8-bit) and press "Save". Select the saving folder, name your file and press "OK". The import of the data into the syGlass format will start.

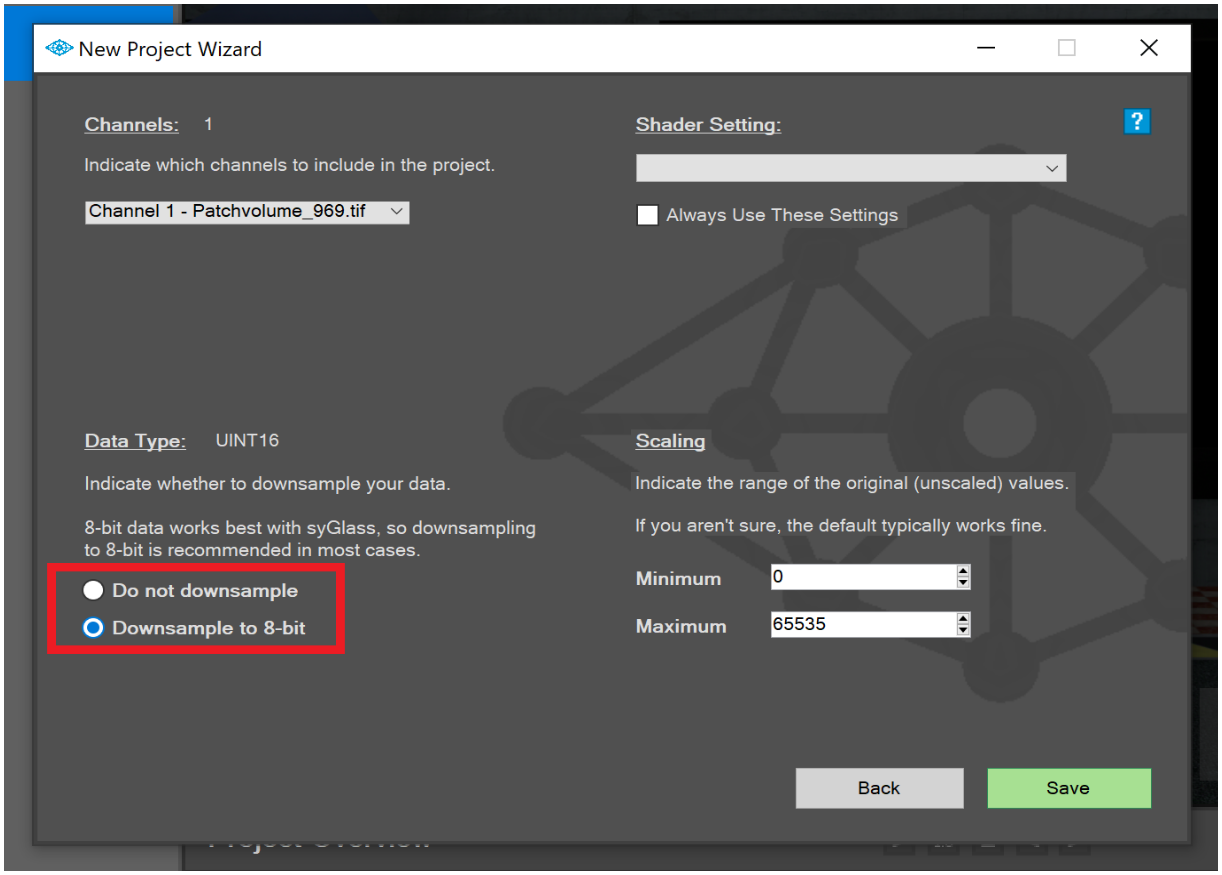

- When the import has finished, your syGlass data file will appear in the column on the left.
    - Double click on the file to open it or, alternatively, select the file and click on "Launch Project".
        - Files can be opened and closed using the VR-headset too.

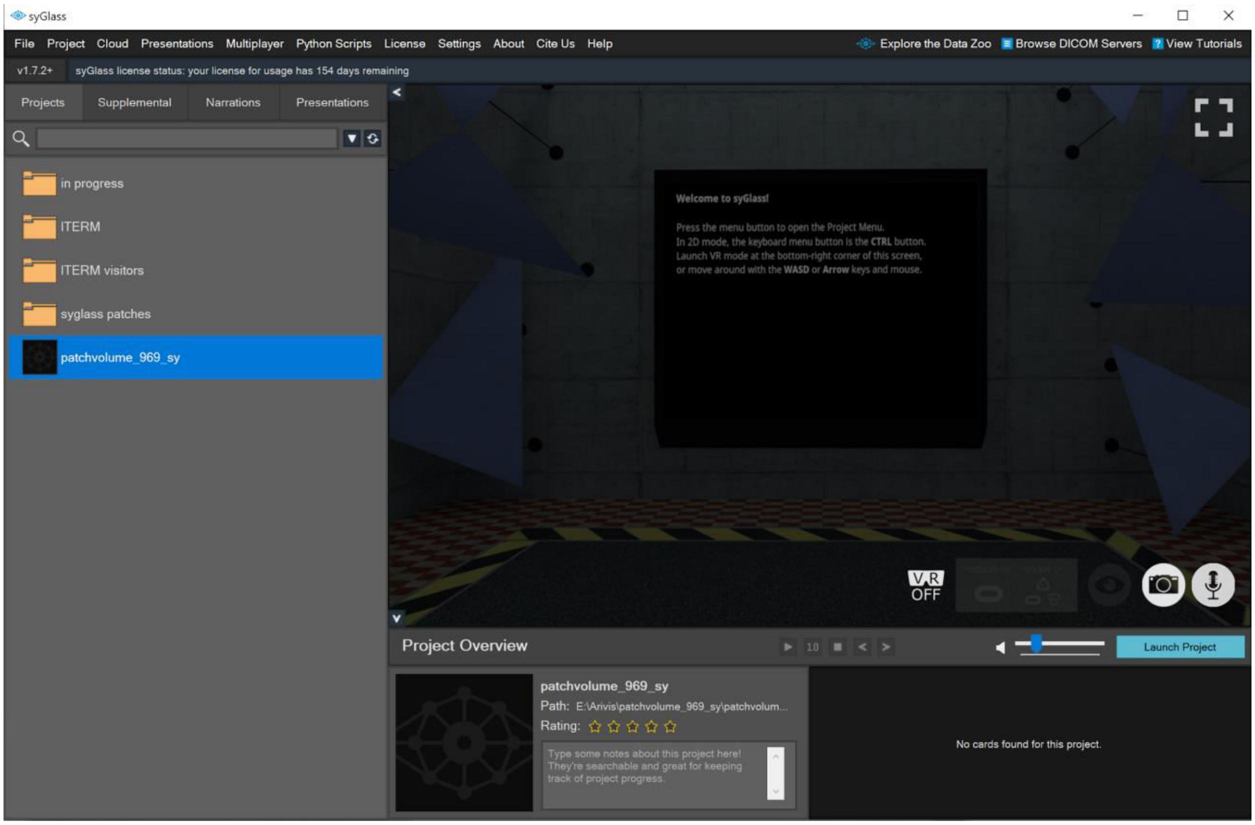

1.4.1. General instructions using VR-Headset and controllers in syGlass

Our description of the VR-Headset and controller usage are based on the Oculus Rift with Oculus Touch controllers, which we used for our annotations. However, when using other VR headsets, please note that controller functions can be slightly different.

- To activate VR, click on the "VR OFF" button on the lower right site of the screen. Depending on which VR system you are using, select either "Oculus VR" or "Steam VR".

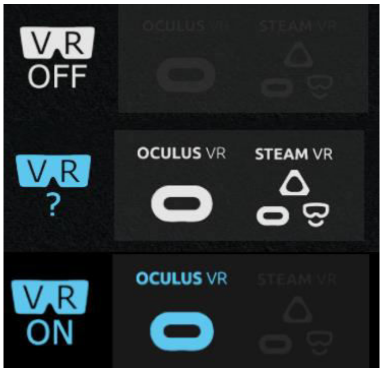

- Put on the VR-headset (eg. Oculus Rift) and use the handhold controllers to navigate through the software.
- Key assignments of the controls can be found in Table 2.
    - **B / Y** buttons opens/closes the menu.
    - The **Front trigger** on both controllers will be used to select, click and to use the tools (ex. moving 3D-file/ cut-planes / ROI-annotation.
    - The switch tool buttons (**A**/**X**/**Side trigger**) lets you change quickly between selected tools.
    - The Joystick can be used to select different actions of selected tools.

Table 2: Controller settings / key assignments

Key	Function
**Front trigger** (index finger)	Select / Use tool (ex. move, cut, annotate)
**Side trigger** (middle finger)	Switch tool / erase in ROI-tool
**A / X**	Switch tool
**B / Y**	Open / Close menu
**Joystick**	Rotate room / open tool options

.

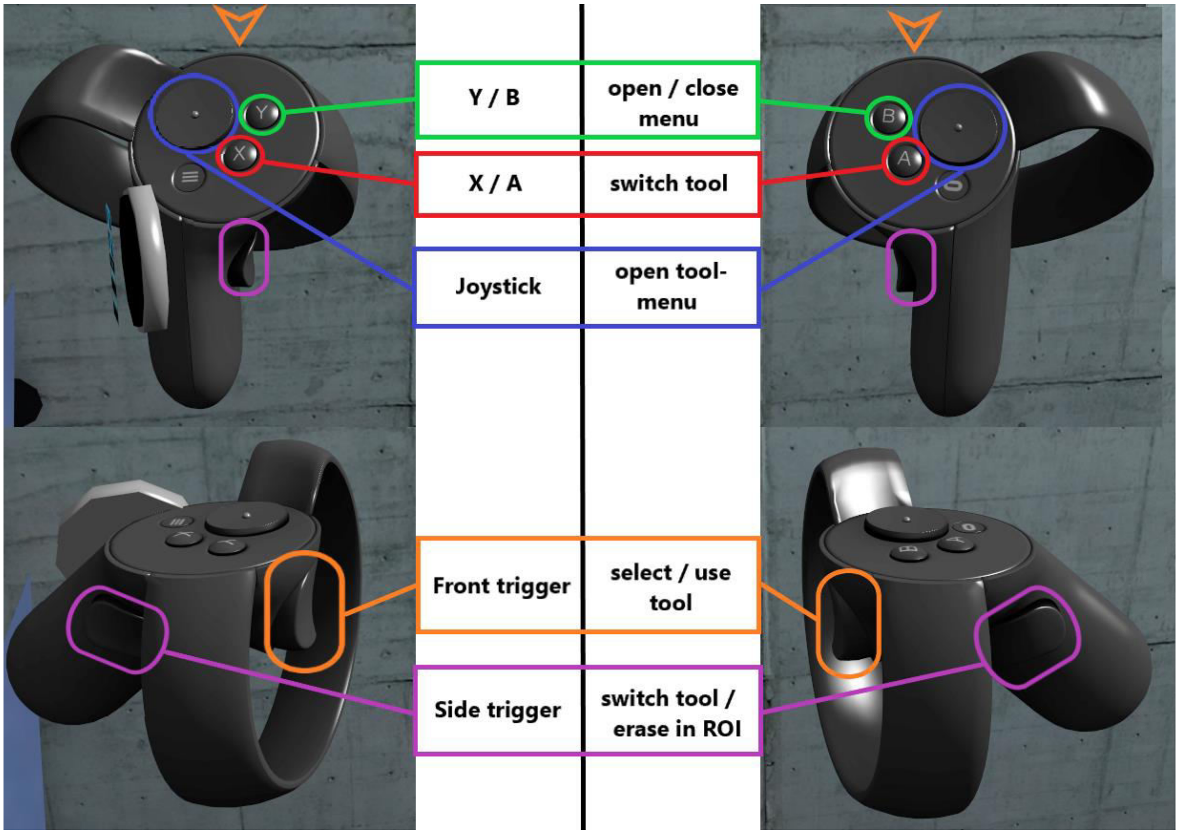

- The VR main menu window (can be opened by pressing **X**/**A** on your controller) shows all folder/files imported in syGlass.
  - Settings are displayed at top right in the main menu window.
  - All tools can be selected at the bottom of the main menu window.
  - On the right side of the main menu, there are specific tool menus (connected to active tools on controllers).
- Each controller can have two tools selected. This can be changed in the VR main menu anytime.
  - Controllers will be set on the Navigation- / Cutplane-Tool when entering the VR-room on first opening.

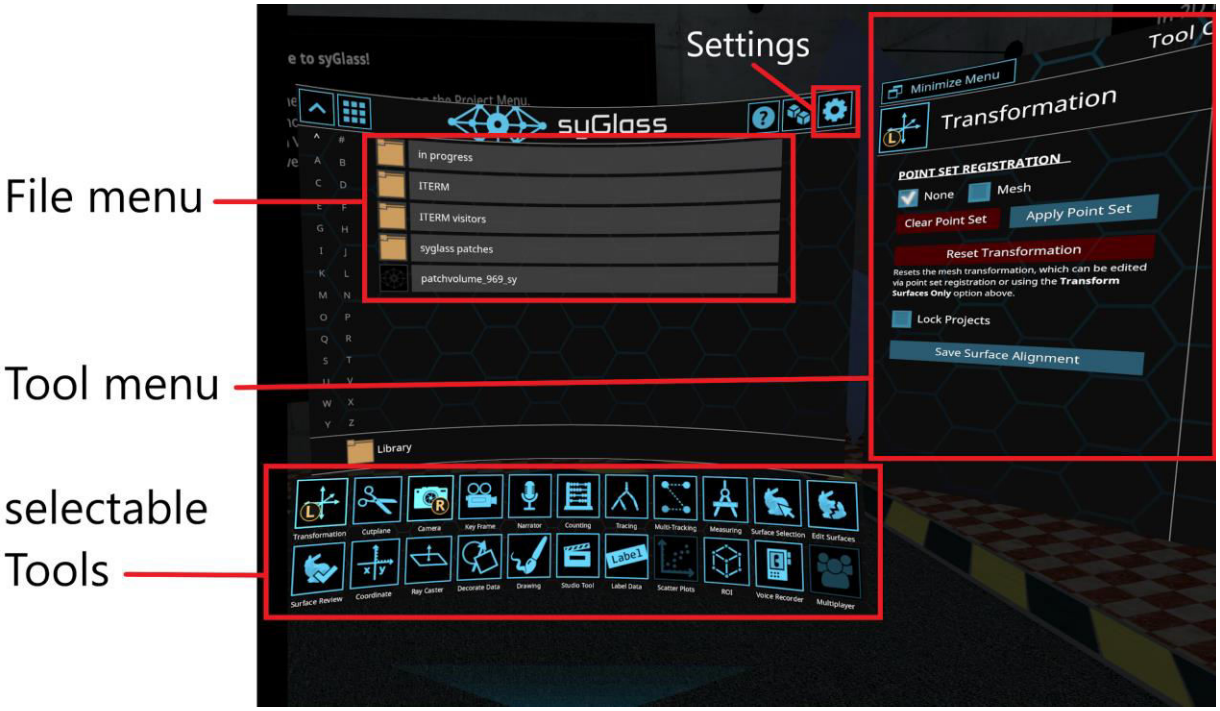
Settings
File menu
Tool menu
selectable
Tools
syGlass
in progress
ITERM
ITERM visitors
syglass patches
patchvolume_969_sy
Library
Minimize Menu
Transformation
POINT SET REGISTRATION
None
Mesh
Clear Point Set
Apply Point Set
Reset Transformation
Lock Projects
Save Surface Alignment
Transformation
Cutplane
Camera
Key Frame
Narrator
Counting
Tracing
Multi-Tracking
Measuring
Surface Selection
Edit Surfaces
Surface Review
Coordinate
Ray Caster
Decorate Data
Drawing
Studio Tool
Label Data
Scatter Plots
ROI
Voice Recorder
Multiplayer

1.4.2. Annotating data using syGlass

In this section, we describe the basic procedure of annotating data in VR on our data set. For this, we used the “ROI”-Tool. Additional tools that help navigating through the data and other purposes are available.

- Open the file you would like to annotate by using the **Front trigger** of the controller and click on the file you want to open.

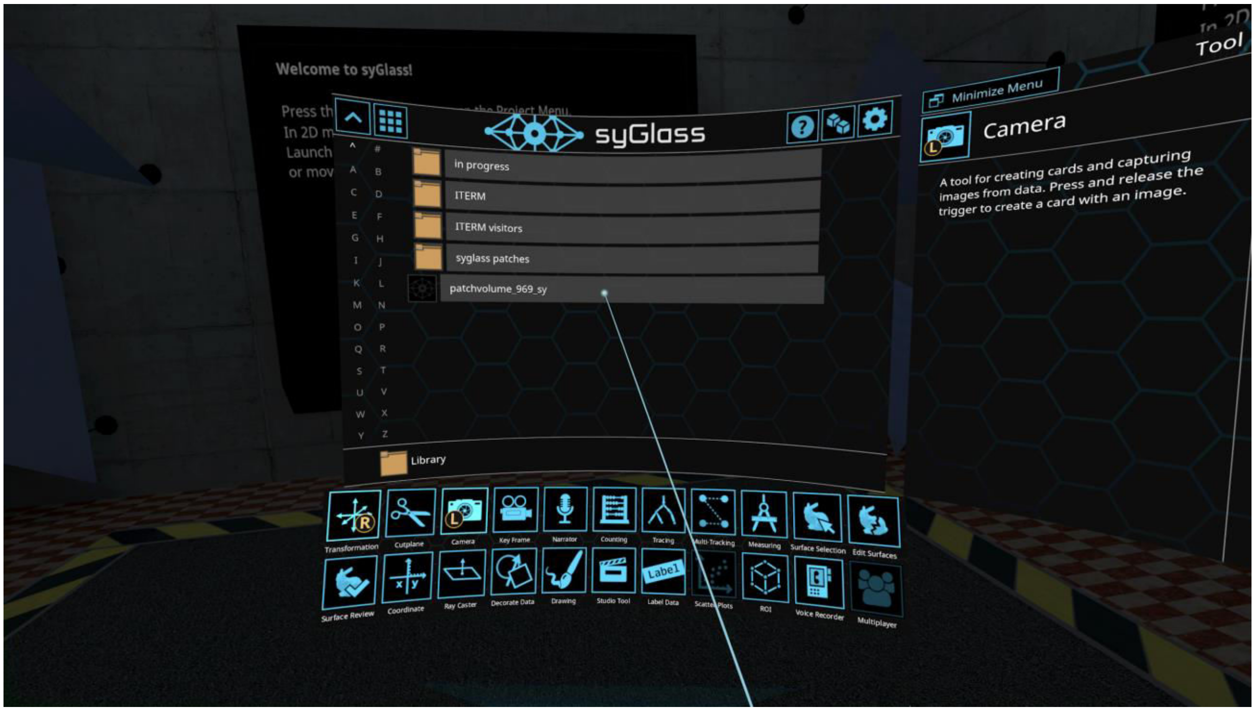

- The selected file will be rendered in VR. Below you see an example of our data set.

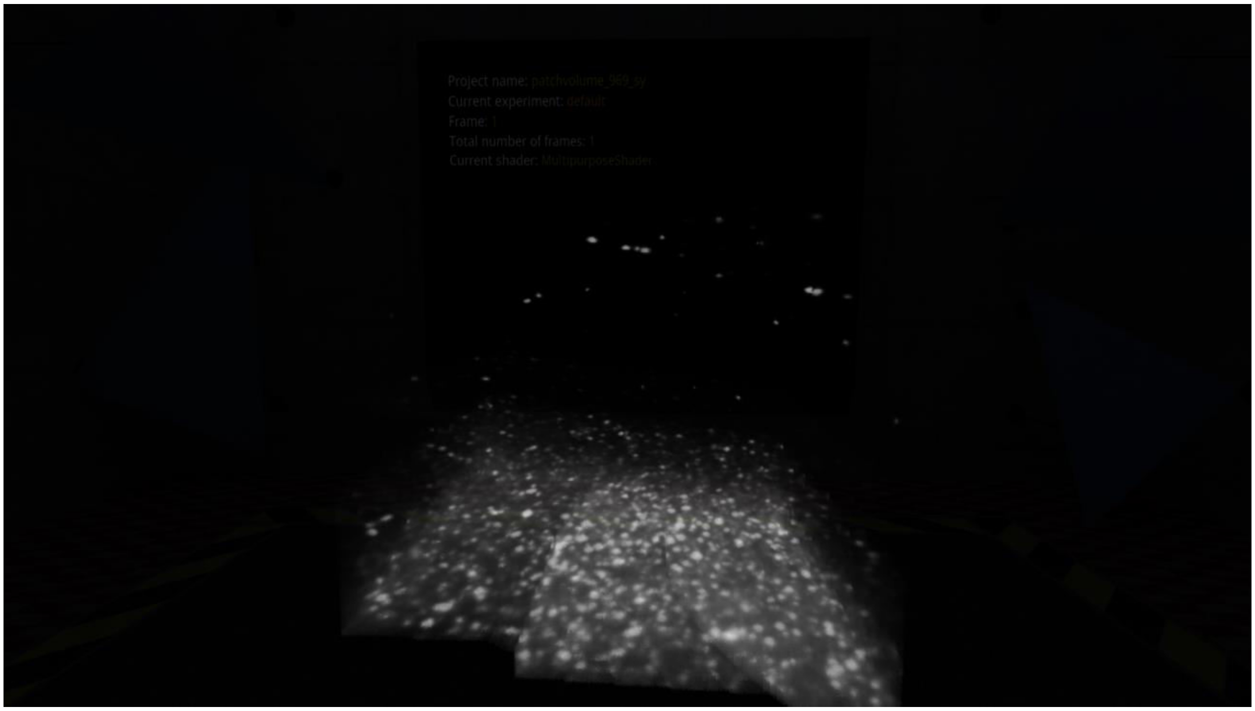

- In the menu, visual settings (e.g. contrast) can be adjusted:
    - Different maps are available (Gradient/Absorption/Max-Projection are the most useful for annotation).
        - Absorption-Map adds an opacity bar to visual settings.
- syGlass can save three different visual settings in VR.
- To change the visual settings, click and hold the **Front trigger** and move the light blue slider. Upon moving the slider, your data set will become visible to adjust the setting accordingly.

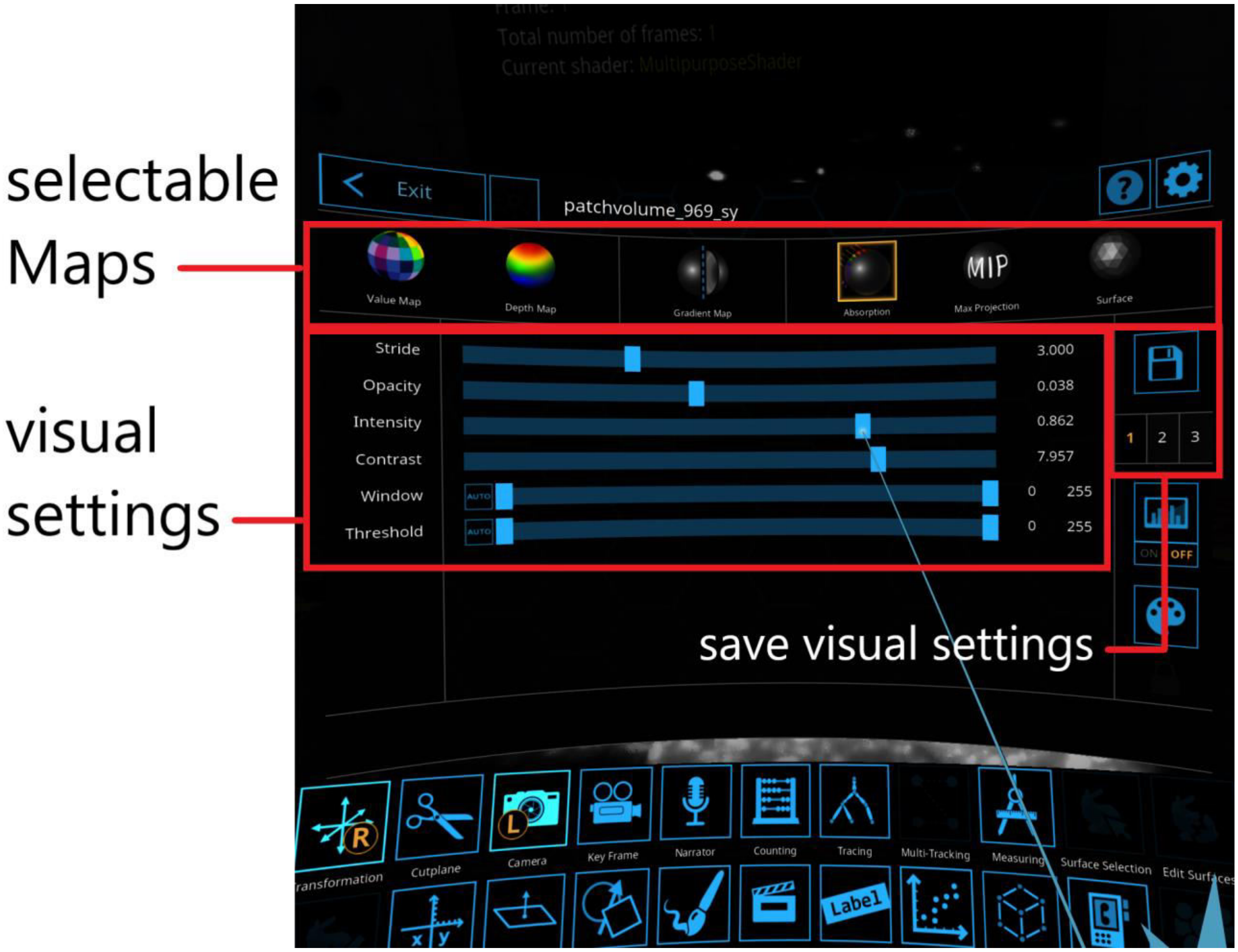
selectable Maps
visual settings
save visual settings
Exit
patchvolume_969_sy
Value Map
Depth Map
Gradient Map
Absorption
MIP
Max Projection
Surface
Stride
Opacity
Intensity
Contrast
Window
Threshold
3.000
0.038
0.862
7.957
0 255
0 255
1 2 3
ON OFF
Transformation
Cutplane
Camera
Key Frame
Narrator
Counting
Tracing
Multi-Tracking
Measuring
Surface Selection
Edit Surfaces
Label

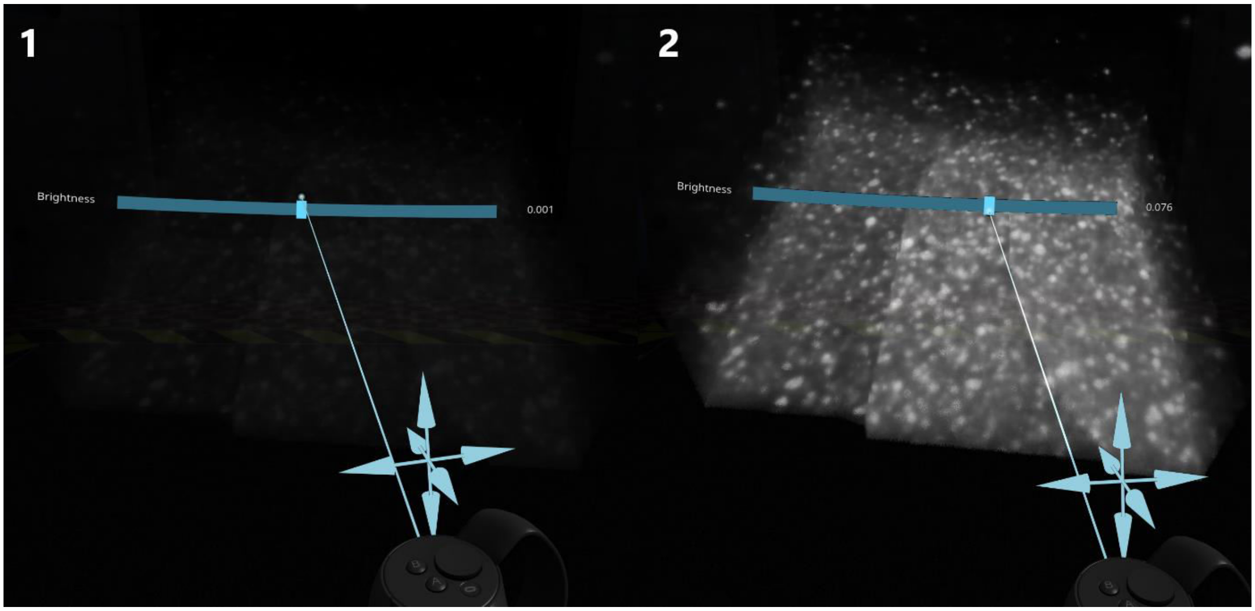
1
Brightness
0.001
2
Brightness
0.076

- Besides the “ROI”-Tool that we used for our annotation, additional tools are available in syGlass that help navigating through the data.

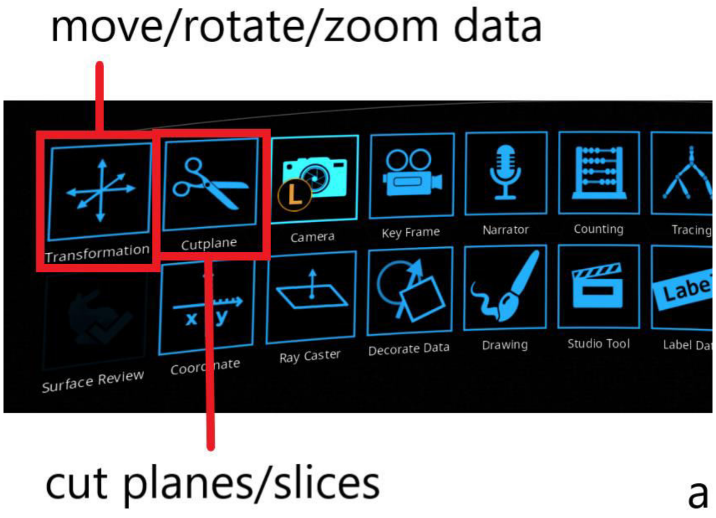

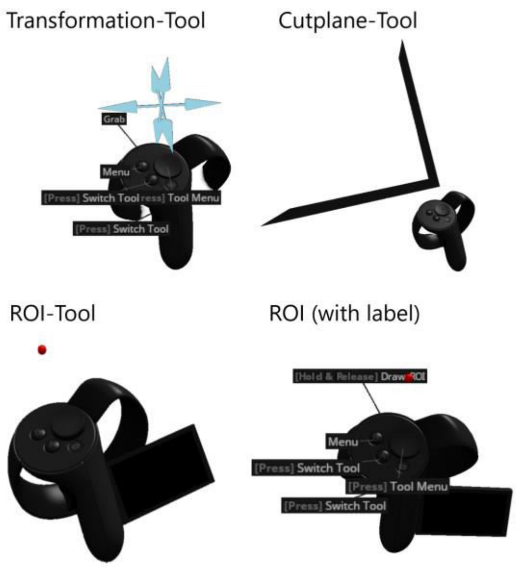

- Transformation-Tool
  - Use to move, rotate, zoom your 3D data
- Cutplane-Tool
  - Cut multiple planes or cut slices of different thickness
- ROI-Tool
  - Mark/annotate your data / signal

1.4.2.1. SyGlass ROI-Tool

- To annotate data in VR, select the ROI-Tool in the tool menu.
- The data volume that should be included in the annotation needs to be selected by first drawing a ROI box over it. Therefore, draw/move the orange ROI-box in the 3D-space over either a specific region of interest of your data set or the whole volume.
- Press the **Joystick** to confirm the ROI by moving controller into “Save ROI” (sphere gets blue) and click the **Front trigger**.
- The orange box will become green and the ROI-Tool is ready for annotation. Now, a magenta- colored sphere will appear on top of your controller that can be used to annotate your data.

- The sphere works similar to a brush at which everything inside of it will be annotated.

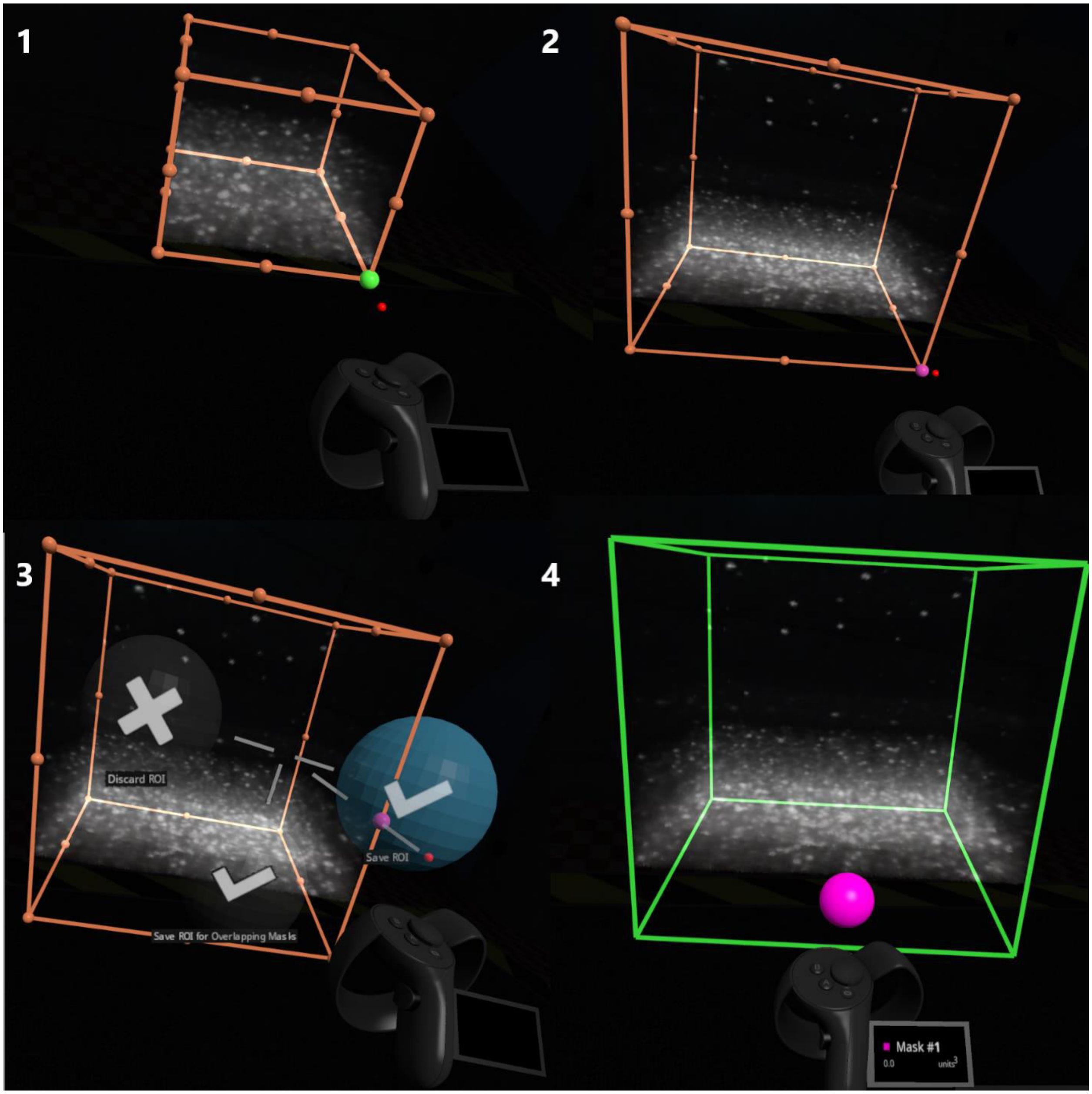

- To annotate, click or hold the **Front trigger** and move the sphere through the object of interest.
  - The size of sphere can be changed by rotating the **Joystick** on the controller with the ROI-Tool active.
  - The mask (annotation color/value) can be changed by clicking on the **Joystick** and selecting “Previous / Next Mask”.

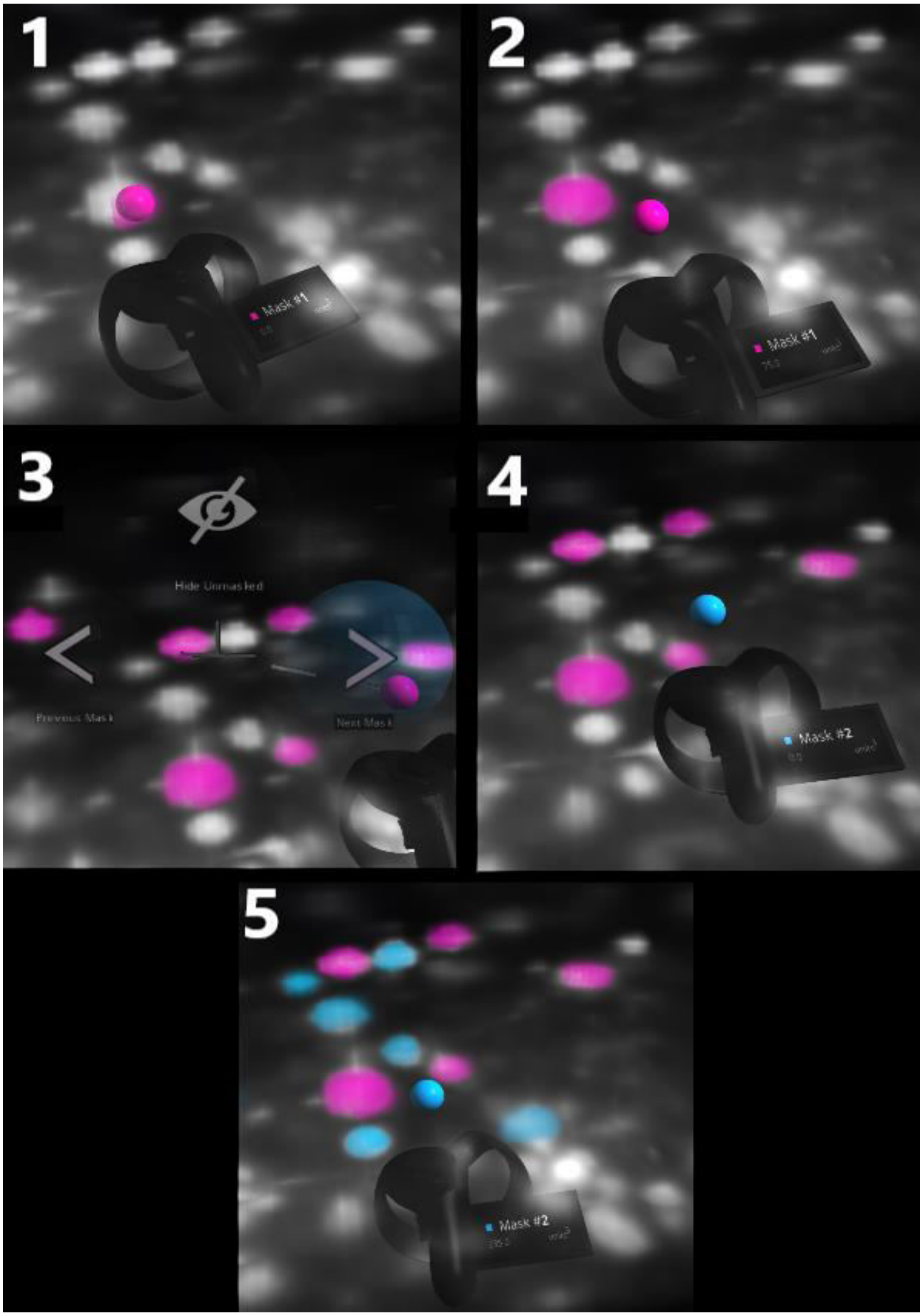

- To delete an annotation, hold the **Side trigger** (the sphere becomes white in color) and use the **Front trigger**.

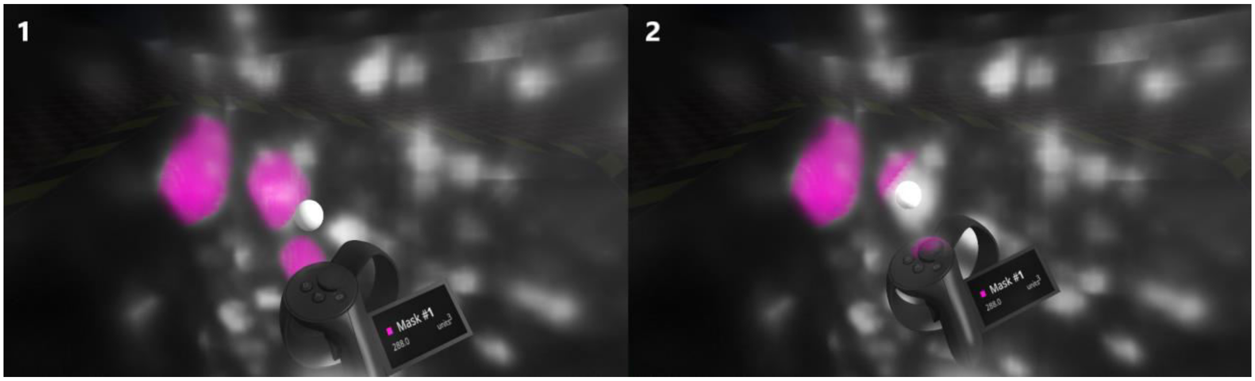

- Everything annotated will be saved automatically when you exit the file.
- You can adjust a threshold to remove background signal.
    - If a threshold is selected, anything below the selected threshold will not be annotated by ROI-tool.

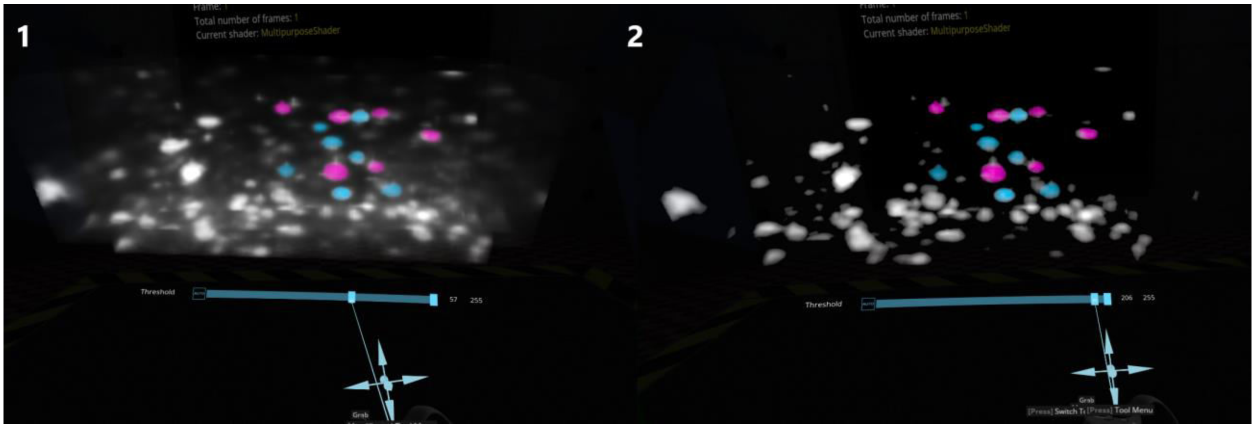

- You also can trim your mask/annotation via the tool menu selection “trim current mask”.
    - Here, set a threshold that removes the part of annotation you would like to exclude and click and hold on trim mask.
    - Every mask / color can be trimmed individually.

1.4.2.2. High resolution ROIs in Syglass

The high resolution ROI allows you to mark smaller rendered sub blocks/pixels for more accurate annotations if needed.

- For rendering ROIs in highest resolution, press the **Joystick** before drawing the ROI and select the sphere on the bottom.

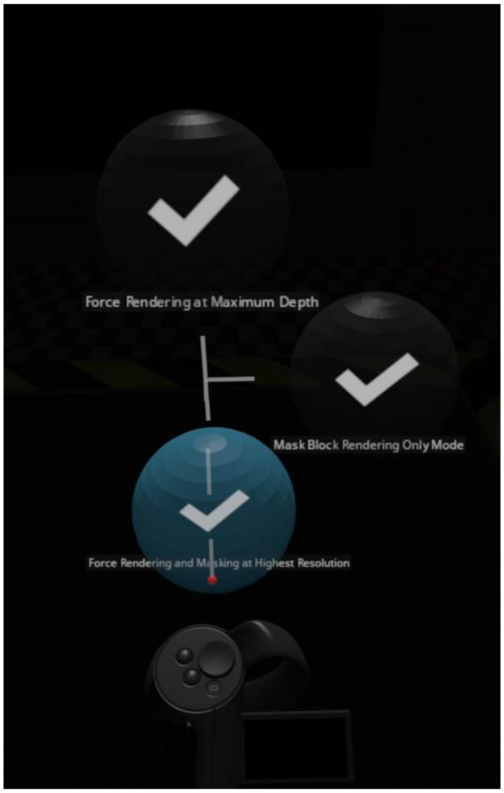

- It creates a high resolution ROI based on your computers capabilities.
  - ROIs can be moved if it doesn't cover the whole volume (resize in Tool-menu).
  - It is only possible to create one high resolution ROI per file.

1.4.2.3. ROI tool menu – managing ROIs

- If you created multiple ROIs in your data set, they will be listed in ROI-tool menu (right next to visual settings menu).
- In ROI tool menu you can select/ merge/ delete/ dilate/ trim already existing masks.
- A high resolution ROI will not appear in ROI list, as it is only accessible by the **Joystick** and selecting the sphere.

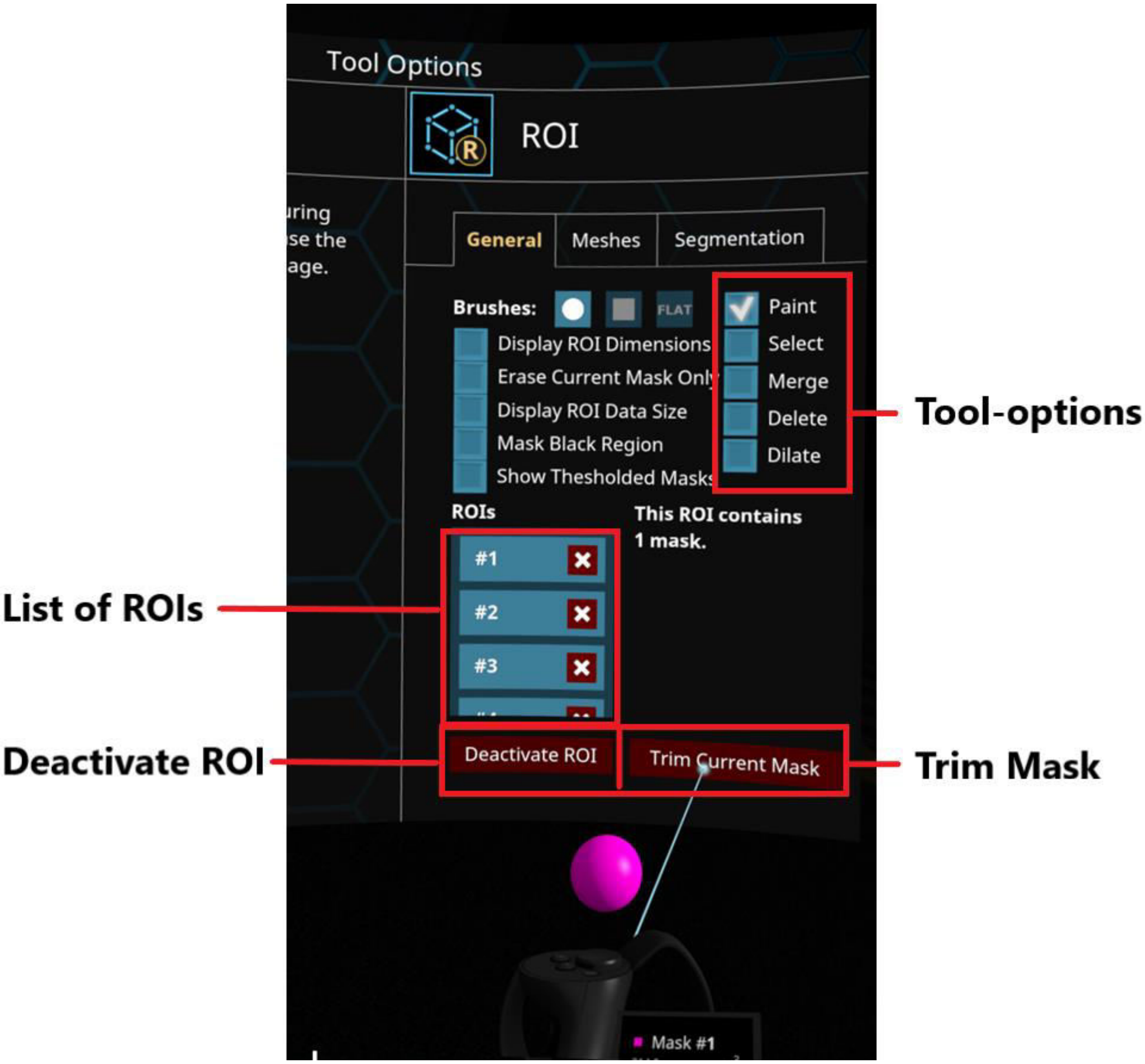

1.4.3. Export annotations in syGlass

- To export annotations/masks made in syGlass, open the file in syGlass and click on "Project" → "ROIs".
- Select ROI/Channels for export in the "ROIs"-window and click on "Export Data" for standard ROIs (non-high resolution ROIs).
  - For high resolution annotations, select "Export Mask Octree".
- Select the saving location.
- The raw data and the mask will be exported in Tiff-slices.

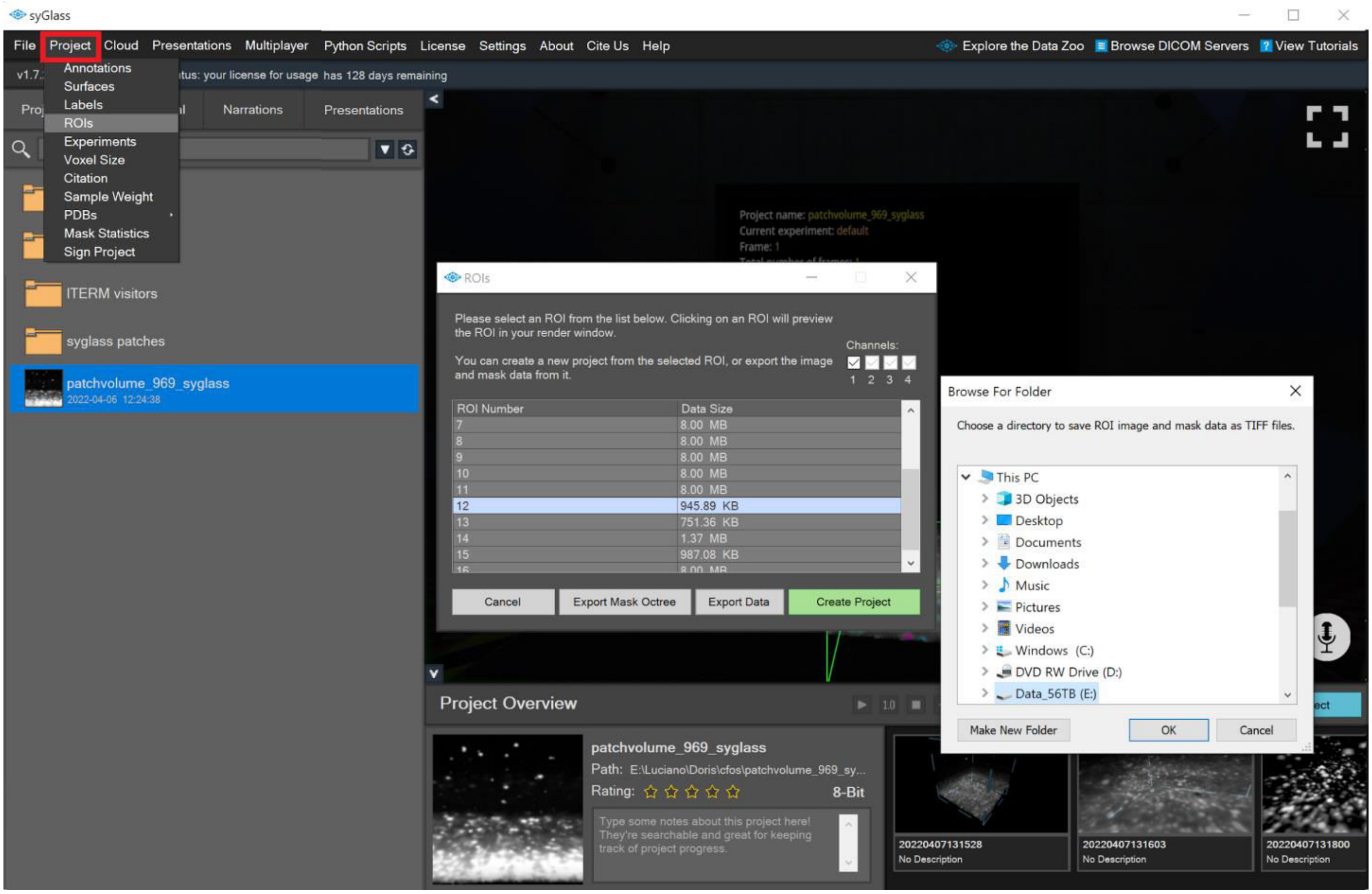

1.4.4. Helpful tips for annotation / visualization in syGlass

- Try to change your perspective while working with your 3D data. This helps a lot to identify your signal, false negatives / positives or artifacts.
- Instead of looking from the outside, try to dive into the data set.
- Try out different visual settings to identify less obvious annotation objects.
- With the ROI-tool, press the joystick to hide unmarked or marked data. This will help you to judge if you annotated too little or too much of your signal. It will also allow you to discover low intensity signal.

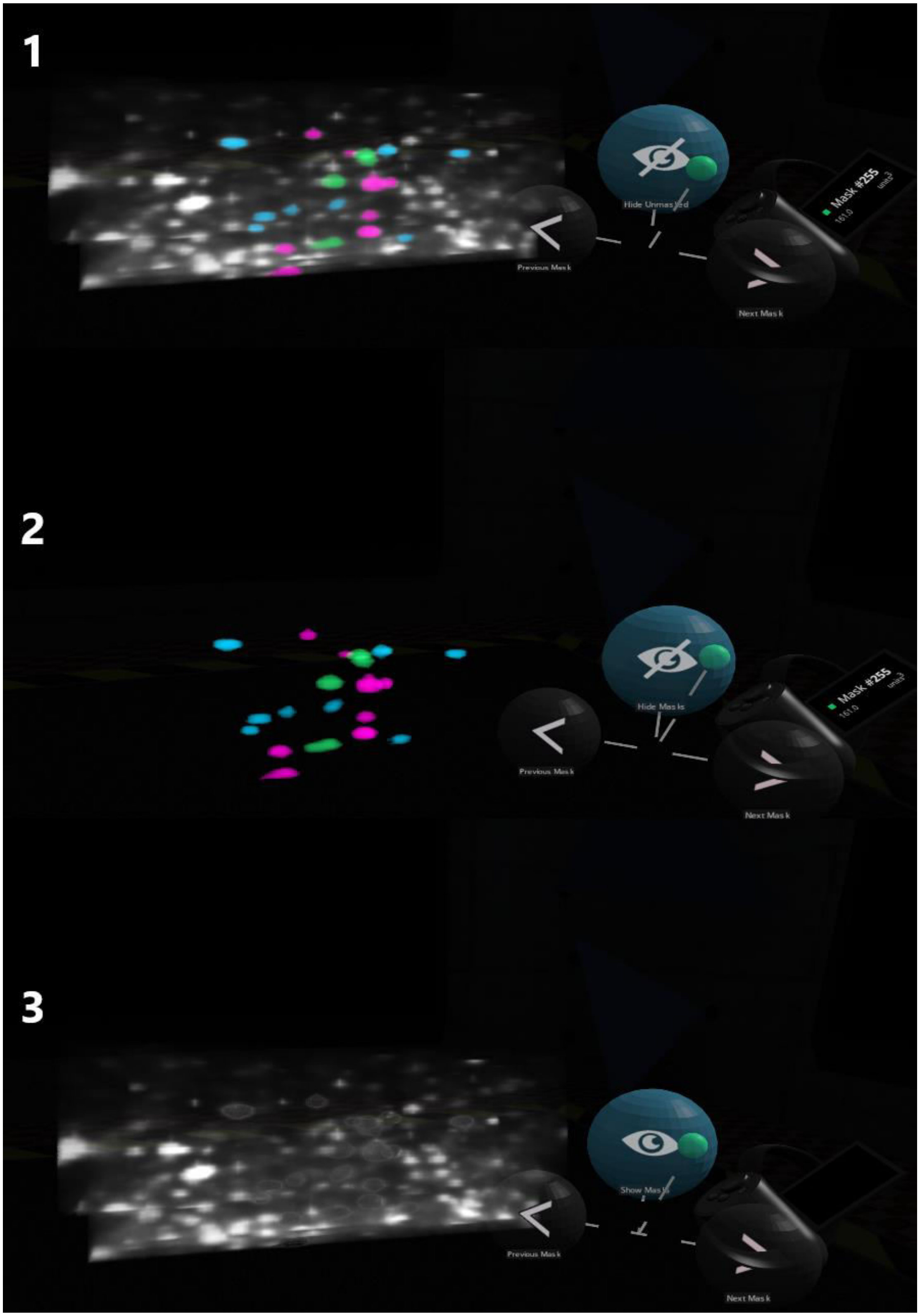

- Use the plane tool, to remove areas that hinder your view.

- You can turn off the virtual room in VR-settings under rendering. Thereby, you will not have any background interfering with your view.

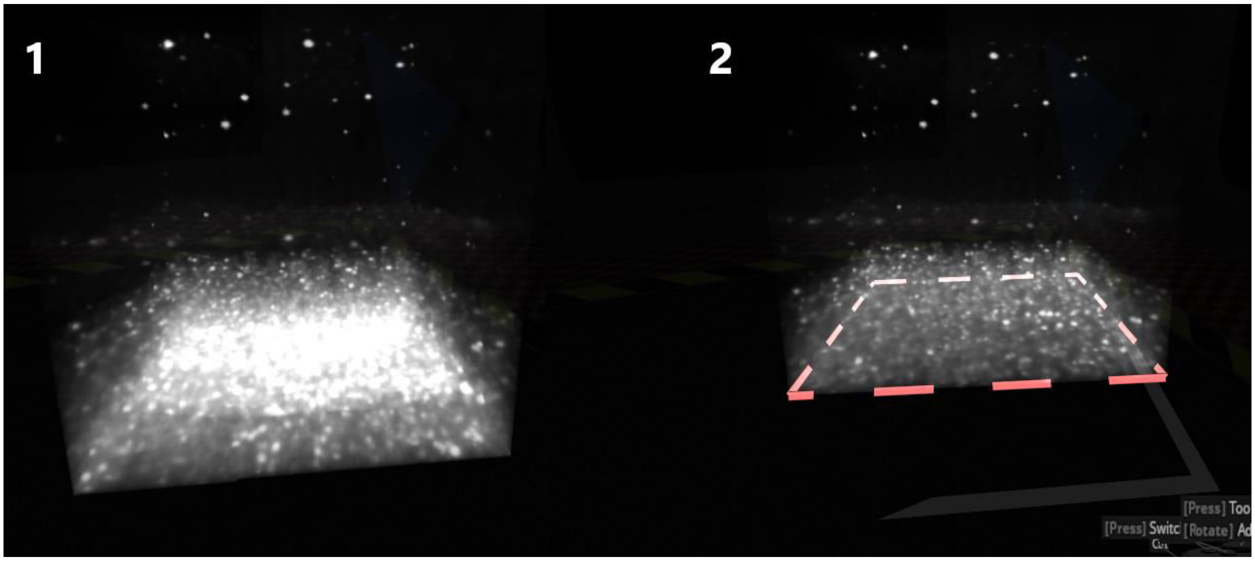

2. Step-by-Step guide for using DELiVR

2.1 System requirements

- Following system requirements are necessary to run DELiVR:
  - CPU: Desktop-class CPU with >4 cores and hardware hypervisor capabilities.
  - RAM: **64 GB, or 32GB with >50GB swap space.**
  - Disk space: **Dataset size x 5** (NVMe SSDs preferred. Example: For a dataset with 50GB 16-bit tiff stacks, 250 GB free space are required).
  - Graphics card: Nvidia CUDA 11.7 capable or higher, **8GB VRAM or higher required.** We tested and confirmed working on RTX 2070 Super with 8GB VRAM.
  - Operating System: Windows 10 or higher, Linux: Ubuntu 20.04 or higher.
    - **Requires administrator privileges to run.**
  - Software: Docker Desktop (windows), docker and nvidia-docker-tools (Linux). Note that nvidia-docker-tools is only required for Linux. If you run the docker under windows and get a "driver not found, install nvidia-docker-tools" error message, make sure that docker can communicate with the graphics card (i.e. by adding the *–gpus=all* flag to the *docker run* command).

2.2. Data preparation

To run DELiVR, the pipeline necessitates a folder structure where each folder houses a single brain scan in the form of TIFF files, with one TIFF file corresponding to each z-slice. We scanned in transversal orientation (viewed from dorsal side, z-axis is dorsal -> ventral). All TIFFs must be 16 bit with values between 0 and 2^{16}.

2.3. Installation

2.3.1 Installing DELiVR Docker image (required for Fiji plugin)

Docker is a software platform that allows to bundle and distribute applications, along with their required components, in a uniform container format known as Docker containers. These containers serve as self-contained environments encompassing everything essential for running an application, including its code, runtime, libraries, and system tools. In order to run DELiVR end-end, we packaged all code to run the pipeline in a single docker container. To run the DELiVR docker image, it is therefore required to install the Docker Desktop Application for Windows or when using Linux, docker and nvidia-docker-tools are needed.

- Install docker.
    - Windows: download and install Docker Desktop: here
        - To be able to install Docker Desktop, it is necessary to have administrator rights on the computer.

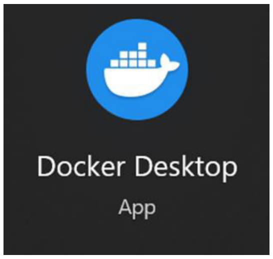

    - Linux:
        - Install nvidia-docker-toolkit: following these instructions .
        - Use our install script (with admin rights): Script from github
- Download our DELiVR Docker image from https://www.discotechnologies.org/DELiVR/ .
- Install our DELiVR docker image.
    - In windows:
        - Open the windows powershell

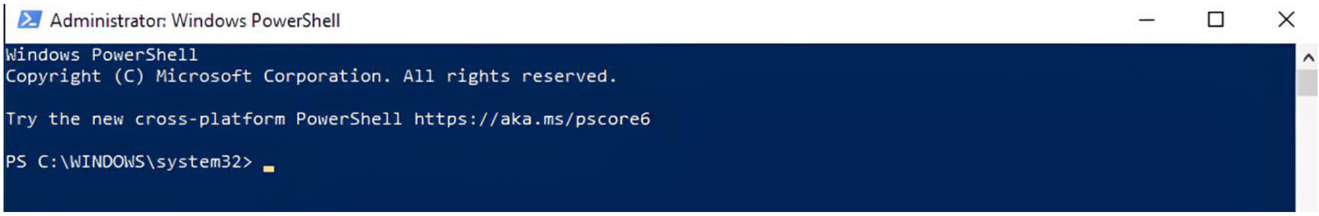

        - Change the directory to the folder where the DELiVR docker image is located by using the command
            - cd [insert path of the folder with the location of the DELiVR docker image]
        - Install the docker via following command (from the folder where the docker container is located):
            - docker image load -i .\delivr_*.tar.gz
            - Installation of the DELiVR docker image can take up to 30 minutes

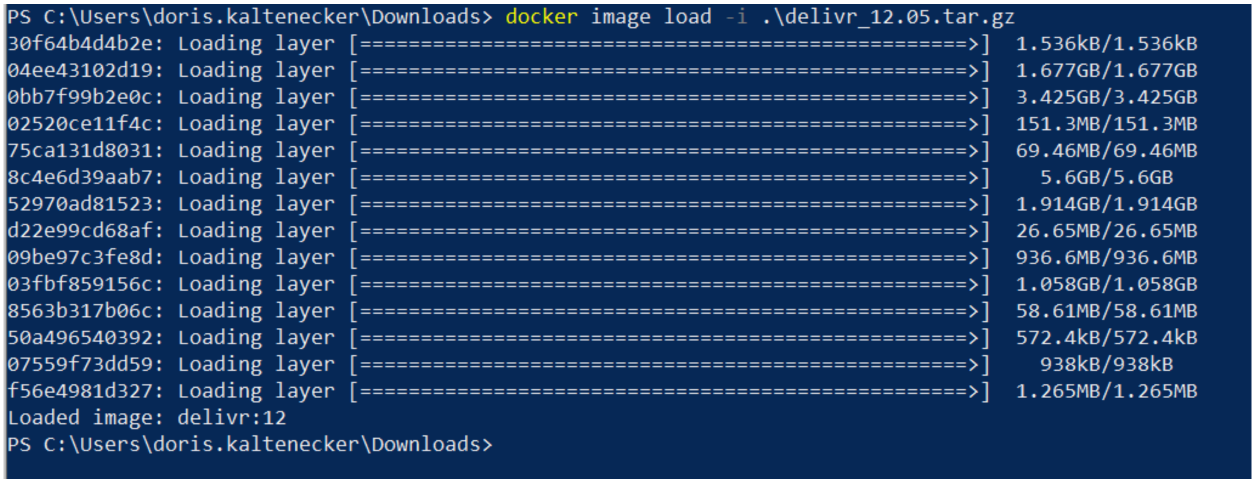

- After successful installation, the DELiVR docker image "delivr" should appear in the docker desktop application under "images".

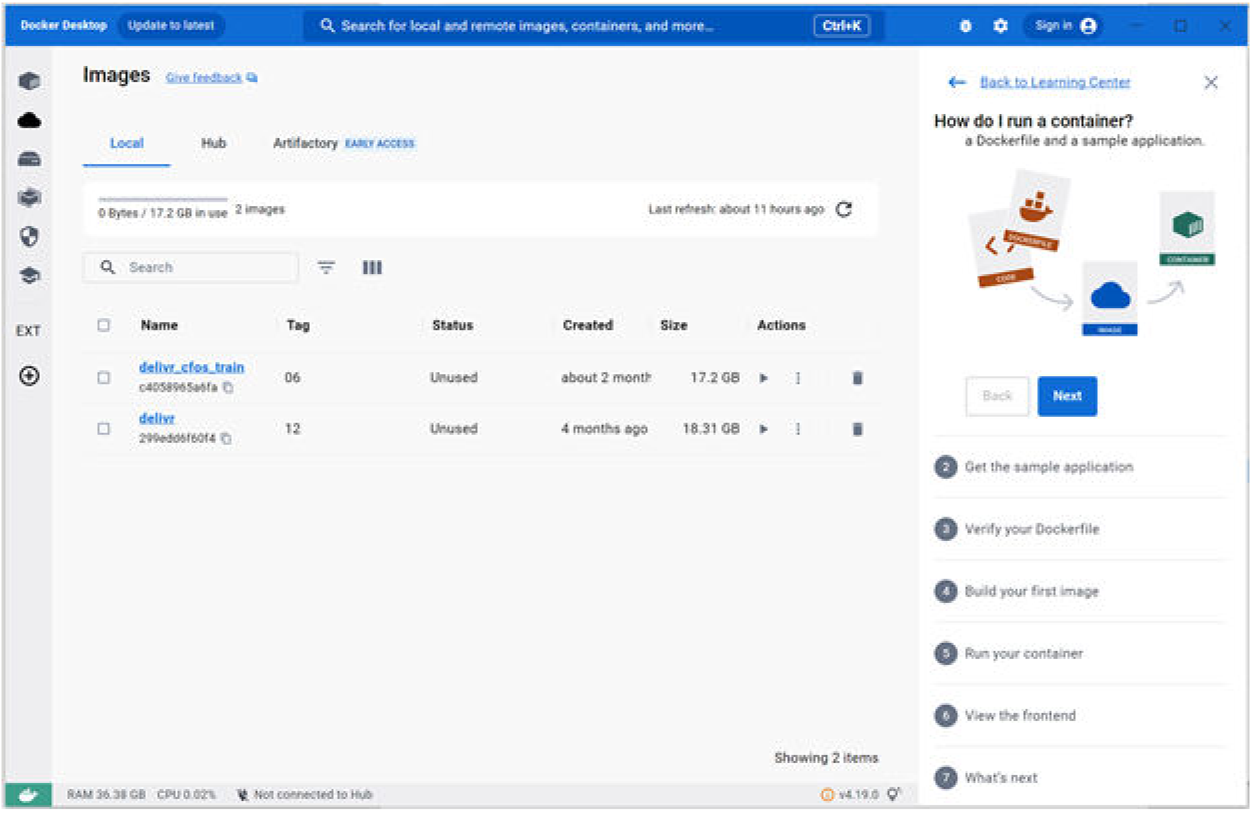

- In Linux:
  - To install manually, open a terminal and type:
    - sudo docker image load -i .\delivr_*.tar.gz

- Verify installation and communication with the Nvidia CUDA drivers (optional but recommended) by typing:

- Windows: Powershell
  - docker run -it –rm --gpus=all --rm nvidia/cuda:11.8.0-base-ubuntu22.04 nvidia-smi
- Linux: Terminal:
  - docker run -it –rm --runtime=nvidia --rm nvidia/cuda:11.8.0-base-ubuntu22.04 nvidia-smi

2.3.2 Fiji Plugin

To run the DELiVR pipeline via the Fiji Plugin, docker as well as our docker image are required (see above). The plugin can be downloaded here: https://www.discotechnologies.org/DELiVR/ .

- For docker installation and docker image installation please see 2.3.1.
- To install the DELiVR Fiji plugin, the plugin file simply has to be moved to the **plugins** folder of Fiji (for example **/fiji-linux64/Fiji.app/plugins/**).
- When the DELiVR plugin is in the plugins folder in Fiji, DELiVR will appear in Fiji after the next restart.

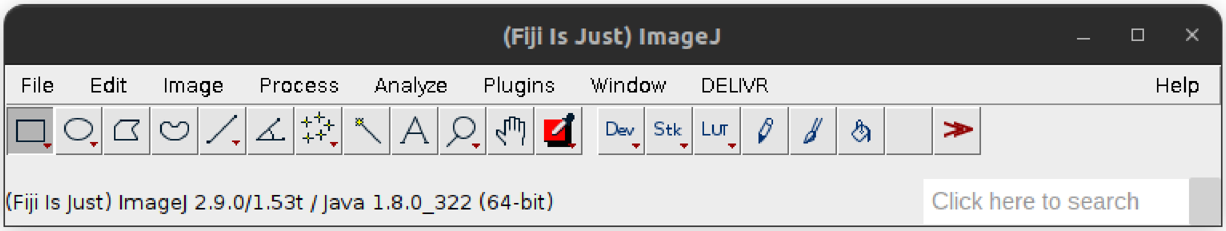

2.4 Running DELiVR end-to-end

2.4.1 The config file

The config file contains all parameters needed to run DELiVR. It will be generated by the DELiVR plugin after the “Start” button in the plugin has been pressed. The config file will then be passed on to the docker container. When using the docker container without the plugin, you can adapt this template: https://github.com/erturklab/delivr_cfos/blob/main/config.json. All fields describing paths should remain unchanged. The parameters are the following:

**Parameter name**	**Description**
raw_location	Path of the input data, a folder containing folders of TIFFs (one folder for each brain)
output_location	Path where the output of the pipeline should

	be stored
mask_detection / ilastik_location	Path of Ilastik runnable
mask_detection / ilastik_model	Path to the trained Ilastik model for ventricle detection
mask_detection / teraconverter_location	Path to TeraConverter
mask_detection / output_location	Path where the output of the mask detection is stored
mask_detection / downsample_steps / original_um_***x/y/z***	Original scanning resolution along axis ***x/y/z***
mask_detection / downsample_steps / downsample_um_***x/y/z***	Target resolution of the downsampled atlas (in our case:25µm/px) in axis ***x/y/z***
mask_detection / mask_with_Ilastik	True: Use Ilastik (with /delivr/models/random_forest_weights.tar) to mask out ventricles.<br>False: Skip Ilastik for ventricle masking, just set everything below ***simple_threshold_value*** to zero. Useful for hemispheres or samples other than mouse brains.
mask_detection / simple_threshold_value	If not masking with Ilastik, set everything below this value to zero. Default: 250
blob_detection / input_location	Path to masked brains as an input for the cell detection; should be the same as mask_detection / output_location
blob_detection / model_location	Path to the trained U-Net model
blob_detection / output_location	Path where the output of the cell detection is stored
blob_detection / window_dimensions	The width of the sliding window analyzer's windows during inference. This should match the window size used in training for optimal segmentation performance (default: 96x96x64).
postprocessing / input_location	Path to binary images containing segmented cells; should be the same as blob_detection / output_location
postprocessing / output_location	Path where the output of the postprocessing is stored

postprocessing / min_size	Minimum size in voxel for the postprocessing; cells smaller than this will be filtered out; -1 indicates no lower bounds
postprocessing / max_size	Maximum size in voxel for the postprocessing; cells larger than this will be filtered out; -1 indicates no upper bounds
atlas_alignment / input_location	Path to csvs containing the segmented cells and their location in 3D, should be the same as postprocessing / output_location
atlas_alignment / output_location	Path to where the output of the atlas alignment is stored
atlas_alignment / mBrainAligner_location	Path to the mBrainAligner executable
atlas_alignment / collection_folder	Collects all atlas aligned cell coordinate tables
atlas_alignment / parallel_processing	Flag for enabling parallel processing in atlas alignment. Default: ***true***
region_assignment / input_location	Path to the input for the region assignment; should be the same as atlas_alignment / collection_folder
region_assignment / CCF3_atlasfile	Path to the CCF3 atlas file, the final heatmaps should map onto this.
region_assignment / CCF3_ontology	Path to the CCF3 ontology table file
region_assignment / output_location	Path to where the output of the region assignment is stored
visualization / input_csv_location	Path to region assignment csvs for the visualization step; should be the same as region_assignment folder
visualization / input_size_location	Path to size csvs for the visualization step; should be the same as postprocessing / output_location
visualization / input_prediction_location	Path to the image files of the binary segmentation; should be the same as blob_detection / output_location
visualization / cache_location	Path to folder where large intermediate results are cached
visualization / output_location	Path to where the final visualization images

	are stored
visualization / region_id_rgb	Create tiff output of cell segmentations, color-coded by Allen Brain Atlas RGB values. Default ***true***
visualization / region_id_grayvalues	Create tiff output of cell segmentations, colored by the region_id for easy thresholding. Default ***false***
visualization / no_atlas_depthmap	Create tiff output of cell segmentations, color-coded by depth (i.e. distance to the nearest masked voxel). Useful for samples that are not mouse brains and therefore have no atlas. Default: ***false***

Furthermore a range of Flags exist:

Flag	Description
ABSPATHS	True if the paths in the config are absolute paths, false if they are nested in the output path. Default: ***false***
TEST_TIME_AUGMENTATION	Performs test time augmentation if true, otherwise only does one inference for each brain; TTA improves segmentation results but takes considerably longer (ca 10x). Default ***true***
MASK_DOWNSAMPLE	Generate downsampled mini-stacks (important for atlas registration) and mask (either ventricle masking or thresholding), plus prepare the .npy file for blob_detection. Only deactivate if re-running the pipeline. Default ***true***
BLOB_DETECTION	Segment the image stack with the sliding_window_inferer. Default ***true***
POSTPROCESSING	Run connected-component analysis on the blob_detection results. Default ***true***
ATLAS_ALIGNMENT	Use the downsampled stacks from the mask_downsample step to register the brain to the CCF3 atlas with mBrainAligner. Default ***true***

REGION_ASSIGNMENT	Check in which brain region the atlas-mapped cell coordinates are located. Aggregate this information into a per-cell table, a per-region table, and a heatmap. Default ***true***
VISUALIZATION	Generate one or more visualizations in image space: Take the blobs and color-code them either by CCF3 atlas region color (RGB) or atlas region_id (grayscale). Default ***true***
SAVE_MASK_OUTPUT	Save the masked raw files separately as tiff stack, in addition to the (mandatory) .npy file. Default: ***true***
SAVE_NETWORK_OUTPUT	After inference, keep the .npy with the inference_output. Useful in case you want to inspect the network output before binarization. Default ***true***
SAVE_ACTIVATED_OUTPUT	Save the activated network output of the inference step. Useful as a diagnostic to troubleshoot inference problems. Default ***false***
SAVE_POSTPROCESSING_OUTPUT	Save the output of the connected-component analysis as a pickle file. Useful for diagnostics, or if you anticipate running the visualization several times (not having to run its own connected component analysis saves time). Default ***true***
SAVE_ATLAS_OUTPUT	Save the output of the atlas registration step (i.e. what's in the 04_atlas_alignment/output folder). Useful if you want to inspect the .v3draw output of mBrainAligner with Vaa3D. Default: ***true***

The rest of the flags describe which operations to perform and whether or not to save the output of each operation.

2.4.2 Running DELiVR from Docker (optional)

Docker is essentially a stripped-down Linux environment that is separate from the main computer. This means that everything required to run DELiVR comes pre-packaged in the docker container. Using the docker container, DELiVR can be used with minimal setup on any computer (or high performance clusters / cloud node) that fulfills the system requirements (Section 2.1). The docker container is also what the Fiji plugin uses in the background to execute all computations.

If you want to change parameters, you can do so as follows:

1) adapt the following template: https://github.com/erturklab/delivr_cfos/blob/main/config.json
2) Place the resulting file in a folder the container will be able to see, e.g. the OUTPUT_PATH
3) Add **/data/output/config.json** to the command below

Start the DELiVR container as follows:

- To start the DELiVR docker on Linux run the command:

```
sudo docker run --rm -i --runtime=nvidia -v RAW_PATH:/data/raw/ -v
OUTPUT_PATH:/data/output delivr:12 python3 __main__.py
```

- For Windows, the command is the following in an administrator Powershell:

```
powershell.exe docker run --rm -i --gpus=all -v RAW_PATH:/data/raw/ -v
OUTPUT_PATH:/data/output delivr:12 python3 __main__.py
```

RAW_PATH and OUTPUT_PATH are the respective paths to the folder of brain scans as well as the folder where the output will be stored. Adapt both to your specific folders before running.

2.4.3 Running DELiVR using the Fiji-Plugin (preferred)

- After successful installation of the docker container (Section 2.3.1) and the Fiji plugin (2.3.3), open Fiji and open the docker desktop application (windows) .
    - The DELiVR plugin should show up in the top bar of Fiji under **DELIVR**.

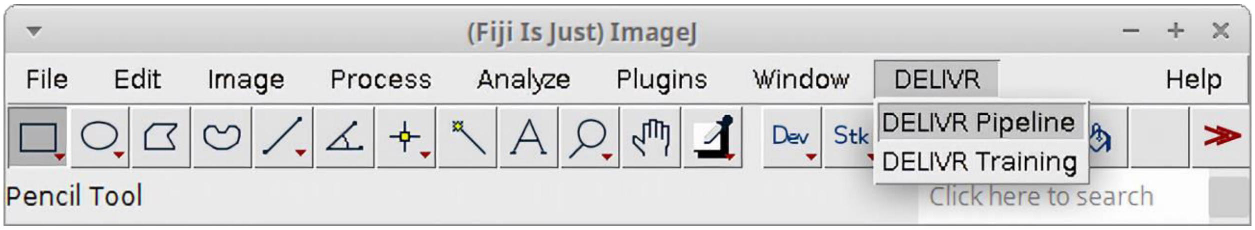

- Open the plugin by clicking on “DELiVR” in Fiji, and choose “DELIVR pipeline”.
    - The “DELIVR Pipeline”-window will appear.
- The DELIVR Pipeline”-window reveals two text inputs.
    - “Raw Data location“ is the directory of the microscope scans that should be analyzed (a path to a folder containing only subfolders of TIFF images of different brain scans).

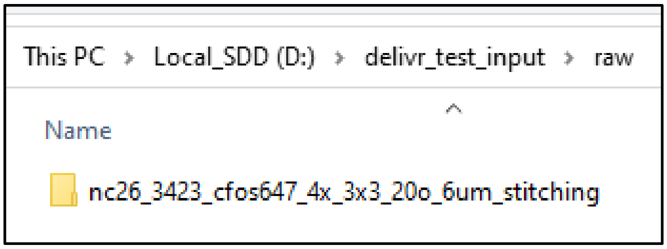

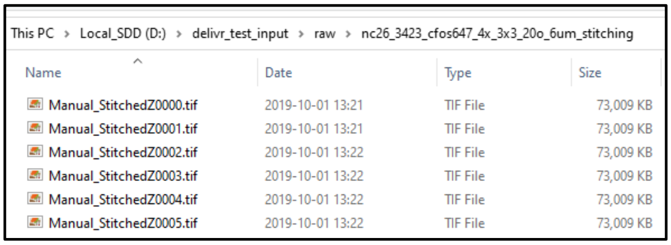

    - “Output location” is the directory where the outputs should be saved. If multiple subfolders with different brain scans are located in the “Raw Data location”, multiple subfolders with the result files will be generated in the “Output location”.

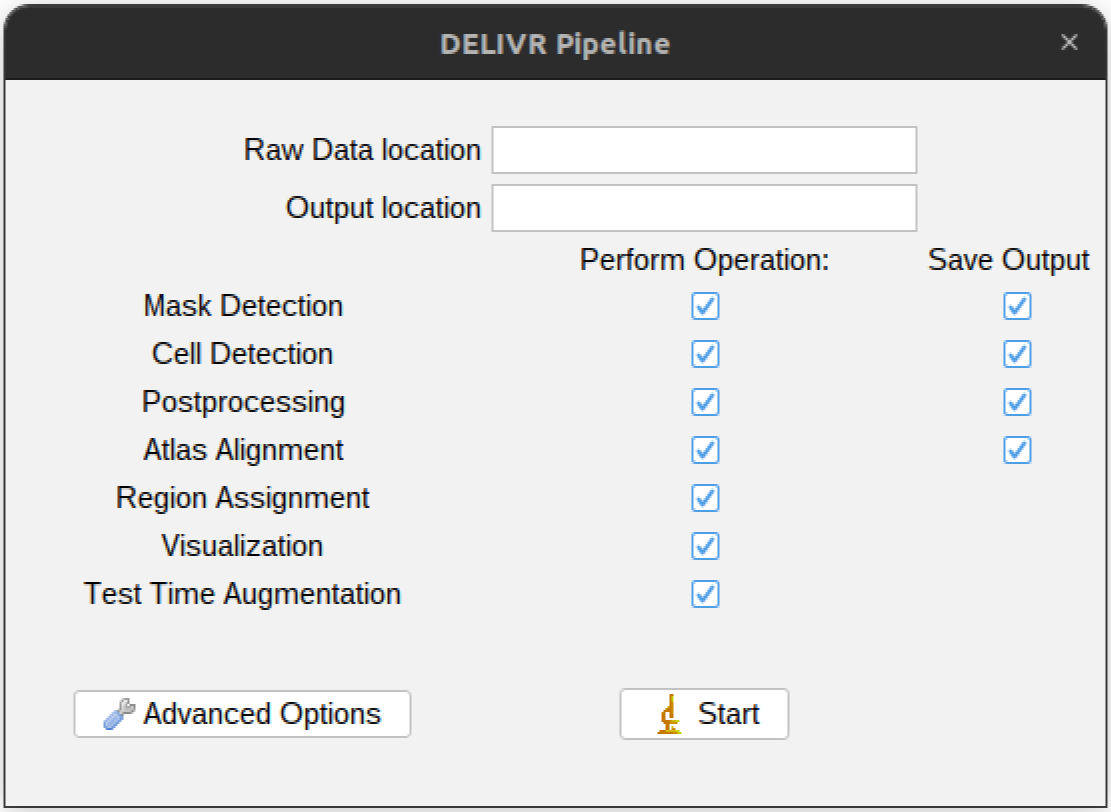

- The checkboxes indicate which operations the plugin will perform and are as follows:
    1. **Mask Detection**: Removes ventricles from the brain as well as skull stripping (removing non-brain area from the image).
    2. **Cell Detection**: Detects cell signals in the masked brain.
    3. **Postprocessing**: Performs a connected component analysis to count and mark individual cells.
    4. **Atlas Alignment**: Aligns the brain to the Allen Brain Atlas.
    5. **Region Assignment**: Assigns each cell detected in step 3 to a single region of the Allen Brain Atlas. Per brain, this function generates two tables (one per cell, and one with sums per brain area). It also generates a cell-distribution heatmap per brain and (optionally) per group.
    6. **Visualization**: Colors every cell detected in step 2 with the color assigned to a brain region in step 5 and outputs the results in the image space of the input image.
    7. **Test Time Augmentation:** Performs step 2 with test time augmentation enabled for a more accurate segmentation (around 5-7% increase in dice score) but at the cost of a 12 times longer segmentation time.

- For steps 1 to 4, select whether to save the intermediate results by checking “Save Output” or to discard them after a successful run by unchecking “Save Output”.
- To adapt the scaling to the correct resolution for your scan, click on **Advanced Options** and enter the X/Y/Z resolution of the scan, as well as the downsample properties for the mask detection. By selecting the **Model Locations** tab you can input different models for steps 1, 2, 4 and 5.

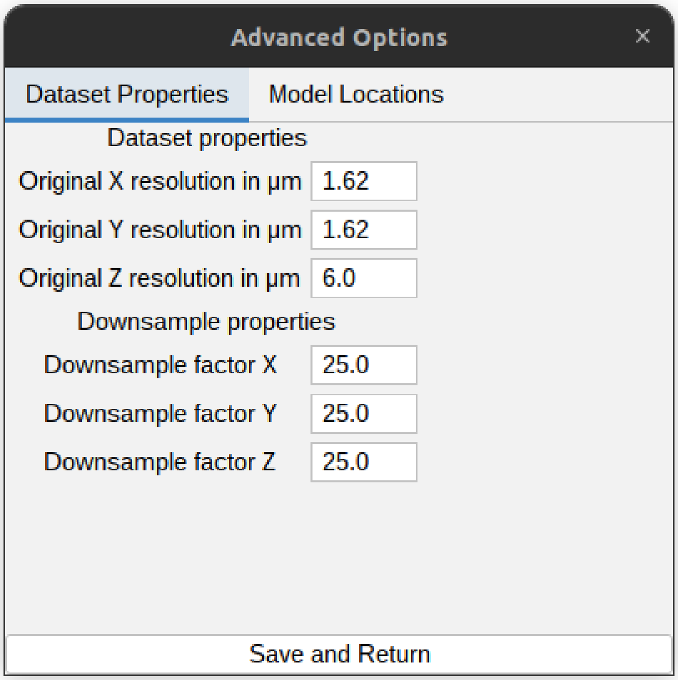

- After pressing start, the plugin will start the docker image, perform all the required operations and save the results in the output folder. Depending on the hardware and settings, this might take between 3 and 30 hours per brain.

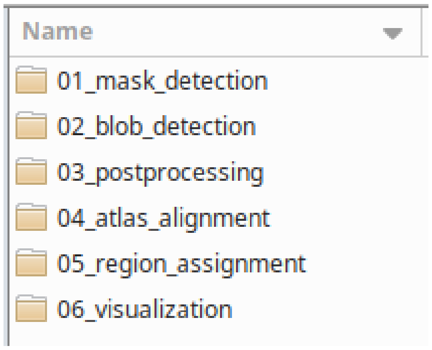

- The output folder contains 6 subfolders.
    - Depending on the "save output" checkboxes, intermediate results from the processing will be saved in folder 01_mask_detection, 02_blob_detection, 03_postprocessing, 04_atlas_alignment.
    - Folder 05_region_assignment will contain a heatmap per brain, plus two tables (one with information for each cell, and one with summed counts per brain region).
    - Folder 06_visualization will contain two subfolder per brain. One with a tiff image series with the same dimensions as the original microscope image stack, but with the cells colored in the respective area ID code of the Allen Brain Atlas. The other will be with cells color-coded according to their Allen Brain Atlas Region ID, for easy threshold-based region isolation in Fiji.
- Furthermore, if operation 5 Region Assignment is selected, it will automatically open the heatmaps of the processed brains once all processing is finished.

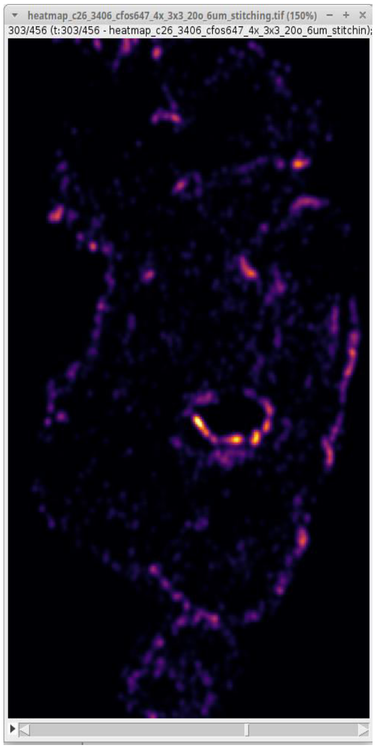

2.4.4 Validation of segmentation output in image space and atlas space

To visualize areas in a specific area, you can make use of the DELiVRs visualization output.

- If the "visualization" option is checked in the Fiji plugin, it will generate a "06_visualization" subfolder, which will contain one subfolder per brain.
    - DELiVR will then generate stacks of tif files, in the same dimensions as the original image stack, that contain all detected cells. Those are color-coded in one of two ways:

1) Color-coded as in the Allen Brain Atlas (8-bit RGB color tifs).

- Those color images are useful to sanity-check the segmentation performance per area. Fiji for 2D view: Open both raw images and RGB visualizations as virtual stacks (File -> Import -> Image Sequence, select folder, click "Load as virtual stack"). Synchronize the views with the Sync Windows function, then scroll through the stacks to get a side-by-side 2D comparison.
- Alternatively, convert this stack to a VR format in syGlass or Arivis VisionVR for an interactive 3D view similar to Supplementary Video 4. For a direct comparison with the raw data, convert the raw stack as well and merge them in the program as separate channels (e.g. RGB segmentation as R/G/B, raw as gray channel with 50% transparency).

2) Color-coded according to Allen Brain Atlas RegionID (32-bit single-channel tif).

- In the RegionID stack, the detected cells are labelled according to their RegionID code (refer to the Allen Brain Atlas ontology, "Atlas ID"). This makes it easy to select all neurons in a single region by thresholding in e.g. Fiji or syGlass.
- In Fiji, load a single z-plane (or image stack, though note that the 32-bit data will take up a lot of RAM), and go to "Image -> Adjust -> Threshold", then "Set" and enter the RegionID of the region you want to threshold for in both fields "minimum" and "maximum", then click "Apply".
- You can then combine this thresholded version with the raw data as an overlay: Load the raw data as image or stack, then go to Image -> Color -> Merge

Channels, then set e.g. masked cells as green and raw image as magenta (for max contrast) or gray.

2.4.5 Training DELiVR for your custom dataset using the Fiji Plugin

Our Fiji plugin contains a module to (re)train a network with your own data. One can retrain the provided cFos detection U-Net or train a completely new network from scratch.

2.4.5.1 Setup & Installation

Please download the training docker image and the Fiji plugin (if not done yet) from https://www.discotechnologies.org/DELiVR/ . For more information on the docker installation process please head to 2.3.1. To install the Fiji plugin, simply drop it into the Fiji "*Plugins*" folder.

2.4.5.2 Generation of training and testing data

- It is necessary to provide the docker for training with annotations of the signal you would like to segment with DELiVR in your image stack. For this, you can generate multiple smaller patches of your image stack (eg. 100x100x100 voxel patches) that are representative and annotate the signal in VR as described under “1. Annotation of ground truth data using virtual reality”. Alternatively, annotations can also be generated in 2D with softwares such as ITK-SNAP.
  - To evaluate how well the segmentation performs, it is essential that the testing patches will never be used in the training, as this can lead to an overfitting of the segmentation and misleading evaluations scores.
- The amount of training and testing data required can be individual for each data set and needs to be evaluated empirically.
  - As an example, for our c-Fos training, we annotated 48x 100x100x100 voxel patches of a c-Fos labeled and cleared mouse brain. This represented a total of 5889 cells. From these patches, we included 39 patches for the training. The remaining 9 patches were then used to test the segmentation performance.
- The raw image patches as well as the training/testing patches that include the annotations need to be saved in separate folders.
  - It is important that the raw and corresponding annotation files have the identical name, otherwise training docker will not recognize the pairing.

2.4.5.3 Data Setup

- The DELiVR training plugin requires a three folder set up as follows (the input text fields in Fiji are named accordingly):

- **Raw path**: A folder that contains the raw image files as nifti images.
- **GT path**: A folder that contains the annotations to the files in the raw path. Ensure that the file names are identical.
- **Output path**: Points to a folder where the output of the training is stored.

- Additionally, an optional path can be specified:
  - **Test path**: A folder that contains annotations to test patches to ensure consistent testing. Ensure that the corresponding raw files are saved in the raw path.
    - If the test path is empty, the DELiVR training feature will automatically assign a subset of patches for testing.
- To successfully train a model from scratch, a minimum of 20 labeled 100^3 patches is recommended. For retraining, depending on the complexity a range from 5 - 10 patches should be sufficient.

2.4.5.4 Starting the DELIVR Training via Fiji

- The Fiji Plugin contains the following options:
  - **Retrain**: Enable in order to retrain the original DELIVR cFos detection network, disable to train a network from scratch
  - **Test Time Augmentation**: Enable to apply test time augmentation for final testing
  - **Normalization**: Enable to normalize the input raw data using an intensity based normalization
  - **Epochs**: Training Epochs; how long the network trains; depends on the complexity of the problem. A fresh network can be trained for 300-800 epochs, retraining should be lower (200)
  - **Learning Rate**: Learning rate of the network, for a new network 0.001 is recommended, retraining should be lower (0.0001)
  - **Number of Workers**: Number of processes that load data for training, setting it to -1 will use all CPUs

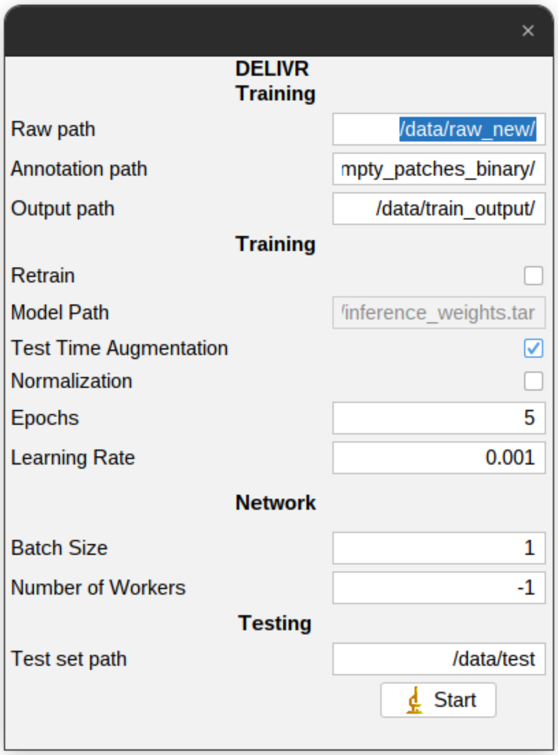

- After pressing start, the plugin will start the docker image, train the model, evaluate the model performance and save the results in the output folder. Depending on the training set size, hardware and settings, this might take between 1 and 20 hours.
- The output folder will be populated by copies of the raw and annotation path, as well as a path containing the training logs and trained models.
- After the training has completed, a popup will present the test scores of the model based on the test set. The test scores are saved in the test_scores.csv file in the output directory.
- The final model will be located in the output path as well, marked as *best_model*. and ending in *.ckpt*. In order to use this model, copy it into the output folder of 2.4.3, and in the inference plugin's "Advanced" options, write the path as "/data/output/MODEL_FILE" where MODEL_FILE is the name of the model file.

2.5 Troubleshooting DELiVR

For troubleshooting, FAQs and best practices, please refer to our wiki https://github.com/erturklab/delivr_cfos/wiki where we list common issues and constantly integrate feedback from the community.
