# Supplementary material for: Virtual reality-empowered deep-learning analysis of brain cells
Source: Nat Methods. 2024 Apr 22;21(7):1306–15. doi: 10.1038/s41592-024-02245-2 (PMC11239522; doi:10.1038/s41592-024-02245-2)
Supplement: Supplementary file 2 — Reporting Summary [file 41592_2024_2245_MOESM2_ESM.pdf]

nature portfolio

Corresponding author(s): Ali Ertürk, Mauricio Berriel Diaz

Last updated by author(s): 2024/03/05



# Reporting Summary

Nature Portfolio wishes to improve the reproducibility of the work that we publish. This form provides structure for consistency and transparency in reporting. For further information on Nature Portfolio policies, see our Editorial Policies and the Editorial Policy Checklist.

## Statistics

For all statistical analyses, confirm that the following items are present in the figure legend, table legend, main text, or Methods section.

| n/a | Confirmed | |
|---|---|---|
| ☐ | ☒ | The exact sample size (*n*) for each experimental group/condition, given as a discrete number and unit of measurement |
| ☐ | ☒ | A statement on whether measurements were taken from distinct samples or whether the same sample was measured repeatedly |
| ☐ | ☒ | The statistical test(s) used AND whether they are one- or two-sided<br>*Only common tests should be described solely by name; describe more complex techniques in the Methods section.* |
| ☒ | ☐ | A description of all covariates tested |
| ☐ | ☒ | A description of any assumptions or corrections, such as tests of normality and adjustment for multiple comparisons |
| ☐ | ☒ | A full description of the statistical parameters including central tendency (e.g. means) or other basic estimates (e.g. regression coefficient) AND variation (e.g. standard deviation) or associated estimates of uncertainty (e.g. confidence intervals) |
| ☐ | ☒ | For null hypothesis testing, the test statistic (e.g. *F*, *t*, *r*) with confidence intervals, effect sizes, degrees of freedom and *P* value noted<br>*Give P values as exact values whenever suitable.* |
| ☒ | ☐ | For Bayesian analysis, information on the choice of priors and Markov chain Monte Carlo settings |
| ☒ | ☐ | For hierarchical and complex designs, identification of the appropriate level for tests and full reporting of outcomes |
| ☒ | ☐ | Estimates of effect sizes (e.g. Cohen's *d*, Pearson's *r*), indicating how they were calculated |

*Our web collection on statistics for biologists contains articles on many of the points above.*

## Software and code

Policy information about availability of computer code

| | |
|---|---|
| Data collection | ImspectorPro [v 5.1] was used for collecting light-sheet images. |
| Data analysis | ClearMap (Renier et al, Cell, 2016), ClearMap2 (Kirst et al, Cell, 2020), Bio-Voxxel toolbox (https://doi.org/10.5281/zenodo.5986129), Python [v3.5 and v3.8], Ilastik (v1.4.0b8), Arivis VisionVR (v3.4.0), syGlass (v1.7.2), ITK-SNAP (v3.8), mBrainAligner (Qu et al, Nat. Methods, 2022), VeSSAP (Todorov et al, Nat. Methods, 2020), Allen Brain Atlas CCF3 atlas file as provided by the Scalable Brain Atlas (Bakker et al, Neuroinformatics, 2015), docker container (base: nvidia/cuda:11.7.2-runtime-ubuntu22.04), BrainRender (v2.0, Claudi et al., Elife 2020), cortical flatmap code (https://github.com/int-brain-lab/atlas) with adaptions (Negwer et al., Gigascience 2023), TeraStitcher portable (v1.11.10), PyTorch [v1.11], PyTorch Lightning [v2.0.5], Nibabel [v5.1.0], Monai [v1.2.0], Scipy [v1.8.1], Numpy [v1.24.4], Pandas [v1.4.3], , imglib2 [https://github.com/imglib/imglib2],cc3d (https://github.com/seung-lab/connected-components-3d), Fiji (v1.52p), Java (v1.8) Maven (v3.9.5), Jackson (https://github.com/FasterXML/jackson), GraphPad Prism (v9). Custom codes were used in the study for ventricle masking, cFos segmentation and quantification. The description is available in the Method section of the manuscript. The custom codes are publicly available on https://github.com/erturklab/delivr_cfos and https://github.com/erturklab/delivr_train |

For manuscripts utilizing custom algorithms or software that are central to the research but not yet described in published literature, software must be made available to editors and reviewers. We strongly encourage code deposition in a community repository (e.g. GitHub). See the Nature Portfolio guidelines for submitting code & software for further information.

## Data

Policy information about availability of data

All manuscripts must include a data availability statement. This statement should provide the following information, where applicable:

- Accession codes, unique identifiers, or web links for publicly available datasets
- A description of any restrictions on data availability
- For clinical datasets or third party data, please ensure that the statement adheres to our policy

Our code to run our pipeline end-to-end is available under https://github.com/erturklab/delivr_cfos/, training pipeline can be found at https://github.com/erturklab/delivr_train. The DeliVR docker image of the complete pipeline, the docker image for training, the Fiji plugin and a whole-brain test set can be downloaded through: https://www.discotechnologies.org/DELiVR/. Representative raw data scans (6 samples, 2 representative brain scans for each of the 3 conditions) is available at the European Bioimage Archive (Accession number: S-BIAD1019). Addtional raw data (26 brain scans, single-channel 16-bit tiff stacks) is available from the authors upon reasonable request. The Allen Brain Atlas (CCF3) was downloaded from the Scalable Brain Atlas repository: https://scalablebrainatlas.incf.org/mouse/ABA_v3.

## Human research participants

Policy information about studies involving human research participants and Sex and Gender in Research.

| | |
|---|---|
| Reporting on sex and gender | N/A |
| Population characteristics | N/A |
| Recruitment | N/A |
| Ethics oversight | N/A |

Note that full information on the approval of the study protocol must also be provided in the manuscript.

# Field-specific reporting

Please select the one below that is the best fit for your research. If you are not sure, read the appropriate sections before making your selection.

☒ Life sciences ☐ Behavioural & social sciences ☐ Ecological, evolutionary & environmental sciences

For a reference copy of the document with all sections, see nature.com/documents/nr-reporting-summary-flat.pdf

# Life sciences study design

All studies must disclose on these points even when the disclosure is negative.

| | |
|---|---|
| Sample size | Statistics were not used to predetermine sample sizes. Instead, we selected sample size based on previously conducted studies by us and others (Morigny et al., J Cachexia Sarcopenia Muscle. 2021 Oct;12(5):1333-1351; Xie et al., Proc Natl Acad Sci U S A. 2022 Mar 1;119(9):e2112840119.) These studies have demonstrated significant differences in cancer-induced weight loss and related parameters using the same models as employed in our current research. |
| Data exclusions | No animals were excluded from the study. We excluded cell counts detected in the fiber tracts of the brain from statistical anaylsis, as these do not contain neuronal cell bodies. |
| Replication | The antibody labeleig protocol, tissue-clearing, imaging, segmentation, mapping and quantification procedures were successfully performed on >30 mouse brains. |
| Randomization | Animals were not randomized. We grouped them based on their body weight to have experimental groups with similar starting body weights. Annotated patches for training and testing the deep learning and non-deep learning segmentation methods were randomized. Patches for VR vs 2D evaluation were chosen based on foreground signal. |
| Blinding | No blinding was done because knowledge of experimental conditions was required during animal handling and data collection. |

# Reporting for specific materials, systems and methods

We require information from authors about some types of materials, experimental systems and methods used in many studies. Here, indicate whether each material, system or method listed is relevant to your study. If you are not sure if a list item applies to your research, read the appropriate section before selecting a response.

## Materials & experimental systems

| n/a | Involved in the study |
|---|---|
| ☐ | ☒ Antibodies |
| ☐ | ☒ Eukaryotic cell lines |
| ☒ | ☐ Palaeontology and archaeology |
| ☐ | ☒ Animals and other organisms |
| ☒ | ☐ Clinical data |
| ☒ | ☐ Dual use research of concern |

## Methods

| n/a | Involved in the study |
|---|---|
| ☒ | ☐ ChIP-seq |
| ☒ | ☐ Flow cytometry |
| ☒ | ☐ MRI-based neuroimaging |

# Antibodies

| | |
|---|---|
| Antibodies used | c-Fos (9F6) Rabbit monoclonal antibody from Cell Signaling, Catalog #2250, 1:1000<br>Goat anti-Rabbit IgG (H+L) Highly Cross-Adsorbed Secondary Antibody, Alexa Fluor™ 647 from Thermo Fisher Scientific, Catalog # A-21245, 1:500<br>Atto647N-conjugated anti-GFP nanobooster, Chromotek, Catalog# gba647n-100, 1:1000 |
| Validation | Statements about c-Fos antibody validation can be found on the website of the manufacturer (https://www.cellsignal.de/products/primary-antibodies/c-fos-9f6-rabbit-mab/2250): The c-Fos antibody has been validated by Cell Signaling using SimpleChIP® Enzymatic Chromatin IP Kits. Species reactivity is determined by testing in at least one approved application (eg. Western Blot).<br>Statements about the anti-GFP nanobooster can be found on the website of the mannfacurer (https://www.ptglab.com/products/GFP-Booster-ATTO647N-gba647n.htm#product-information): The nanobodies are validated by a genetic approach. They are tested on cell lines that express and do not express GFP. |

# Eukaryotic cell lines

Policy information about cell lines and Sex and Gender in Research

| | |
|---|---|
| Cell line source(s) | C26 were provided by tumor bank of the German Cancer Research Center (Deutsches Krebsforschungszentrum, DKFZ Tumorbank). NC26 cells were kindly provided by Rudolf Zechner and Martina Schweiger from the Institute of Molecular Biosciences (University of Graz) and were originally obtained from the Cell Resource Center for Biomedical Research-Cell Bank of the Tohoku University (TKG-0518). |
| Authentication | The cell lines have not been authenticated. |
| Mycoplasma contamination | Cell lines have undergone myoplasma checks and tested negative. |
| Commonly misidentified lines<br>(See ICLAC register) | No commonly misidentified cell lines were used. |

# Animals and other research organisms

Policy information about studies involving animals; ARRIVE guidelines recommended for reporting animal research, and Sex and Gender in Research

| | |
|---|---|
| Laboratory animals | We used male BALB/c mice at an age of 10-12 weeks and 6-8 week old CX3CR1 GFP/+ (B6.129P-Cx3cr1tm1Litt/J (Jackson Laboratory strain code 005582). |
| Wild animals | We did not use wild animals. |
| Reporting on sex | We used only used male mice in our study to keep experimental conditions maintain consistency in experimental conditions and minimize variability. This decision is grounded in the recognition of reported distinctions in cancer-associated weight loss between male and female cancer patients, with the former exhibiting a more pronounced susceptibility. In mouse models, sex-specific regulations may influence the response to cachexia-inducing cytokines such as IL-6 during the progression of cachexia. Consequently, employing only male mice enhances the precision of our investigation by aligning with the potential sex-specific nuances in disease phenotypes, thereby contributing to a more focused and insightful analysis |
| Field-collected samples | The study did not involve samples collected from the field. |
| Ethics oversight | Animal experimentation was performed in accordance with the European Union directives and the German animal welfare act (Tierschutzgesetz). They have been approved the state ethics committee and the government of Upper Bavaria. |

Note that full information on the approval of the study protocol must also be provided in the manuscript.
